# Chemical Profiling of *Commiphora gileadensis*, *Salsola incanescens*, and *Savignya parviflora* and Their Protective Effects in an Acute Rat Model of Ulcerative Colitis

**DOI:** 10.3390/ph19081178

**Published:** 2026-07-27

**Authors:** Fawaz K. Alanazi, Nashwa Hashad, Asmaa A. Ahmed, Elsayed K. El-Sayed, Yara E. Mansour, Mohamed I. S. Abdelhady, Eman G. Haggag, Fatma M. Abdel Bar

**Affiliations:** 1 Department of Pharmacognosy, Faculty of Pharmacy, Capital University (Formerly Helwan University), Cairo 11795, Egypt; ph.fawaz@hotmail.com (F.K.A.); nashwa.elsayed@pharm.capu.edu.eg (N.H.); mohamed_abdelhady@pharm.capu.edu.eg (M.I.S.A.); eman.g.haggag@pharm.capu.edu.eg (E.G.H.); 2 Department of Pharmacology and Toxicology, Faculty of Pharmacy, Capital University (Formerly Helwan University), Cairo 11795, Egypt; dr.asmaa_ali@pharm.capu.edu.eg (A.A.A.); elsayed_elsayed@pharm.capu.edu.eg (E.K.E.-S.); 3 Department of Pharmaceutical Chemistry and Pharmacognosy, College of Pharmacy, Qassim University, Unaizah 51911, Saudi Arabia; y.mansour@qu.edu.sa; 4 Pharmaceutical Organic Chemistry Department, Faculty of Pharmacy, Capital University (Formerly Helwan University), Cairo 11795, Egypt; 5 Department of Pharmacognosy, College of Pharmacy, Prince Sattam Bin Abdulaziz University, Al-Kharj 11942, Saudi Arabia; 6 Department of Pharmacognosy, Faculty of Pharmacy, Mansoura University, Mansoura 35516, Egypt

**Keywords:** ulcerative colitis, TLR4, *Commiphora gileadensis*, *Salsola incanescens*, *Savignya parviflora*, LC-MS/MS

## Abstract

**Background/Objectives**: Ulcerative colitis (UC) is a relapsing colonic disorder in which persistent immune-mediated inflammation is accompanied by oxidative imbalance and deterioration of the protective intestinal mucosal barrier. Due to disease complexity and limitations of current therapies, alternative and adjunctive treatments are needed. This study investigated the phytochemical profiles and anti-UC activities of *Commiphora gileadensis*, *Salsola incanescens*, and *Savignya parviflora*. **Methods**: The methanolic extracts of aerial parts (AMEs) were characterized using LC-MS/MS and evaluated for their protective effects against acetic acid (AA)-induced UC in female *Sprague Dawley* rats. Animals were treated orally once daily for 14 days with extract doses of 250 and 500 mg/kg prior to colitis induction. In vitro anti-inflammatory and NO scavenging activities were also evaluated. Molecular docking was performed to explore the potential mechanisms of action by predicting the interactions of key compounds with COX-1 and 2 and the TLR4/MD-2 complex. **Results**: LC-MS/MS analysis revealed flavonoid-rich extracts with characteristic metabolites, including triterpenes in *C. gileadensis*, phenolic amides in *S. incanescens*, and amino acids in *S. parviflora*. *C. gileadensis* exhibited the strongest in vitro anti-inflammatory activity, showing marked suppression of interleukin-6 and tumor necrosis factor-α production in lipopolysaccharide (LPS)-induced RAW264.7 macrophages, selective COX-2 inhibition, and potent NO scavenging activity comparable to celecoxib. The extracts significantly attenuated AA-induced colonic injury in a dose-dependent manner. *C. gileadensis* at 500 mg/kg exhibited the highest protective efficacy by reducing disease activity index, colonic edema, oxidative stress, and inflammatory mediators, while restoring mucosal architecture. It downregulated TLR4 and attenuated NF-κB-dependent inflammatory and iNOS pathways. Histopathological examination and mucin expression results were consistent with these findings. Molecular docking was utilized as a hypothesis-generating tool to provide an in silico insight into the anti-inflammatory activity of *C. gileadensis*, suggesting potential multi-target interactions with COX-1, COX-2, and the TLR4/MD-2 complex, likely attributable to its unique triterpenoid and flavonoid constituents. **Conclusions**: *C. gileadensis* emerged as the most promising extract for UC management, exhibiting marked antioxidant and anti-inflammatory effects. Further mechanistic, pharmacokinetic, and clinical investigations are required to establish its translational potential for the treatment of UC.

## 1. Introduction

Ulcerative colitis (UC) is a chronic, idiopathic, and relapsing inflammatory bowel disease (IBD) that primarily affects the mucosal lining of the colon. This inflammatory disease causes several symptoms affecting the quality of the patient’s life, including significant abdominal pain, diarrhea, rectal bleeding, and weight loss [1]. While the exact etiology remains indefinable, UC is widely considered to arise from a multifactorial interaction between personal genetic susceptibility, immune response dysregulation, environmental factors, and gut microbiota imbalances. Among the UC experimental models, the acetic acid-induced UC model in rats is one of the most widely used chemically induced models of acute colitis, exhibiting high reproducibility and reproducing several pathological features observed in human ulcerative colitis, including epithelial erosion, mucosal ulceration, neutrophil infiltration, oxidative stress, and excessive production of inflammatory mediators. Furthermore, acetic acid-induced colonic injury is strongly associated with activation of the oxidative and inflammatory signaling pathways, making this model particularly suitable for evaluating the anti-inflammatory, antioxidant, and mucosal protective effects of the investigated plant extracts and for assessing the molecular mechanisms explored in the present study [2,3,4].

A central pathway involved in UC pathogenesis is the activation of Toll-like receptor 4 (TLR4)/interleukin-1 receptor-associated kinase (IRAK)/nuclear factor kappa B (NF-κB) signaling axis. Activation of TLR4 by gut microbial or tissue damage-associated molecular patterns (MAMPs or DAMPs) leads to the recruitment of IRAK, which in turn activates NF-κB, a transcription factor that orchestrates the cellular expression of various pro-inflammatory cytokines and chemokines. Sustained activation of this pathway amplifies intestinal inflammation and contributes to the chronicity of the disease. In parallel, oxidative stress plays a pivotal role in disease progression through the overproduction of reactive oxygen species (ROS) by the inflammatory cells, which not only damage cellular components but also further activate inflammatory pathways. Moreover, excessive oxidative stress can disrupt the redox balance, impair antioxidant defences, and trigger apoptosis of epithelial cells, compromising mucosal barrier integrity and exacerbating inflammation [5,6].

Despite advances in pharmacological management, current therapeutic agents for UC often carry significant side effects and fail to induce long-term remission in many patients [1,7]. Consequently, there is growing interest in exploring alternative and plant-based therapies that offer anti-inflammatory, antioxidant, and anti-apoptotic properties, potentially providing safer and more effective strategies for adjuvant or additive therapies in UC management.

Recently, the increased tendency to introduce herbal formulations into modern medical practices is attributed to their relative availability and low cost compared with conventional treatments and the common feeling of safety and minimal side effects of natural plants [8]. This concept encouraged scientists to strengthen the bond between traditional uses of common plants and scientific evidence by conducting their studies on different models.

*Commiphora gileadensis* (L.) C.Ch. (syn. *C. opobalsamum*) is a small to medium shrub, which belongs to the family Burseraceae [9]. It is distributed all over the Middle East in diverse environments and is known in Arabic as Mecca myrrh, Ood-e-balsan, Balm of Mecca, or Balsam [10,11]. In traditional Arabic medicine, decoctions and other oral preparations of different parts of *C. gileadensis* were used in the management of gastrointestinal conditions, including stomach troubles, colic, constipation, and jaundice, as well as hepatobiliary conditions. Also, in traditional Chinese medicine and Ayurveda, orally or topically applied bark-derived resins were used for gastrointestinal disorders [11,12].

Plants of the *Salsola* genus (Amaranthaceae), commonly known as saltwort or Russian thistle, are halophytes capable of living in saline environments and are widely distributed in temperate regions, representing nearly 45% of desert plants, native to Africa, Europe, and Asia [13,14]. They have a documented history in traditional medicine for treating various gastrointestinal (GIT) ailments [14,15], and recent research provides scientific support for some of these uses. For instance, *S. imbricata* is traditionally reputed for managing GIT issues such as indigestion, vomiting, and abdominal discomfort. Experimental studies confirm its antispasmodic effects on intestinal smooth muscle, mediated by calcium antagonism and *β*-adrenergic agonism, which help relieve spasms and support its use for indigestion and abdominal bloating. Additionally, *S. imbricata* extract has shown protective effects in animal models of inflammatory bowel disease, reducing inflammation, oxidative stress, and tissue damage in the colon, which aligns with traditional uses for conditions like hemorrhoids and dyspepsia. *S. collina* is also recognized in traditional medicine for treating constipation and has demonstrated the ability to promote gastrointestinal motility in animal studies, supporting its use for digestive sluggishness and bloating. These findings provide a pharmacological basis for the traditional use of *Salsola* species in managing a range of GIT ailments, including indigestion, vomiting, hemorrhoids, dyspepsia, and abdominal bloating. *Salsola incanescens* (syn. *Caroxylon incanescens* (C.A.Mey.) Akhani & Roalson) is a hairy, erect, perennial herbaceous plant with fleshy and juicy leaves belonging to the Amaranthaceae family. It reaches 70 cm in height and may cover an area of 1.5 m [16]. It is known locally as Al-khithraf. It has been found to have little reported research on its phytochemical constituents and biological activities.

*Savignya parviflora* (Delile) Webb (syn. *Farsetia parviflora* (Delile) Spreng., *Lunaria parviflora* Delile, *Savignya aegyptiaca* DC.) is an annual herb that belongs to the mustard family (Brassicaceae) and is native to the Middle East and North Africa. It is known in Arabic as Gilgilan, Klikelan, Qulayqalan (or Qalqal) due to the distinct, clicking noise or rattling sound produced by its mature fruits, like the “qalqala” sound in the Arabic language. Plants of the family Brassicaceae are well known in traditional medicine for managing a wide range of gastrointestinal ailments, including gastritis, cholecystolithiasis, constipation, and liver diseases [17,18]. Basically, Brassicaceae is known for its richness in sulfur-containing compounds (Glucosinolates), which have antimicrobial properties that can help combat *Helicobacter pylori*, a leading cause of ulcers [19].

Recent research has increasingly endorsed a multi-target treatment approach for IBD, where effective natural products simultaneously diminish inflammatory signaling, reduce oxidative stress, and aid in the restoration of the intestinal mucosal barrier. The TLR4/NF-κB pathway has been identified as a key regulator of colon inflammation, with various natural metabolites, including flavonoids, terpenoids, and phenolic compounds, demonstrated to inhibit TLR4-mediated activation of downstream kinases like IRAK, leading to a decrease in the production of pro-inflammatory mediators and the expression of COX-2 and iNOS [20,21,22,23]. Simultaneously, recent findings underscore the significance of antioxidant defenses and mucosal repair in securing lasting protection against experimental colitis. Natural phenolics and flavonoids have been reported to activate Nrf2-associated antioxidant pathways, enhance endogenous enzymes such as SOD and CAT, restore goblet cell populations, and improve mucin secretion and epithelial integrity [24,25]. Furthermore, several natural products have demonstrated the ability to repair the intestinal barrier by modulating TLR4-dependent signaling and preserving tight junction integrity [26]. Despite these advances, the selective inhibition of inflammatory enzymes, particularly COX-2, by natural products remains poorly explored in experimental colitis models. Moreover, although numerous flavonoid-rich plants have been investigated, the phytochemical composition and anti-colitic potential of Arabian desert medicinal plants remain largely unexplored. Therefore, investigating the phytochemical profiles and protective effects of *C. gileadensis*, *S. incanescens*, and *S. parviflora* may provide novel insights into multi-target natural therapies for ulcerative colitis.

This work aimed to investigate the phytochemical composition of the methanolic extracts of three selected plants native to the Arab region, *C. gileadensis*, *S. incanescens*, and *S. parviflora*, using LC–MS/MS-based profiling; assess their protective effects against acetic acid-induced ulcerative colitis in rats; explore the underlying mechanisms of action through evaluating their cyclooxygenase-1 and 2 (COX-1, and 2) inhibitory, nitric oxide (NO) scavenging activities, as well as their effects on pro-inflammatory cytokine production in LPS-stimulated RAW264.7 macrophages, and the modulation of the TLR4/IRAK/NF-κB signaling pathway; and conduct molecular docking analyses to assess the interactions of the identified phytoconstituents with TLR4, COX-1, and COX-2 as potential anti-inflammatory targets.

## 2. Results

### 2.1. Phytochemical Profiling by LC-MS/MS Analysis

Metabolite annotation was classified according to the conceptual values defined by Schymanski et al. [27]. In the absence of authentic reference standards (Level 1; Confirmed/Standard), metabolites were classified as probable based on unambiguous library matches or diagnostic fragmentation patterns, or as tentative/putative for class-level assignments or those exhibiting high mass errors (>±10 ppm) [27]. The annotated phytochemical profiles of the methanolic extracts of the aerial parts (AME) of *C. gileadensis*, *S. incanescens*, and *S. parviflora*, identified by LC-MS/MS in positive and negative ionization modes, are presented in Tables S1–S3. A comparative phytochemical profile of the major bioactive and marker metabolites tentatively identified in the three extracts is summarized in Table 1.

#### 2.1.1. Phytochemical Profiling of *C. gileadensis*

**Figure 1:**
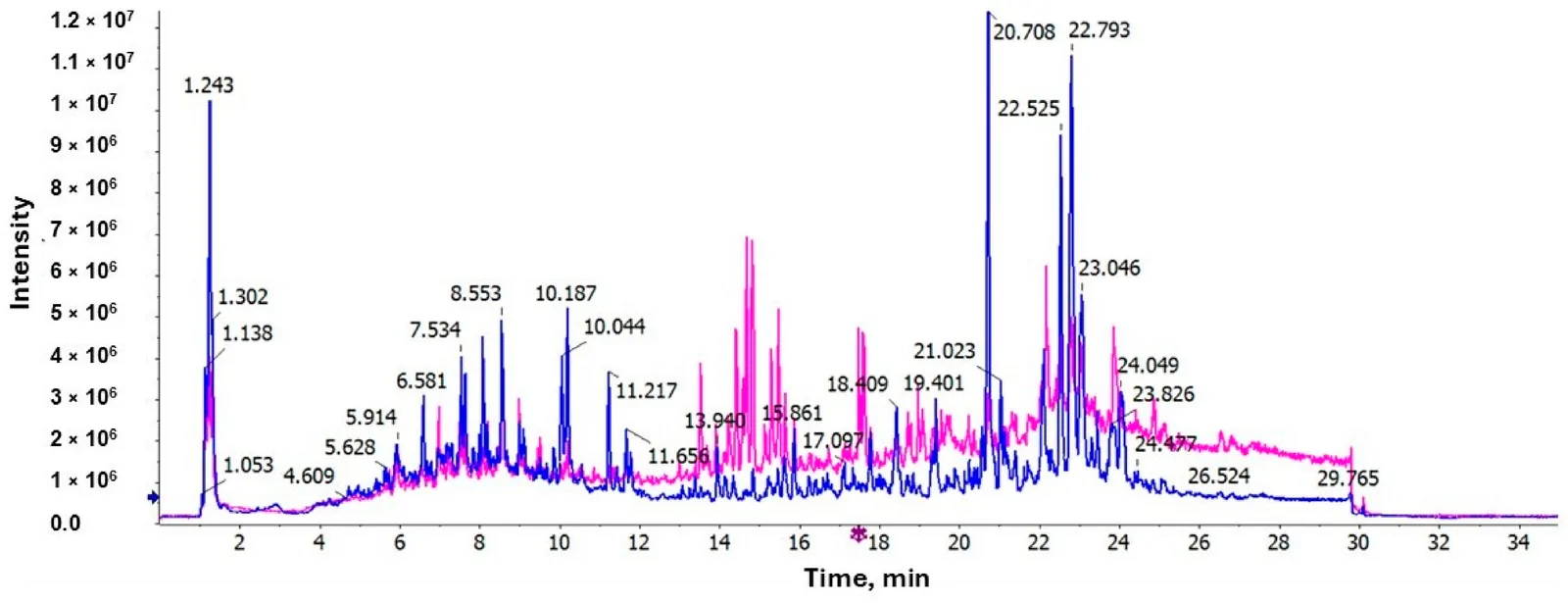
Total ion chromatograms (TICs) from LC-MS analysis of *C. gileadensis*, acquired in both negative ionization mode (blue) and positive ionization mode (pink).

A total of 59 belonging to ten chemical classes were tentatively identified from the LC-MS/MS analysis of *C. gileadensis* extract, particularly the unique triterpenes and phenolic derivatives (Figures S1–S14). Flavonoids and their glycosides constituted a major group of the identified compounds (27 compounds), followed by triterpenes and sterols (9 compounds), simple phenolic derivatives (5 compounds), fatty acids and carboxylic acids and their derivatives (4 compounds each), coumarins and amino acids (3 compounds each), sugars and stilbenes (1 compound each), and miscellaneous group (2 compounds) (Figure 1 and Table S1).

Notably, the major identified group of compounds, flavonoids, is represented in different subclasses, including flavonols as the major subclass, followed by flavones, flavanones, chalcone derivatives, and anthocyanins, occurring mainly as *O*-glycosides and aglycones, with fewer *C*-glycosides.

#### 2.1.2. Phytochemical Profiling of *S. incanescens*

**Figure 2:**
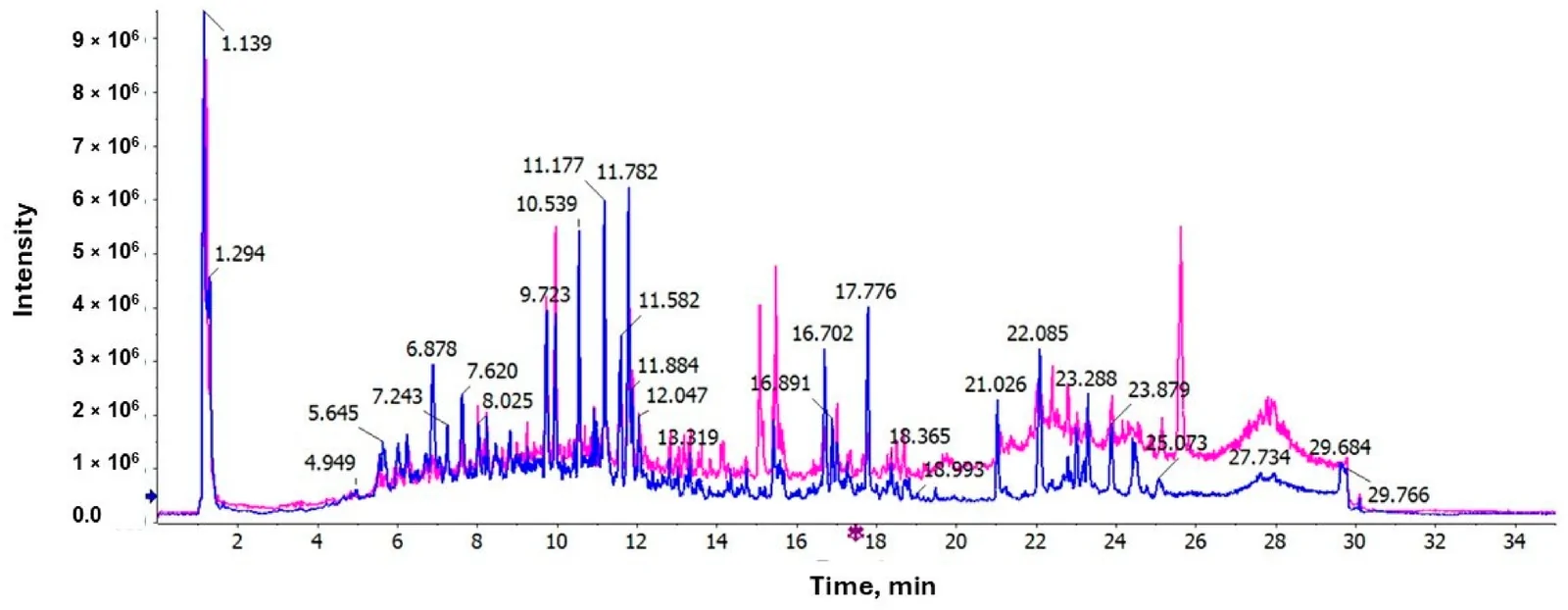
Total ion chromatograms (TICs) from LC-MS analysis of *S. incanescens*, acquired in both negative ionization mode (blue) and positive ionization mode (pink).

LC–MS/MS analysis tentatively identified 69 compounds belonging to ten chemical classes from *S. incanescens* extract, with alkaloids and nitrogenous compounds (Figures S15–S19) and flavonoids and their glycosides representing the major groups of the identified compounds **(17** and **16** compounds), followed by coumarins, stilbenes, phenolic acids and miscellaneous carboxylic acids (8 compounds each), nucleobases and triterpenoid derivatives (**7** and **5** compounds), amino acid and their derivatives (3 compounds), oligosaccharides and sugar derivatives, sterols and fatty acid derivatives (2 compounds each), and vitamins (1 compound) (Figure 2 and Table S2).

#### 2.1.3. Phytochemical Profiling of *S. parviflora*

**Figure 3:**
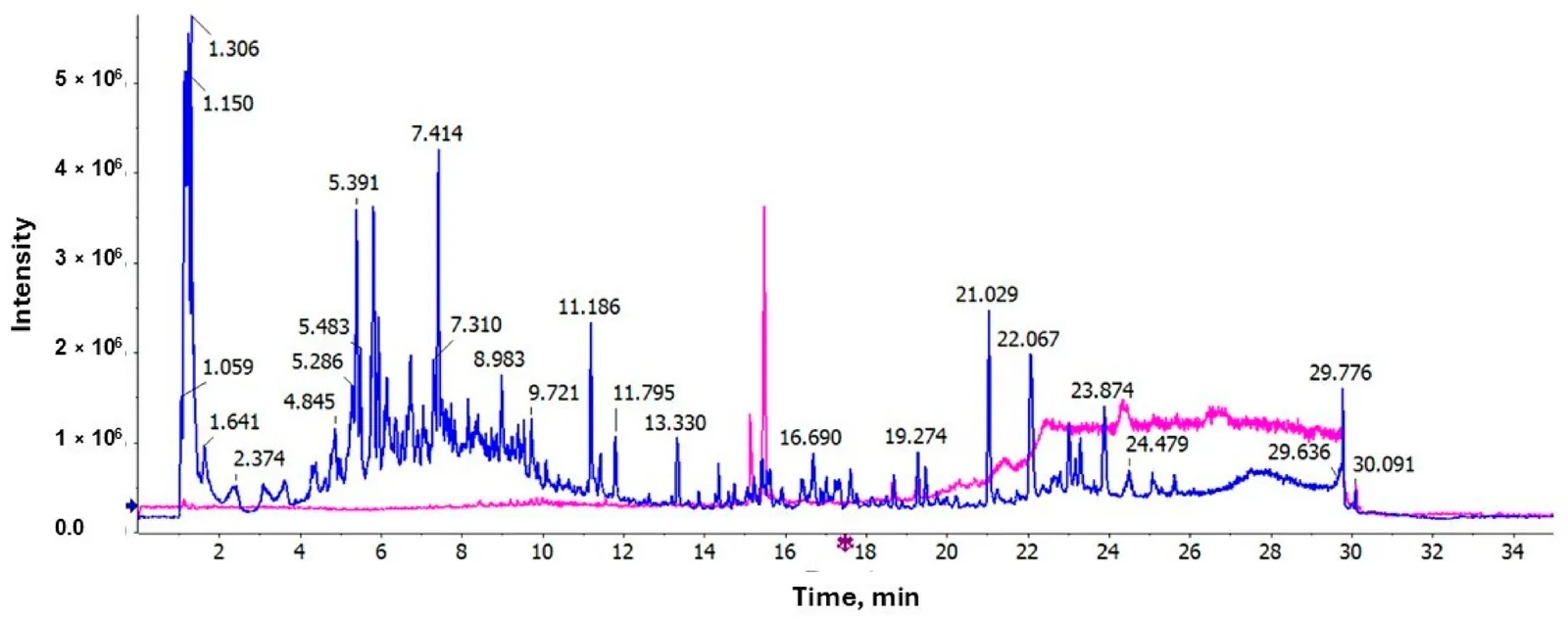
Total ion chromatograms (TICs) from LC-MS analysis of *S. parviflora*, acquired in both negative ionization mode (blue) and positive ionization mode (pink).

From *S. parviflora* extract, a total of 41 compounds belonging to eight chemical classes were tentatively identified, with amino acids and flavonoids and their glycosides comprising the major groups of compounds (14 and 12 compounds), followed by fatty acids and their derivatives (5 compounds), carboxylic acids (3 compounds), phenolic acids, alkyl amides (Figures S20 and S21), and glycerophospholipids (2 compounds each), and nucleosides (1 compound) (Figure 3, Table S3).

### 2.2. Total Phenolic and Flavonoid Contents

The calibration curves of gallic acid and quercetin showed good linearity within the tested concentration ranges (Figures S22 and S23) and were used for quantification of total phenolic content (TPC) and total flavonoid content (TFC). Quantitative data are summarized in Table 2, while the detailed absorbance and calculation data are provided in Tables S4 and S5.

Among the extracts investigated, *S. parviflora* exhibited the highest TPC, followed by *C. gileadensis*, whereas *S. incanescens* showed the lowest value. In contrast, the highest TFC was detected in *C. gileadensis*; however, when calculated per gram of dry plant material, *S. parviflora* yielded the highest total flavonoid content (2.45 mg QE/g) due to its significantly higher extraction yield.

### 2.3. In Vitro Anti-Inflammatory and Antioxidant Activities

#### 2.3.1. Assessment of IL-6 and TNF-α Levels in RAW264.7 Macrophages

LPS stimulation of RAW264.7 macrophages induced a significant increase in IL-6 and TNF-α secretion, consistent with the well-established role of LPS in activating Toll-like receptor 4 (TLR4) signalling and downstream cytokine production. *C. gileadensis* reduced IL-6 and TNF-α levels by approximately 89% and 84%, respectively, compared with the LPS control, with an efficacy comparable to celecoxib. This suggests that *C. gileadensis* may act upstream of cytokine production, potentially by interfering with TLR4/NF-κB signaling. *S. incanescensis* showed considerable inhibition; meanwhile, *S. parviflora* showed the least inhibitory effect (Table 3).

#### 2.3.2. Assessment of COX-1 and COX-2 Enzymes Inhibition

Among the examined extracts, *C. gileadensis* exhibited the highest selectivity for COX-2 over COX-1 (SI = 4.44). *S. incanescensis* exhibited weak selectivity (SI = 1.74), while *S. parviflora* exhibited non-selective inhibition (SI = 1.33). As expected, celecoxib exhibited highly selective COX-2 inhibition (SI = 55.33), consistent with its defined pharmacological profile (Table 4). These findings suggest that *C. gileadensis* may simultaneously suppress cytokine production and inhibit COX-2. This dual mechanism may be beneficial in managing complex inflammatory diseases in which both prostaglandin synthesis and cytokine cascades are involved in disease pathology.

#### 2.3.3. Assessment of Nitric Oxide Radical Scavenging Activity

As presented in Table 4, *C. gileadensis* demonstrated potent NO radical scavenging activity with an IC_50_ of 27.31 µg/mL, comparable with the IC_50_ of celecoxib (37.92 µg/mL). *S. incanescensis* showed moderate NO scavenging (IC_50_ = 43.35 µg/mL), while *S. parviflora* exhibited the least activity (IC_50_ = 52.24 µg/mL). The observed effects go in parallel with the results seen in cytokine suppression, suggesting a possible relationship between radical scavenging capacity and anti-inflammatory efficacy. The NO scavenging ability of *C. gileadensis* may complement its COX-2 inhibitory and cytokine-suppressing activities, providing a multi-faceted approach for its possible therapeutic activity.

### 2.4. In Vivo Anti-Ulcerative Colitis Activities in AA-Induced UC in Rats

#### 2.4.1. Acute Toxicity Study

The Acute oral toxicity evaluation of the AME of *C. gileadensis*, *S. incanescens*, and *S. parviflora* revealed no mortality or treatment-related signs of toxicity at oral doses up to 5 g/kg during the 24 h observation period. The animals exhibited normal behavioral and motor activities throughout the study. Based on these findings and previous literature supporting the pharmacological efficacy of plant extracts within this dose range, 250 and 500 mg/kg were selected as the experimental doses for the subsequent in vivo UC study.

These doses are consistent with previous pharmacological investigations of Commiphora gileadensis and are within the dose range commonly employed for evaluating the biological activities of medicinal plant extracts, including members of the genus Salsola, to investigate dose-dependent pharmacological effects [14,28,29,30].

#### 2.4.2. Effect of AMEs on Disease Activity Index (DAI)

**Figure 4:**
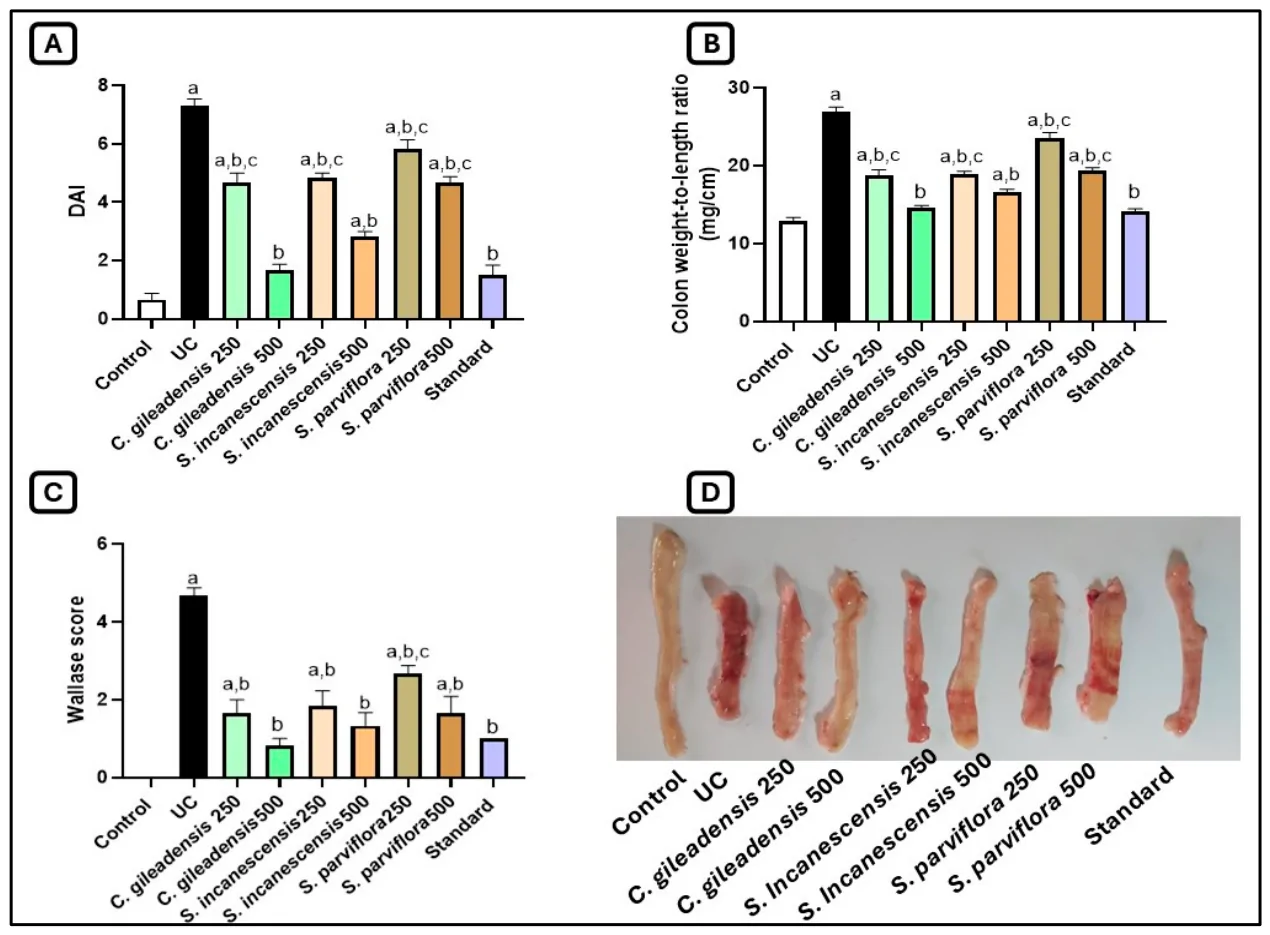
Effect of the tested AMEs of *C. gileadensis*, *S. incanescens*, and *S. parviflora* on (**A**) DAI, (**B**) Colon weight-to-length ratio, (**C**) Colon macroscopic score (Wallase score), and (**D**) Photographic representation of the colonic tissues. Data represented as mean ± SEM. *n* = 6. a: significant from the control group at *p* < 0.05, b: significant from the UC group at *p* < 0.05, c: significant from the standard group at *p* < 0.05.

The determined DAI showed a significant (*p* < 0.05) increase in the UC group by 11-fold compared with the control group. Meanwhile, treatment with sulfasalazine as a standard caused a significant (*p* < 0.05) 79.5% decrease in the DAI compared with the UC group. Treatment with 250 mg/kg of *C. gileadensis*, *S. incanescens*, and *S. parviflora* caused a significant (*p* < 0.05) decrease in DAI by 36.4%, 34.1, and 20.5%, respectively. On the other hand, treatment with 500 mg/kg of *C. gileadensis*, *S. incanescens*, and *S. parviflora* caused a significant (*p* < 0.05) decrease in DAI by 77.3%, 61.4%, and 36.4%, respectively (Figure 4A). The highest protective activity was recorded for *C. gileadensis* at a dose of 500, as it showed a nonsignificant difference in DAI compared with the sulfasalazine standard group.

#### 2.4.3. Effect of AMEs on Colon Weight-to-Length Ratio

IR injection of AA caused a notable swelling and shrinkage of the colonic tissue, indicated by the significant (*p* < 0.05) 2.1-fold increase in colon weight-to-length ratio compared with the control group. Treatment with 250 mg/kg of *C. gileadensis*, *S. incanescens*, and *S. parviflora* significantly (*p* < 0.05) decreased the ratio by 30.3%, 29.7%, and 12.8%, respectively, comparable to the UC group. Moreover, the dose of 500 mg/kg of *C. gileadensis*, *S. incanescens*, and *S. parviflora* significantly (*p* < 0.05) decreased the ratio by 45.7%, 38.2%, and 28.3%, respectively, comparable to the UC group (Figure 4B). The highest inhibitory effect was recorded with 500 mg/kg of *C. gileadensis*, as it is comparable with the effect of the standard sulfasalazine, which caused a 47.8% decrease in the weight-to-length ratio.

#### 2.4.4. Effect of AMEs on Colon Macroscopic Score

AA injected intrarectally induced tissue edema, inflammation, and ulceration of the mucosa, causing a significant (*p* < 0.05) 4.7-fold higher macroscopic Wallas score, compared with the control group. Meanwhile, 250 mg/kg of *C. gileadensis*, *S. incanescens*, and *S. parviflora* tissue samples revealed less tissue inflammation and ulceration, with a significant (*p* < 0.05) decrease in Wallas macroscopic scoring by 64.3%, 60.7%, and 42.9%, respectively, comparable with the UC group. The doses of 500 mg/kg of *C. gileadensis*, *S. incanescens*, and *S. parviflora* significantly (*p* < 0.05) decreased the score by 82.1%, 71.4%, and 64.3%, respectively, comparable to the UC group (Figure 4C,D). The highest inhibitory effect was recorded with 500 mg/kg of *C. gileadensis*, as it is comparable with the effect of the standard sulfasalazine, which caused a 78.6% decrease in the weight-to-length ratio.

#### 2.4.5. Effect of AMEs on Oxidative Stress Parameters

**Figure 5:**
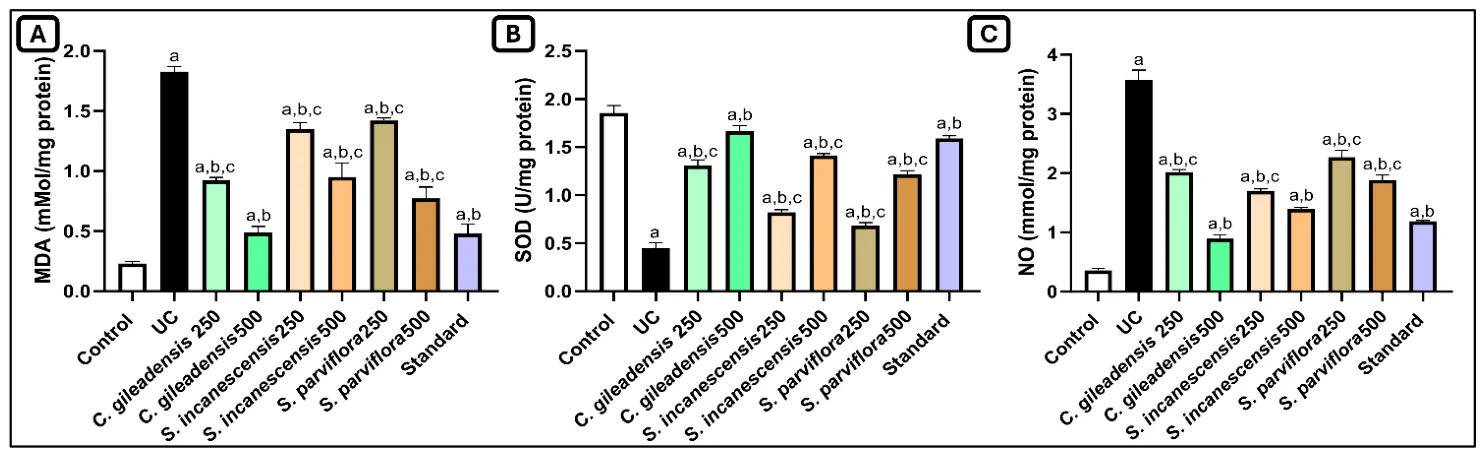
Effect of the tested AMEs of *C. gileadensis*, *S. incanescensis*, and *S. parviflora* on (**A**) MDA, (**B**) SOD, and (**C**) NO. Data represented as mean ± SEM. *n* = 6. a: significant from the control group at *p* < 0.05, b: significant from the UC group at *p* < 0.05, c: significant from the standard group at *p* < 0.05. MDA: malondialdehyde, SOD: superoxide dismutase, NO: nitric oxide.

Treatment with AA intrarectally caused significant (*p* < 0.05) colonic oxidative tissue damage, evidenced by the 7.9-fold increase in MDA content, 10-fold increase in NO content, and 75.9% decrease in SOD content, compared with the control group. Sulfasalazine caused a significant (*p* < 0.05) 73.6% decrease in MDA content, 66.7% decrease in NO content, and 3.6-fold increase in SOD content, compared with the UC group. Treatment with *C. gileadensis*, *S. incanescens*, and *S. parviflora* exhibited significant antioxidant activities in a dose-dependent-matter as the dose of 250 mg/kg of the extracts caused a significant decrease in MDA content by 49.4%, 26.1%, and 22.2%, respectively; decrease in NO content by 43.6%, 52.4%, and 36.5%, respectively; and increase in SOD content by 2.9, 1.8, and 1.5 folds, respectively, compared with the UC group. Meanwhile, the dose of 500 mg/kg of the extracts caused a significant decrease in MDA content by 73.3%, 48.0%, and 57.6%, respectively; a decrease in NO content by 74.9%, 60.9%, and 47.3%, respectively; and an increase in SOD content by 3.7, 3.2, and 2.7 folds, respectively, compared with the UC group. The highest antioxidant activity was recorded for *C. gileadensis* at the 500 mg/kg dose, which is nonsignificant from the standard sulfasalazine group (Figure 5).

#### 2.4.6. Effect of AMEs on Inflammatory Markers

**Figure 6:**
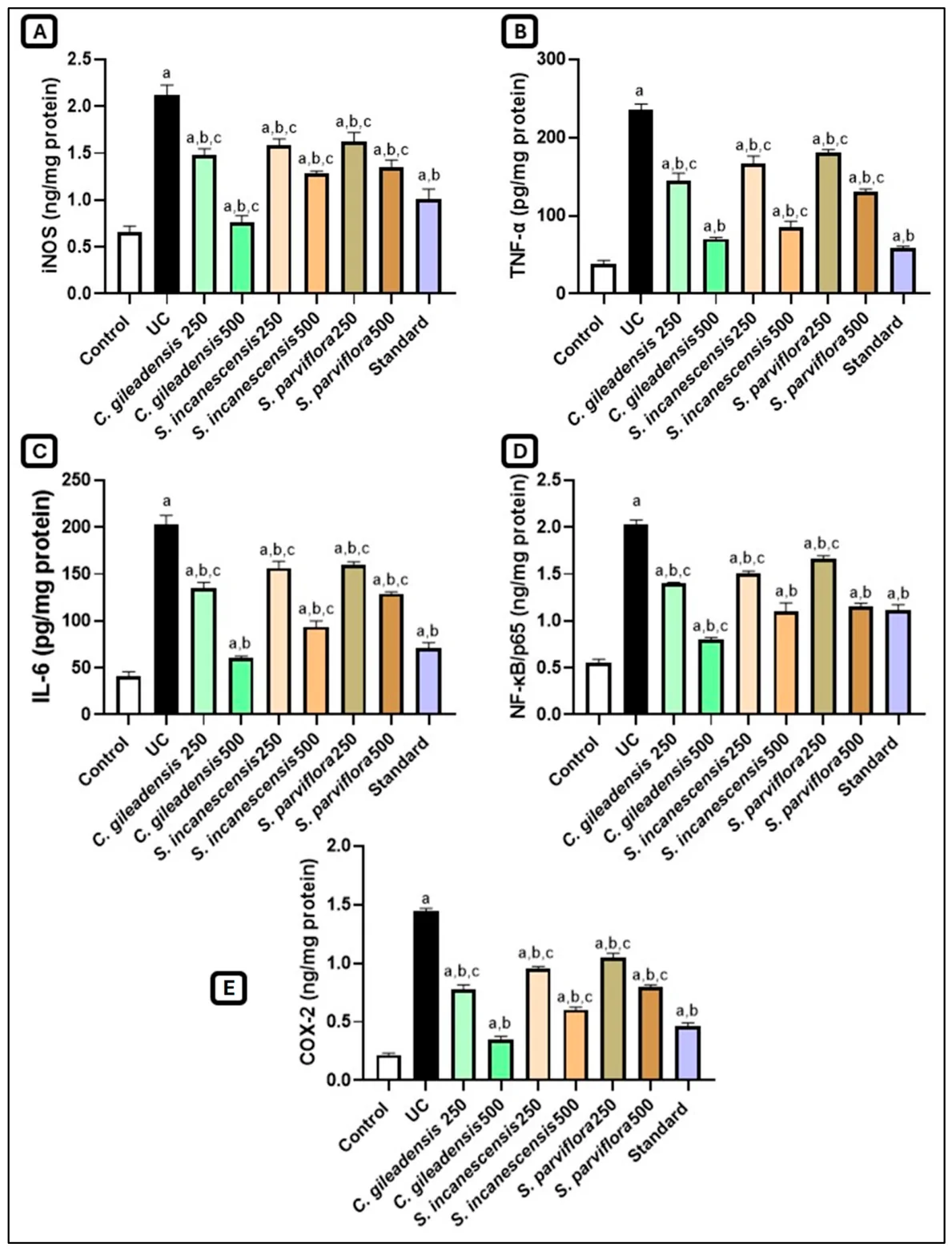
Effect of the tested AMEs of *C. gileadensis*, *S. incanescensis*, and *S. parviflora* on (**A**) iNOS, (**B**) TNF-α, (**C**) IL-6, (**D**) NF-ĸB/p65, and (**E**) COX-2. Data represented as mean ± SEM. *n* = 6. a: significant from the control group at *p* < 0.05, b: significant from the UC group at *p* < 0.05, c: significant from the standard group at *p* < 0.05. iNOS: inducible nitric oxide synthase, TNF-α: tumor necrosis factor-α, IL-6: interleukin-6, NF-ĸB/p65: phosphorylated nuclear factor-kappa B, COX-2: cyclooxygenase-2.

As demonstrated in Figure 6, AA-induced UC is characterized by consequential inflammatory damage, evidenced by the significant (*p* < 0.05) increase in iNOS (3.2-fold), TNF-α (6.1-fold), IL-6 (5-fold), NF-κB p65 (3.6-fold), and COX-2 (6.6-fold). Meanwhile, treatment with the standard caused a significant (*p* < 0.05) decrease in iNOS (53%), TNF-α (75.1%), IL-6 (64.7%), NF-κB p65 (45.3%), and COX-2 (67.8%). Treatment with *C. gileadensis*, *S. incanescens*, and *S. parviflora* exhibited significant anti-inflammatory effect in a dose-dependent manner as the dose of 250 mg/kg of the extracts caused a significant (*p* < 0.05) decrease in iNOS (30.2%, 24.9%, and 23.2%), TNF-α (38.6%, 29.0%, and 22.8%), IL-6 (33.2%, 22.6%, and 21.0%), NF-κB p65 (31.0%, 25.6%, and 18.1%), and COX-2 (45.8%, 34.0%, and 27.3%) compared with the UC group. Meanwhile, the dose of 500 mg/kg of the extracts caused further significant (*p* < 0.05) decrease in iNOS (63.8%, 39.2%, and 35.9%), TNF-α (70.0%, 63.6%, and 44.1%), IL-6 (70.0%, 53.6%, and 36.2%), NF-κB p65 (60.3%, 45.9%, and 43.1%), and COX-2 (75.7%, 58.1%, and 44.7%) compared with the UC group. The highest activity is observed for *C. gileadensis* at a dose of 500 mg/kg, which exhibited anti-inflammatory activity comparable to the standard group.

#### 2.4.7. Effect of AMEs on Prostaglandin E2 (PGE2)

**Figure 7:**
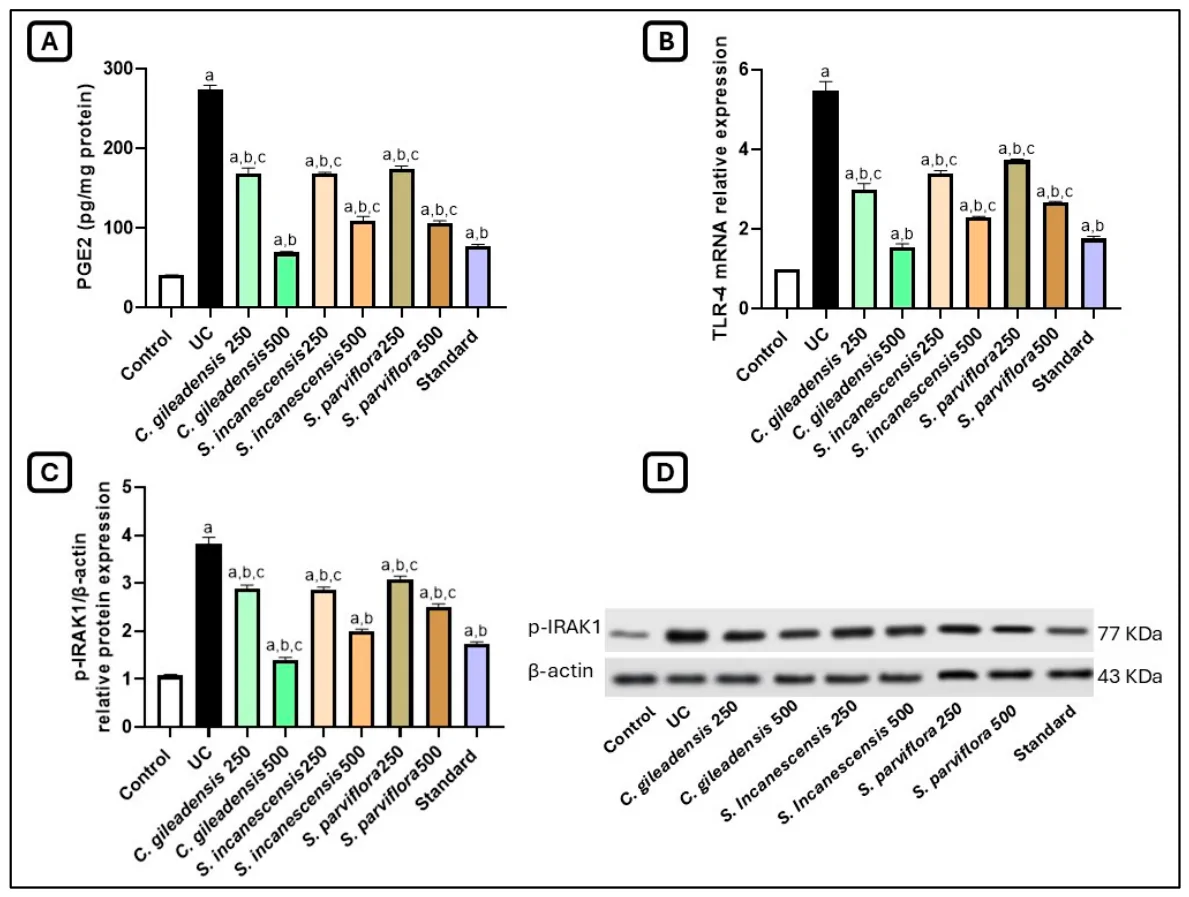
Effect of the tested AMEs of *C. gileadensis*, *S. incanescensis*, and *S. parviflora* on (**A**) PGE2, (**B**) TLR4 relative gene expression, (**C**) p-IRAK1 relative protein expression, and (**D**) Representative cropped Western blot images showing the expression of p-IRAK1 with β-actin as a loading control. Data represented as mean ± SEM. *n* = 6. a: significant from the control group at *p* < 0.05, b: significant from the UC group at *p* < 0.05, c: significant from the standard group at *p* < 0.05. PGE2: prostaglandin E2, TLR4: toll like receptor-4, p-IRAK1: phosphorylated interleukin-1 receptor-associated kinase.

As represented in Figure 7A, IR administration of AA caused a significant (*p* < 0.05) increase in PGE2 level by 6.8-fold, compared with the control group. Treatment with *C. gileadensis*, *S. incanescens*, and *S. parviflora* exhibited a significant decrease in PGE2 in a dose-dependent manner, as the dose of 250 mg/kg of the extracts caused a significant (*p* < 0.05) reduction by 38.3%, 38.3%, and 36.6%, respectively, compared with the UC group. Meanwhile, a dose of 500 mg/kg of the extracts caused a further significant (*p* < 0.05) decrease in PGE2 levels by 74.7%, 60.3%, and 61.5%, respectively, compared with the UC group. The highest effect is observed for *C. gileadensis* at a dose of 500 mg/kg, as it is nonsignificant from the standard group.

#### 2.4.8. Effect of AMEs on TLR4 and p-IRAK1

Induction of UC by AA caused a significant increase in the colonic tissue expression of TLR4 by 5.5-fold, with a subsequent increase in phosphorylation of its downstream kinase IRAK1 by 3.6-fold, compared with the control group. Meanwhile, treatment with *C. gileadensis*, *S. incanescens*, and *S. parviflora* at a dose of 250 mg/kg caused a significant (*p* < 0.05) decrease in TLR4 expression by 45.3%, 38.3%, and 32.1%, respectively, and in p-IRAK1 by 24.7%, 25.3%, and 19.4%, respectively, compared with the AA-treated group. The higher dose of 500 mg/kg of the extracts and the standard caused a further significant (*p* < 0.05) reduction in TLR4 expression by 71.9%, 58.2%, 51.5%, and 67.9%, respectively, and p-IRAK1 by 63.4%, 47.8%, 34.5%, and 54.7%, respectively, compared with the AA-treated group. As presented in Figure 7B–D, the highest effect is observed for *C. gileadensis* at a dose of 500 mg/kg, as it is nonsignificant from the standard group regarding TLR4 expression level, and it showed a significant decrease in p-IRAK1 expression level by 19%, compared with the standard group.

#### 2.4.9. Effect of AMEs on Histopathological Examination

**Figure 8:**
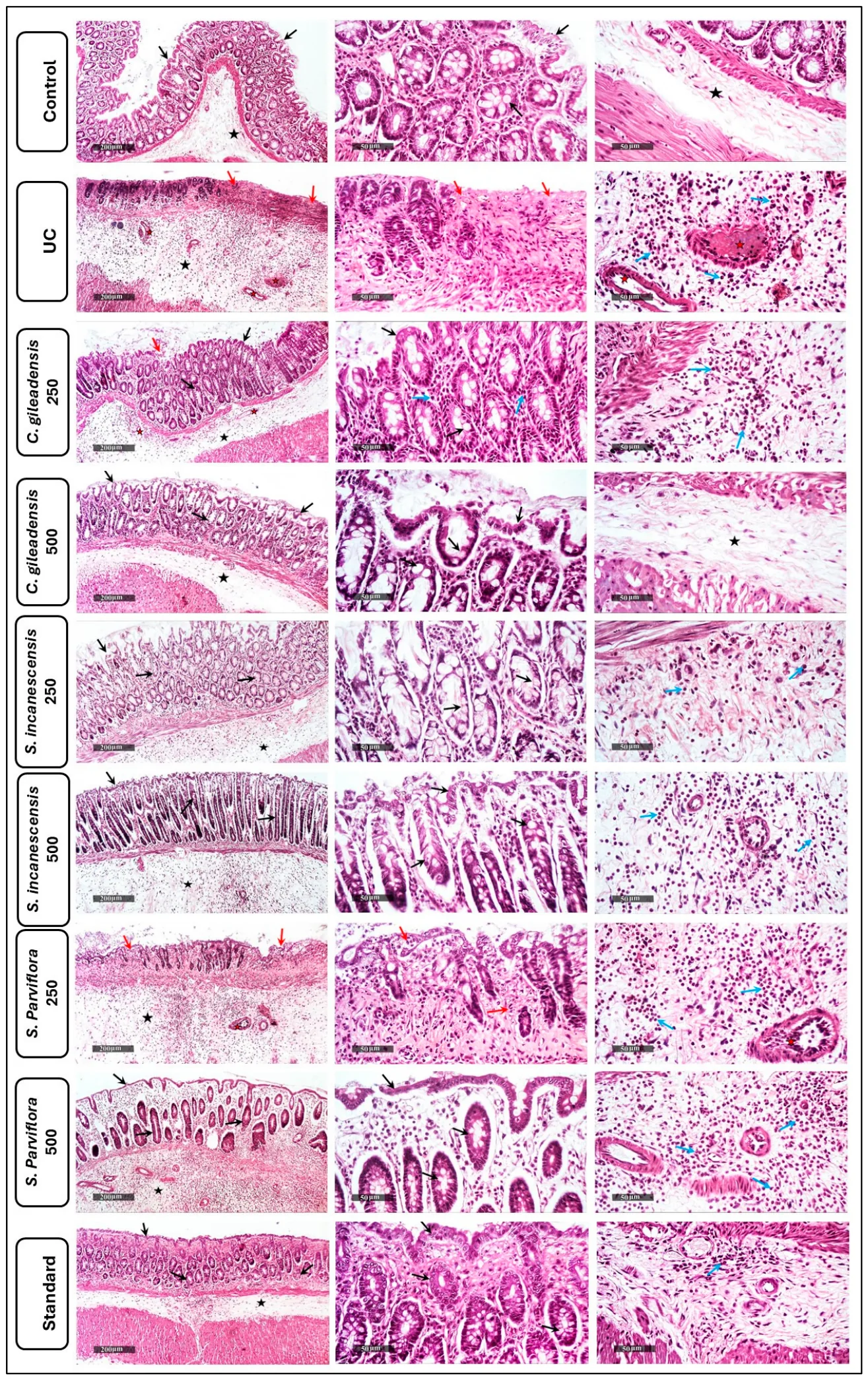
Effect of the tested AMEs of *C. gileadensis*, *S. incanescensis*, and *S. parviflora* on the histopathological findings (H&E staining). The control group showed normal architecture with intact crypts and goblet cells (black arrow) and preserved submucosa (black star). The UC group exhibited severe ulceration, necrotic depressions (red arrow), inflammatory infiltrates (blue arrow), vascular congestion (red star), and oedema (black star). *C. gileadensis* 250 provided moderate protection with focal erosions (red arrow), re-epithelialisation (black arrow), but persistent inflammation (blue arrow) and oedema (black star). *C. gileadensis* 500 showed marked improvement with intact epithelium, increased goblet cells (black arrow), minimal inflammation, and mild oedema (black star). *S. incanescens* 250 and 500 demonstrated moderate improvement with intact glands and goblet cells (black arrow), but persistent inflammation (blue arrow) and oedema (black star). *S. parviflora* 250 showed minimal efficacy (similar to UC). *S. parviflora* 500 showed moderate glandular protection but severe inflammation. The standard treatment showed efficacy comparable to *C. gileadensis* 500 with mild focal inflammation (blue arrow).

As presented in Figure 8, microscopic examination of rat colon samples from the control group demonstrated a well-organized morphological architecture. Apparent intact colonic crypts showed numerous goblet cells (black arrow) with a maintained overlying epithelial lining layer. The submucosa (star) and outer muscular coat also preserved their normal structure. Meanwhile, samples from the UC group revealed a substantial loss of the colonic wall’s normal architecture, marked by ulcerative hemorrhagic colitis, abundant mucosal necrotic depressions (red arrow), significant loss of glandular structures, moderate to severe mixed inflammatory infiltrates in the mucosa and submucosa (blue arrow), congested blood vessels (red star), and pronounced submucosal edema (black star). Samples from the *C. gileadensis* 250-treated group demonstrated moderate protective efficacy with persistent focal records of mucosal superficial erosions and loss of lining epithelium (red arrow) with abundant figures of glandular structure re-epithelialization (black arrow). However, a limited number of mature goblet cells were identified, along with persistent severe mucosal/submucosal infiltrates of mixed inflammatory cells (blue arrow), moderately congested blood vessels (red star), and submucosal edema (black star). Colonic samples from the *C. gileadensis* 500-treated group revealed marked improvement in the histological organization, with a clearly intact lining epithelium and more preserved mucosal glandular structures, alongside a notable increase in mature goblet cells (black arrow). In most examined samples, minimal mucosal/submucosal inflammatory cell infiltrates were observed, with mild persistent submucosal edema (black star). Moreover, *S. incanescens* 250-treated group samples showed moderate improvement in colonic mucosal organization, with higher records of intact lining epithelium and mucosal glandular structures, along with moderate mature goblet cells (black arrow). However, persistent moderate aggregates of inflammatory cells were observed in the colonic wall (blue arrow), as well as edema (black star) with minor congested blood vessels. *S. incanescens* 500-treated group samples showed similar records to *S. incanescens* 250-treated group samples. Colonic samples from the *S. parviflora* 250-treated group exhibited almost the same records as UC group samples, with minimal protective efficacy among all the treated groups. Samples from the *S. parviflora* 500-treated group showed equivalent protective efficacy on colonic glandular structures as *S. incanescens* samples; however, more severe and persistent figures of reactive inflammatory cell infiltrates were observed. Standard-treated samples showed relatively comparable protective efficacy to *C. gileadensis* 500 samples. However, persistent mucosal superficial loss of glandular structures was observed with mild focal inflammatory cell infiltrates (blue arrow).

#### 2.4.10. Effect of AMEs on Relative Mucin Expression

**Figure 9:**
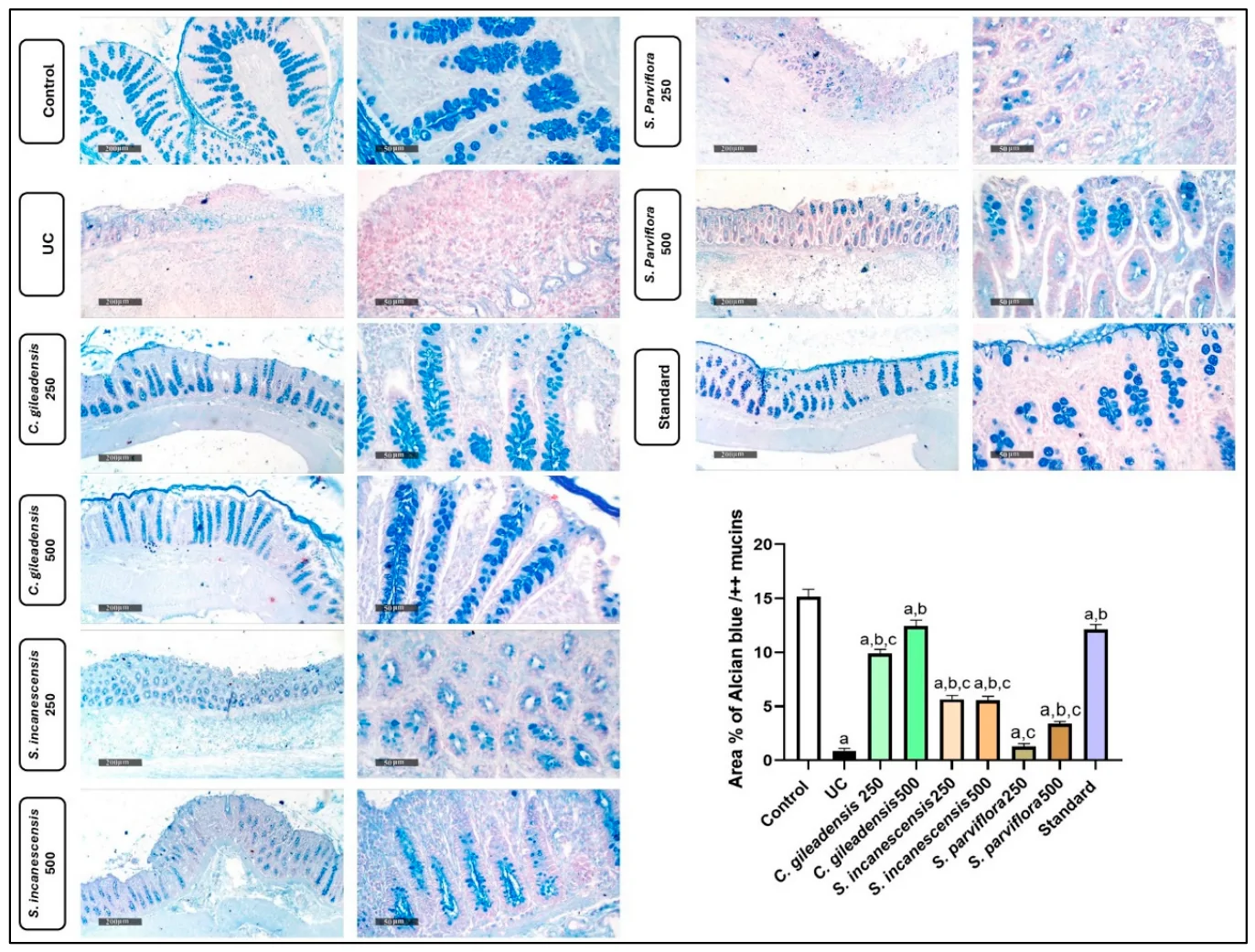
Effect of the tested AMEs of *C. gileadensis*, *S. incanescensis*, and *S. parviflora* on mucin expression using Alcian Blue staining. Data represented as mean ± SEM. *n* = 6. a: significant from the control group at *p* < 0.05, b: significant from the UC group at *p* < 0.05, c: significant from the standard group at *p* < 0.05.

Induction of UC by AA caused remarkable disruption of the mucus barrier, indicated by the significant (*p* < 0.05) decrease in the relative mucin expression measured immunochemically by 94%, compared with the normal control group (Figure 9). Treatment with *S. parviflora* 250 showed a similar result to the UC group. Meanwhile, treatment with *C. gileadensis* and *S. incanescens* at a dose of 250 mg/kg caused a significant (*p* < 0.05) increase in the relative mucin expression by 11-fold and 6.2-fold, respectively, compared with the UC group. Higher doses of 500 mg/kg of *C. gileadensis*, *S. incanescens*, and *S. parviflora*, and the standard caused a significant (*p* < 0.05) increase in the relative mucin expression by 13.8-, 6.2-, 3.7-, and 13.4-fold, respectively, compared with the UC group. Accordingly, the highest protective effect is observed for *C. gileadensis* at a dose of 500 mg/kg, as it is nonsignificant from the standard group.

### 2.5. In Silico Molecular Docking Evaluation of the Major Identified Compounds

Molecular docking studies were conducted to correlate the anti-inflammatory activity of the three extracts of *C. gileadensis*, *S. incanescens*, and *S. parviflora* with potential targets, including TLR4, COX-1, and COX-2 (PDB ID: 2Z65, 1Q4G, and 3NT1). To validate the protocol, we redocked the crystallized ligands Eritoran, Fluprofen, and Naproxen (E55, BFL, and NPS) into the TLR4, COX-1, and COX-2 active sites, respectively. The redocked poses yielded RMSDs of 1.59, 0.99, and 1.17 Å, with binding scores of −10.12, −7.899, and −7.66 kcal/mol.

Using the validated protocol, selected key phytocompounds identified from the plant species investigated were docked into the binding pocket of PDB ID: 2Z65, 1Q4G, and 3NT1. For TLR4, the large LPS-binding pocket resulted in diverse binding patterns (Table S6 and Figure S24), with the top affinities shown in Table 5. For COX-1 and COX-2, visual inspection of the superposed poses shows that all compounds occupy the canonical binding cavity and substantially overlap the BFL and NPS binding cavity, indicating consistent targeting residues critical for COX-1 and COX-2 inhibition. The highest binding affinities are recorded in Table 6.

## 3. Discussion

### 3.1. Phytochemical Profiling by LC-MS/MS Analysis

#### 3.1.1. Phytochemical Profiling of *C. gileadensis*

The LC-MS/MS analysis of *C. gileadensis* revealed a very rich phytochemical profile with diverse classes.

Compound **1.1.3** (R_t_ 6.92 min, *m*/*z* 481.0960) was assigned as a probable identification of gossypin. The MS^2^ spectrum showed a prominent base peak at *m*/*z* 319.0422 (calculated *m*/*z* 319.0448), corresponding to the loss of a glucose moiety [M – glucose-H]^−^ (MSBNK-RIKEN-PR100353) (Table S1, Figure S1) [31]. To the best of our knowledge, this is the first putative report of this compound in *C. gileadensis* and requires confirmation using an authentic reference standard.

Likewise, several flavonoid glycosides were detected in the MS^2^ spectrum (negative mode), showing a prominent peak corresponding to the loss of a sugar moiety [M - H - sugar]^−^, along with other diagnostic fragment ions (Table S1, Figures S2–S6). Compound **1.1.13** (*m*/*z* 611.1856) was tentatively assigned as neohesperidin dihydrochalcone (Figure S2) [31,32]. However, the relatively high mass error (–20.5 ppm), exceeding the accepted ±10 ppm threshold, limits confidence in this annotation. Therefore, to the best of our knowledge, this is reported as a putative first identification in *C. gileadensis* and requires confirmation. Isorhamnetin nucleus represented by isorhamnetin pentoside (**1.1.21**; Figure S3) [33], isorhamnetin-3-*O*-glucoside (**1.1.12**) [34], isorhamnetin-3-*O*-rutinoside (**1.1.15**) [34,35], along with their aglycone, isorhamnetin (**1.1.25**) [31,34]. Other key detected flavonoids in *C. gileadensis* included delphinidin-3-*O*-beta-glucopyranoside (**1.1.6**) [36], compounds with acacetin nucleus, such as **1.1.1** [32] and **1.1.27** [37], and luteolin nucleus, such as **1.1.9** [31,34] and **1.1.19** [31,38].

This is also the first potential report of kaempferol-3-*O*-alpha-L-rhamnoside (**1.1.17**; Figure S4) *C. gileadensis* [31,34]. Similarly, compounds **1.1.8** [34], **1.1.16** [31], and **1.1.26** [31,32,35], which share the kaempferol nucleus, as well as apigenin 8-C-glucoside (**1.1.5**) [39] and hyperoside (**1.1.7**) [31], were potentially reported herein for the first time from this species. In contrast, their corresponding aglycones, kaempferol, apigenin (**1.1.22**) [31], and quercetin (**1.1.20**) [31,32], were previously reported from *C. gileadensis* [30]. Quercitrin (**1.1.10**; Figure S5) [32] was also reported for the first time from *C. gileadensis*, although it has previously been identified in *C. leptophloeos* [40]. Likewise, okanin-4′-*O*-glucoside (**1.1.2**) [32] is newly reported from *C. gileadensis*, whereas its aglycone, okanin, was previously isolated from *C. mollis* [41]. On the other hand, hesperidin (**1.1.4**; Figure S6) [31], taxifolin (**1.1.11**) [37], myricetin (**1.1.14**) [36], eriodictoyl (**1.1.18**) [31], naringenin (**1.1.23**) [31,36], and hesperetin (**1.1.24**) were previously reported from *C. gileadensis* [30,42]. Notably, certain flavonoids (**1.1.9**, **1.1.15**, **1.1.19**, **1.1.20**, **1.1.23**, and **1.1.26**) were detected in both negative and positive ionization modes (Table S1).

Triterpenoids constituted one of the main phytochemical classes identified in *C. gileadensis* [30]. A probable identification of commigileadin A (**1.9.5**, *m*/*z* 455.351) was based on diagnostic MS^2^ fragments (Figure S7), involving the loss of a water molecule (*m*/*z* 437.3353; [M + H − H_2_O]^+^) (calculated *m*/*z* 437.3414) and a formic acid moiety (*m*/*z* 409.3402; [M + H − HCOOH]^+^). Commigileadin A is a friedelan triterpenoid that was previously isolated from *C. gileadensis* aerial parts [42].

Compound **1.9.7** (*m*/*z* 441.3727) was assigned a probable identification based on the MS^2^ fragmentation pattern (Figure S8) in the positive mode, showing a prominent base peak at *m*/*z* 423.3574, corresponding to the loss of a hydroxyl moiety [M + H − H_2_O]^+^. Canophyllal is a friedelan triterpenoid that was previously isolated from *C. gileadensis* aerial parts and stems [43,44], and it is the first proposal of the MS^2^ fragmentation pattern of the compound.

Compounds **1.9.2**, **1.9.3**, **1.9.6**, and **1.9.8** [45] were tentatively identified as triterpenoids and are reported here for the first time from *C. gileadensis*, although other structurally related triterpenoids have previously been reported from this species [30].

Among the phenolic acid derivatives, chlorogenic acid (**1.2.1**) was identified in both positive and negative ionization modes (Figures S9 and S10) and is reported herein for the first time from *C. gileadensis* [31,32]. Likewise, sinapyl aldehyde (**1.2.4**; Figure S11) [31,32] and vanillic acid glucoside (**1.2.2**) [46], are newly identified from this species. Notably, the corresponding oxidized derivative of sinapyl aldehyde, sinapic acid, has previously been reported from *C. gileadensis* [30]. In addition, p-coumaric acid (**1.2.5**; Figure S12) [31,32] was detected herein for the first time in *C. gileadensis*. Although this compound was previously reported from *C. africana* [47], its saturated analogue, phloretic acid, was reported before from *C. gileadensis* [48].

In addition, a stilbene compound (**1.4.1**; Figure S13) [31,32], two coumarins (**1.3.2** and **1.3.3**) [34], and two fatty acids, 12-oxo-10,15(*Z*)-phytodienoic acid (**1.6.1**; Figure S14) [31] and suberic acid (**1.6.2**) [31,32], were identified and reported herein for the first time from *C. gileadensis* (Table S1).

#### 3.1.2. Phytochemical Profiling of *S. incanescens*

The LC-MS analysis of *S. incanescens* revealed a rich and diverse phytochemical profile comprising alkaloids and nitrogenous compounds, flavonoids, phenolic acids, coumarins, triterpenoids, and fatty acid derivatives.

Phenolic amides, represented primarily by *N*-feruloyl tyramine and related hydroxycinnamic acid amide derivatives, constituted a characteristic phytochemical class in *S. incanescens*, distinguishing it from the other investigated species [14]. Compound **2.1.7** (*m*/*z* 476.1903) was characterized as a probable identification for the phenolic amide glycoside, *trans*-*N*-feruloyl tyramine-4‴-*O*-β-D-glucopyranoside. The MS^2^ spectrum (positive mode) showed a diagnostic fragment at *m*/*z* 314.1351 (calculated *m*/*z* 314.1387), signifying the loss of the glucose moiety to produce the feruloyl tyramine aglycone (Figure S15). This compound was previously reported in *S. inermis*; however, this report represents its putative first detection in *S. incanescens* [14,49,50].

The precursor ion detected at *m*/*z* 506.2007 (R*_t_* 7.8222 min) with the molecular formula C_25_H_31_NO_10_ was identified as a glucoside derivative, cimicifugamide; *N*-(*trans*-4-*O*-β-D-glucopyranosyl feruloyl)-3′-*O*-methyldopamine (**2.1.8**; Figure S16) [51], previously isolated from the rhizomes of *Cimicifuga* species. This compound is reported here for the first time from *S. incanescens*.

In contrast, compound (**2.1.13**; Figure S17) [52] illustrates the fragmentation pattern of another precursor ion at *m*/*z* 344.1492 (R*_t_* 9.586783 min) with the molecular formula C_19_H_21_NO_6_, likely corresponding to the aglycone of the aforementioned compound. This structure has been previously identified in *S. incanescens* (syn. *Caroxylon volkensii*) as *N*-*trans*-feruloyl-3-*O*-methyldopamine or *N*-*trans*-feruloyl-4′-*O*-methyldopamine. However, the discrepancy in the methylation site on the dopamine moiety highlights the possibility of alternative structures, tentatively suggesting the presence of either 3-*O*-methyldopamine or 4-*O*-methyldopamine derivatives.

The MS^2^ spectra presented in Figures S18 and S19 revealed two distinct compounds with nearly identical molecular ions at *m*/*z* 360.1436 (**2.1.9**) and 360.1432 (**2.1.4**), respectively, suggesting they are structural isomers or analogs sharing the same molecular formula (C_19_H_21_NO_6_). Despite their similar mass, clear differences were observed in their retention times and structural features. Structurally, both compounds are based on a moupinamide scaffold. The MS^2^ spectrum in Figure S18 identified compound **2.1.9** as 2′-hydroxy-3″-methyoxymoupinamide, exhibiting a longer retention time (R*t* 8.244 min) [52]. In contrast, *N*-(3′,4′-dimethoxy-cinnamoyl)-norepinephrine (**2.1.4**; Figure S19) eluted earlier (R*_t_* 6.674 min), likely due to increased polarity from the catechol-derived norepinephrine core. These differences not only influenced their chromatographic behavior but also their fragmentation patterns, as exemplified by the fragment ion observed at *m*/*z* 167.0323 for compound **2.1.4** (Figure S19) [52].

Flavonoids represent the second most prominent class of phytochemicals detected in *S. incanescens*, comprising derivatives such as luteolin (**2.2.12**), kaempferol glycosides (**2.2.2**, **2.2.3**, and **2.2.10**), apigenin (**2.2.13**), diosmetin (**2.2.16**), isorhamnetin (**2.2.14**, **2.2.6**, and **2.2.8**), and (+)-catechin (**2.2.9**) [31,53] (Table S2).

A group of polyoxygenated triterpenoids was also detected, including salsolin A (**2.4.2**) and B (**2.4.4**) [14,54], salsoloside C (**2.4.3**) [52], and salsolic acid (**2.4.5**) [14,55], which were identified in negative ionization mode [14].

#### 3.1.3. Phytochemical Profiling by LC-MS/MS Analysis of *S. parviflora*

Erucamide (**3.7.2**, *m*/*z* 338.3417) was assigned a probable identification with a mass error of +2.66 ppm. The identification was supported by diagnostic fragments at *m*/*z* 303.303, 321.314, and 338.340, corresponding to the cleavage of the amide bond and the long-chain alkyl moiety (Figure S20) [31].

Another alkylamide (**3.7.1**) was probably identified as feruloyltyramine based on a protonated molecular ion [M + H]^+^ at *m*/*z* 314.1375, closely matching the theoretical mass of 314.1387 (mass error, −3.82 ppm). The assignment was further supported by a characteristic feruloyl cation base peak at *m*/*z* 177.055 and diagnostic fragment ions at *m*/*z* 145.028 and 121.065 (Figure S21) [31].

*S. parviflora* showed a rich profile of flavonoids and their glycosides with predominance of flavones, especially represented by luteolin derivatives, including *O*-(**3.1.2**, **3.1.6**, and **3.1.7**) and *C*-glycosides (**3.1.4** and **3.1.5**) and the aglycone, **3.1.10** [31,56,57,58,59]. Numerous amino acids (**3.3.1**–**3.3.13**) and several fatty acids or their derivatives (**3.5.1**–**3.5.5**) were among the identified structures (Table S3).

It is worth noting that the present study provides the first comprehensive phytochemical profile of *S. parviflora*. To date, only a limited number of phytochemical investigations have been conducted on this species, reporting the presence of two alkanes (eicosane and tetratetracontane), a fatty acid (palmitic acid), a phenolic acid (gallic acid), and the acyclic diterpene alcohol phytol [60,61,62].

### 3.2. Anti-Inflammatory Activities

Despite the availability of various therapeutic strategies, the treatment of ulcerative colitis (UC) and the preservation of colonic tissue integrity remain challenging. Current therapeutic agents include corticosteroids, sulfasalazine, 5-aminosalicylates, thiopurines, and, more recently, immunomodulators such as TNF-α and interleukin (IL) antagonists [7]. However, the side effects and toxicity associated with long-term use of these agents limit their clinical application, highlighting the need for safer and more effective alternatives. Traditional medicine continues to serve as an important source of alternative and adjunct therapies for many diseases. In the present study, we investigated the protective effects of three different plant extracts, *C. gileadensis*, *S. incanescens*, and *S. parviflora*, against acetic acid (AA)-induced UC in rats.

Determination of the DAI was performed as an initial assessment of the protective effects of the extracts against AA-induced UC. Macroscopic examination of colonic tissues revealed decreased tissue necrosis, inflammation, and ulceration. Decreased colonic weight-to-length ratio indicates decreased edema and supports the anti-inflammatory properties of the extracts; meanwhile, microscopic examination of the tissues using H&E stain supports the protective activity of the extracts with decreased ulceration, inflammation, and inflammatory cell infiltrates and increased mucosal integrity, evidenced by increased mucin expression seen with Alcin Blue stain. The highest protective activity against AA- induced UC was observed for *C. gileadensis* at a dose of 500 mg/kg.

UC is a recurrent inflammatory event in the colonic tissue that affects tissue integrity and the intestinal barrier, with a complex nature that allows several molecular signaling pathways to interplay. UC is characterized by the release of massive inflammatory cytokines and inflammatory cell infiltrates, accompanied by the release of reactive oxygen species, which leads to oxidative damage and worsens the condition. TLRs, and more specifically TLR4, are an integral part of the colonic innate immune system that is upregulated in UC. TLR4 allows the recognition of both DAMPs from damaged colonic tissues and PAMPs from colonic bacteria after epithelial disruption. Activation of TLR4 phosphorylates IRAK, which acts as a downstream target and amplification point within TLR-inflammatory signaling. In UC, p-IRAK exacerbates the transcription of inflammatory cytokines by targeting and activating NF-κB, a master regulator of inflammation. Activated NF-κB is translocated into the nucleus, inducing the expression of inflammatory cytokines such as TNF-α and IL-6. As observed in our study, treatment with AA increased the expression of TLR4, the phosphorylation of both IRAK and NF-κB, and the level of TNF-α and IL-6, confirming the role of the TLR4/IRAK/NF-κB pathway in UC pathogenesis. Our findings align with previous studies that document the role of TLR in colonic tissues and other tissues. Meanwhile, treatment with the extracts significantly impaired the TLR4 inflammatory pathway, showing decreased TLR4 expression, inhibited IRAK and NF-κB phosphorylation, and attenuated proinflammatory cytokine levels, with the highest protective activity observed for *C. gileadensis* at a dose of 500 mg/kg.

An additional downstream target of NF-κB is iNOS, which catalyzes the production of a powerful reactive nitrogen species (NO). Despite the physiological role of NO in vasodilatation, vascularization, and maintenance of mucosal defense, overproduction of NO under stressful inflammatory conditions causes oxidative stress, DNA damage, and tissue apoptosis. Our results demonstrated a significant increase in iNOS and NO levels in the UC model, indicating the dual action of NF-κB, which is the production of inflammatory cytokines and the induction of oxidative tissue damage.

The results obtained are consistent with previously reported activities for the plants, where several in vitro and in vivo studies on different extracts of *C. gileadensis* reported excellent antioxidant and anti-inflammatory, in addition to gastroprotective and anti-ulcer activities. The strong anti-inflammatory activities of the extracts, sometimes comparable to diclofenac in reducing edema and granuloma formation, were exerted via different mechanisms, including COX-1 inhibition, reduced proinflammatory mediators (PGE2, NO, TNF-α), inhibition of protein denaturation, and NF-κB suppression. Extracts also exhibited significant protection against oxidative stress by showing strong antioxidant activity either in vitro, sometimes results close to ascorbic acid, or in vivo by enhancing antioxidant biomarkers (SOD, CAT, GST, GSH) and reducing lipid peroxidation [30]. Additionally, the ethanol extract of *C. gileadensis* showed a dose-dependent ulcer-protective effect by preserving gastric mucus and nonprotein sulfhydryl NP-SH levels [28].

Genus *Salsola* has been extensively documented for its antioxidant and anti-inflammatory properties, especially due to its content of phenolic compounds, especially flavonoids and flavonoid glucosides from aqueous and hydroalcoholic extracts [14,63]. In addition, Elwekeel et al. [64] reported antioxidant and anti-inflammatory activities of the non-polar extract of five *Salsola* species, attributing these effects, at least in part, to their high contents of fatty acids and fatty acid methyl esters. Among the investigated species, *S. villosa* exhibited the strongest COX-2 inhibitory activity, whereas *S. imbricata* showed the most potent inhibition of COX-1 [64].

In the same context, *S. parviflora* extracts have exhibited antioxidant and anti-inflammatory activities attributed to polyphenol constituents [65]. A herbal mixture containing the plant showed good anti-ulcerogenic activities [62].

The LC-MS/MS analysis of *C. gileadensis*, *S. incanescens*, and *S. parviflora* showed that the three plants contain a rich profile of flavonoids and flavonoid glycosides as well as unique compounds characteristic of each plant (Table 1 and Tables S1–S3). In addition to the flavonoids, *C. gileadensis* is characterized by triterpenes (oleanane and ursane types) and phenolic acids, while *S. incanescens* is characterized by the distinct presence of phenolic amides and polar triterpenoid derivatives (oleanane/ursane types), and finally, *S. parviflora* is characterized by Brassicaceae-type amino acids and lipids.

Several detected classes are well-recognized for their pharmacological potential, particularly in stress response and anti-inflammatory pathways of the three plants. Flavonoids are likely key contributors to the observed anti-inflammatory potential. They are renowned for their multi-target anti-inflammatory mechanisms, such as inhibiting key pro-inflammatory enzymes such as COX-2 and inducible iNOS, thereby reducing the synthesis of inflammatory mediators, such as prostaglandins and nitric oxide [66,67,68,69]. They also modulate the expression and activity of pro-inflammatory cytokines, including tumor necrosis factor-alpha (TNF-α), interleukin-1β (IL-1β), and interleukin-6 (IL-6), which are crucial to the inflammatory response [66]. A key mechanism involves the suppression of transcription factors such as nuclear factor kappa B (NF-κB) and activator protein-1 (AP-1), leading to downregulation of genes involved in inflammation and immune cell recruitment [66,69,70]. Additionally, flavonoids can inhibit the secretion of arachidonic acid and related enzymes, further reducing inflammatory cascades [66,67]. Their antioxidant properties also contribute to their anti-inflammatory action by neutralizing reactive oxygen species (ROS) and reducing oxidative stress, which is closely linked to chronic inflammation [71]. Among these, methoxylated flavonols, particularly isorhamnetin and its glycosides (e.g., isorhamnetin-3-*O*-glucoside and isorhamnetin-3-*O*-rutinoside), merit special attention because of their well-documented anti-inflammatory and cytoprotective properties [69,72,73]. These compounds were identified in all three species (Table 1), with a greater diversity in *C. gileadensis*, and some were among the metabolites selected for molecular docking. Structurally, methylation of the flavonol scaffold increases lipophilicity and metabolic stability relative to non-methylated analogues, whereas glycosylation enhances aqueous solubility but generally reduces passive intestinal absorption. Following oral administration, isorhamnetin glycosides function as prodrugs that are hydrolyzed by intestinal enzymes and gut microbiota to release the more readily absorbed aglycone, isorhamnetin, thereby enhancing its biological availability and pharmacological activity. Once absorbed, isorhamnetin has been reported to attenuate oxidative stress and inflammatory responses through modulation of the NF-κB, MAPK, and Nrf2 signaling pathways, leading to reduced production of pro-inflammatory mediators and enhanced cellular antioxidant defenses [73]. These pharmacokinetic and pharmacodynamic characteristics provide a plausible explanation for the favorable docking behavior of isorhamnetin derivatives and support their potential contribution, together with other constituents, to the anti-ulcerative colitis activity observed for the crude extracts. Nevertheless, because the biological evaluation was performed using unfractionated extracts, the contribution of individual isorhamnetin derivatives remains putative and requires confirmation through bioactivity-guided isolation and targeted pharmacological studies.

Triterpenoid derivatives are also well recognized for their anti-inflammatory properties, primarily through the modulation of key inflammatory signaling pathways. Numerous triterpenes, including oleanolic acid, asiatic acid, maslinic acid, and various lanostane derivatives, have been shown to suppress the nuclear translocation of NF-κB, a central regulator of inflammation, thereby reducing the expression of pro-inflammatory mediators, such as NO, iNOS, IL-6, and TNF-α [74,75,76]. In addition, triterpenes can inhibit the phosphorylation of MAPK proteins (ERK1/2, p38, JNK1/2), further inhibiting inflammatory responses [74,76]. Their antioxidant activity, attributed in part to the presence of hydroxyl and carboxyl functional groups, may also contribute to the mitigation of inflammation-associated oxidative stress [74,75,76]. Notably, triterpenoids and their derivatives were identified by the current study in *C. gileadensis* and *S. incanescens* (Table 1) and may therefore contribute to the observed anti-inflammatory effects of these extracts.

A prominent group detected includes *N*-feruloyltyramine derivatives, such as *trans*-*N*-feruloyltyramine-4‴-*O*-β-D-glucopyranoside and *N*-*trans*-feruloyl-3-*O*-methyldopamine. These compounds are phenolic amides (or hydroxycinnamic acid amides), with established roles in modulating inflammatory responses. Studies reported that feruloyltyramines inhibit NO production in LPS-stimulated macrophages and downregulate COX-2 expression, both of which are critical steps in the inflammatory cascade. Feruloyltyramines, such as *N*-*trans*-feruloyltyramine, have been shown to exert anti-inflammatory effects by strongly suppressing the mRNA expression of iNOS and COX-2, resulting in reduced production of NO and PGE2 in LPS-stimulated macrophages. This inhibition is mediated through the suppression of the AP-1 transcription factor and decreased expression and phosphorylation of the c-Jun N-terminal kinase (JNK) protein, indicating that *N*-*trans*-feruloyltyramine acts *via* the AP-1 and JNK signaling pathways to downregulate these key inflammatory mediators [77]. Additionally, the relationship between NO and COX-2 is complex; for example, in rat peritoneal macrophages, NO negatively regulates COX-2 expression, as inhibition of NO production leads to enhanced COX-2 protein levels and activity [78]. This dynamic regulation underscores the importance of targeting both NO and COX-2 pathways in the control of inflammation. Also, feruloyltyramine (**3.7.1**), a phenolic amide derived from the conjugation of ferulic acid and tyramine, is well recognized for its antioxidant and anti-inflammatory activities and is often reported in stress-induced plant secondary metabolism [77,79]. The identification of feruloyltyramines in *S. incanescens* and *S. parviflora* (Table 1), particularly their abundance in the former species, suggested that these metabolites may contribute to the observed biological activities of this extract.

Both *S. incanescens* and *S. parviflora* extracts indicated the potential occurrence of erucamide (**2.1.17** and **3.7.2**), a naturally occurring primary fatty acid amide known to possess anti-inflammatory and antimicrobial properties, which may contribute to the biological properties of these extracts [80,81,82] (Table 1 and Tables S2 and S3). However, despite the presence of this bioactive metabolite, *S. parviflora* exhibited the weakest biological activity among the investigated extracts, suggesting that the relatively low abundance of erucamide and/or the total phytochemical composition was insufficient to produce pronounced anti-inflammatory effects.

Natural polyphenols and flavonoids are widely recognized for their therapeutic benefits, particularly their capacity to mitigate oxidative stress, scavenge free radicals [83], and downregulate pro-inflammatory cascades by blocking key enzymatic pathways such as cyclooxygenase (COX) [84]. However, the present results demonstrated that total phenolic (TPC) and flavonoid (TFC) contents do not necessarily correlate with biological efficacy. Although *S. parviflora* showed the highest TPC (26.29 ± 2.50 µg GAE/mg) and a relatively high TFC, it exhibited the weakest anti-inflammatory and anti-UC activities. In contrast, *C. gileadensis*, which exhibited the highest TFC (13.67 ± 1.00 µg QE/mg) but lower TPC, demonstrated the greatest biological efficacy, which may be attributed to its flavonoid- and triterpenoid-rich phytochemical composition. Similarly, *S. incanescens* displayed moderate anti-inflammatory activity despite exhibiting the lowest TPC and TFC, suggesting that its efficacy may be associated with other bioactive constituents, particularly phenolic amides, in addition to phenolics and flavonoids. These findings are further supported by the LC-MS/MS profiling and molecular docking analyses, which identified flavonoids, triterpenoids, and phenolic amides with favorable interactions toward TLR4/MD-2 and COX-2. Thus, the present findings suggest that qualitative chemodiversity is a more reliable predictor of anti-inflammatory efficacy than conventional TPC and TFC assessments. Future bioassay-guided isolation and characterization studies are warranted to identify the specific compounds responsible for the observed anti-inflammatory activity.

### 3.3. Docking Studies

The in vivo studies showed that *C. gileadensis* exhibits the highest anti-inflammatory activity, followed by *S. incanescens.* These results are further supported by potential affinities of their compounds toward TLR4 and COXs (COX-1/COX-2) molecular targets.

The primary function of the TLR4 receptor is to detect lipopolysaccharide (LPS) from Gram-negative bacteria, a process that initiates the innate immune response and triggers receptor activation upon association with the MD-2 protein. Once stimulated, TLR4 dimerizes and initiates two distinct signaling cascades to ramp up inflammation [85]. Antagonists compete for the same binding pocket as LPS but do not induce the conformational change required for dimerization and signaling; this effectively prevents the downstream phosphorylation of p65 (NF-κB) and the activation of Caspase-1, thereby reducing inflammatory markers and pyroptosis. This dimerization is triggered only when a ligand (like LPS) is partially exposed to the pocket’s entrance, creating a “bridge” to the second TLR4 molecule [86]. Antagonists like Eritoran (PDB ID: E55), in the crystal structure of the human TLR4/MD-2 complex (PDB ID: 2Z65), prevent this by being fully sequestered deep within the large hydrophobic pocket of the MD-2 co-receptor, leaving no part exposed to form a bridge [87]. The interaction is characterized by extensive hydrophobic contacts with the acyl chains, specific loop lid, and specific polar/ionic interactions with the phosphate and sugar moieties. Structural stability is maintained by four acyl chains that fill the internal MD-2 cavity. This anchor is driven by an aromatic “phenylalanine-rich” region (Phe76, Phe119, Phe121, Phe151) that packs against lipid tails, combined with specific Isoleucine (Ile32, Ile46, Ile52, Ile80, and Ile117), Valine (Val24, Val48, Val82, Val135), and Leucine (Leu54, Leu61, Leu71) residues. A specific loop in MD-2 (residues 82–87) closes over the ligand. The orientation of Phe126 is particularly important. In the antagonist-bound state, it is oriented inward to help sequester the chains, whereas in the agonist-bound state (PDB ID: 2Z65), it is oriented inward, helping to sequester the lipid chains. In the agonist-bound state (PDB ID: 3FXI), it flips outward to facilitate dimerization with the second TLR4 molecule [85]. The polar head of Eritoran, which includes the glucosamine rings and the phosphate groups, forms an ionic/polar lock that prevents the pathogenic ligand from initiating inflammation. Ser118, Ser120, and Tyr102 residues can form hydrogen bonds with sugar moieties or phosphate oxygens [85].

**Figure 10:**
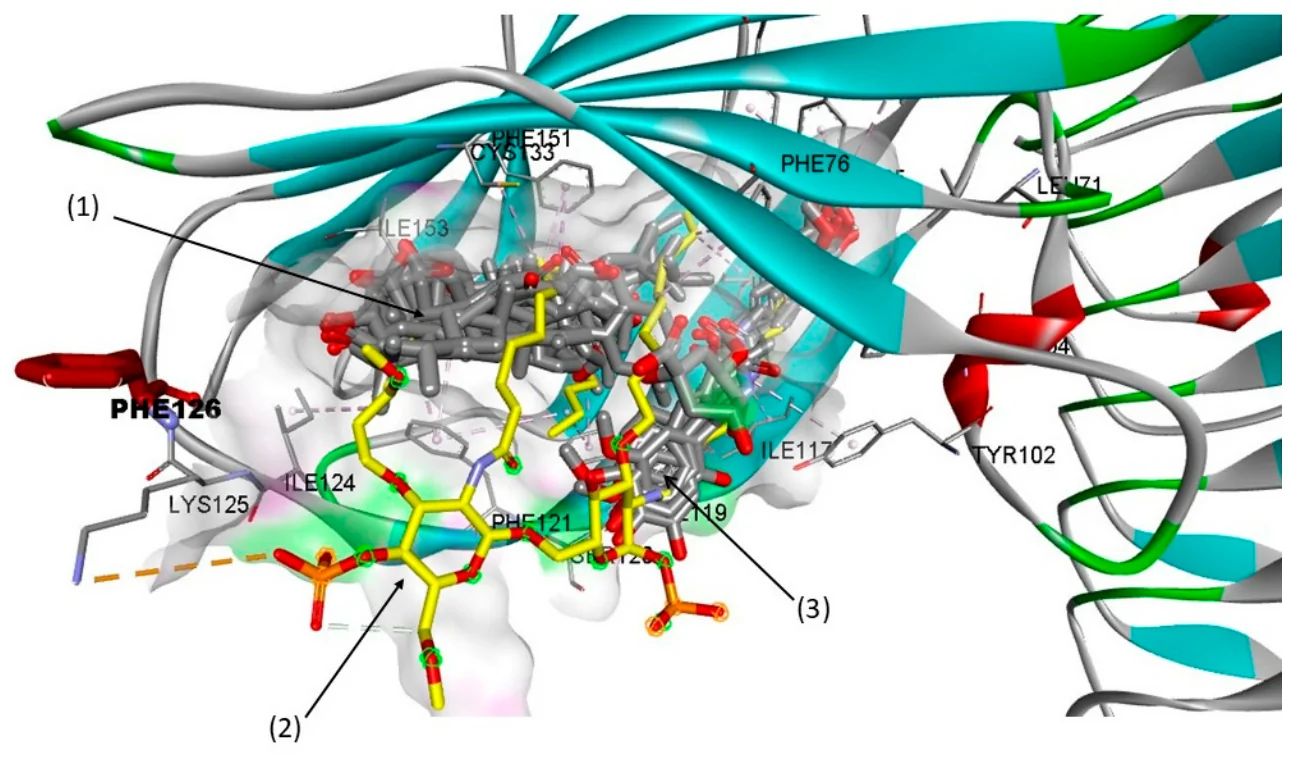
Comparative 3D molecular docking visualization within the TLR4/MD-2 complex (PDB ID: 2Z65). (1) Pentacyclic triterpenoids and phytosteroidal scaffolds (oleanonic aldehyde, 11-oxo-oleanonic acid, 11-oxo-ursonic acid, oleanonic acid, commigileadin A, urosonic acid, canophyllal, urosonic aldehyde, salsolin B, amasterol, and stigmasterol) exhibiting deep hydrophobic sequestration. (2) Protocol validation through redocking of the co-crystallized antagonist Eritoran (E55). (3) Phenolic amides and moupinamide derivatives (*N*-feruloyltyramine, *N*-caffeoyltyramine, (*E*)-3-(4-hydroxy-3-methoxyphenyl)-*N*-(4-methoxyphenethyl) acrylamide, 2-hydroxy-3″-methyoxymoupinamide, *N*-*trans*-feruloyl-3-*O*-methyldopamine, and *N*-(3,4′-dimethoxy-cinnamoyl)-norepinephrine) alongside chlorogenic acid, illustrating their flexible interactions within the polar headgroup region of the MD-2 pocket.

The docking results provide a theoretical molecular framework that serves as hypothesis-generating support for the observed in vivo anti-inflammatory activity. These compounds can be classified into two major phytochemical classes based on their interaction profiles within the TLR4/MD-2 binding site. The first class, predominant in *C. gileadensis*, comprised lipophilic, rigid pentacyclic triterpenoids and phytosterols, such as commigileadin A and stigmasterol. These compounds are predicted to orient within the large hydrophobic pocket of the MD-2 co-receptor, maximizing van der Waals interactions with critical hydrophobic clusters, including phenylalanine (Phe76, Phe119, Phe121, Phe151), isoleucine, and leucine residues. It was suggested that their large molecular volumes promote deeper sequestration within the hydrophobic cavity of MD-2, compared with smaller and more slender molecules, such as moupinamides, flavones, and coumarins (Figure 10), though experimental confirmation remains necessary. Interestingly, the polar functional groups of salsolic acid and salsolin A may lead to binding patterns that deviate from purely hydrophobic sequestration, potentially explaining the moderate activity of *S. incanescens*. The second class, primarily associated with *S. incanescens*, includes phenolic amides (moupinamide derivatives like *N*-*trans*-feruloyl-3-*O*-methyldopamine) and caffeoylquinic acids (e.g., chlorogenic acid). These molecules consist of aromatic rings connected by flexible chains. While they are highly effective at forming multiple hydrogen bonds in the polar region of the MD-2 binding pocket due to the presence of multiple hydroxyl (-OH) and methoxy (-OCH_3_) groups, they lack the rigid, bulky hydrophobic core of triterpenes. Although these amides showed favorable predicted individual binding affinities (e.g., *N*-*trans*-feruloyl-3-*O*-methyldopamine with an S-score of −6.64 kcal/mol for TLR4), their flexible nature is predicted to make them less capable of deep sequestration than the bulkier, rigid triterpenoids.

Cyclooxygenases (COX) are key enzymes in the biosynthesis of prostaglandins, which play crucial roles in various physiological and pathological processes, including inflammation. Two main isoforms, COX-1 and COX-2, exist with distinct physiological functions. Selective inhibition of COX-2 over COX-1 is a therapeutic strategy to reduce inflammatory side effects while minimizing adverse effects on normal physiological functions [88].

**Figure 11:**
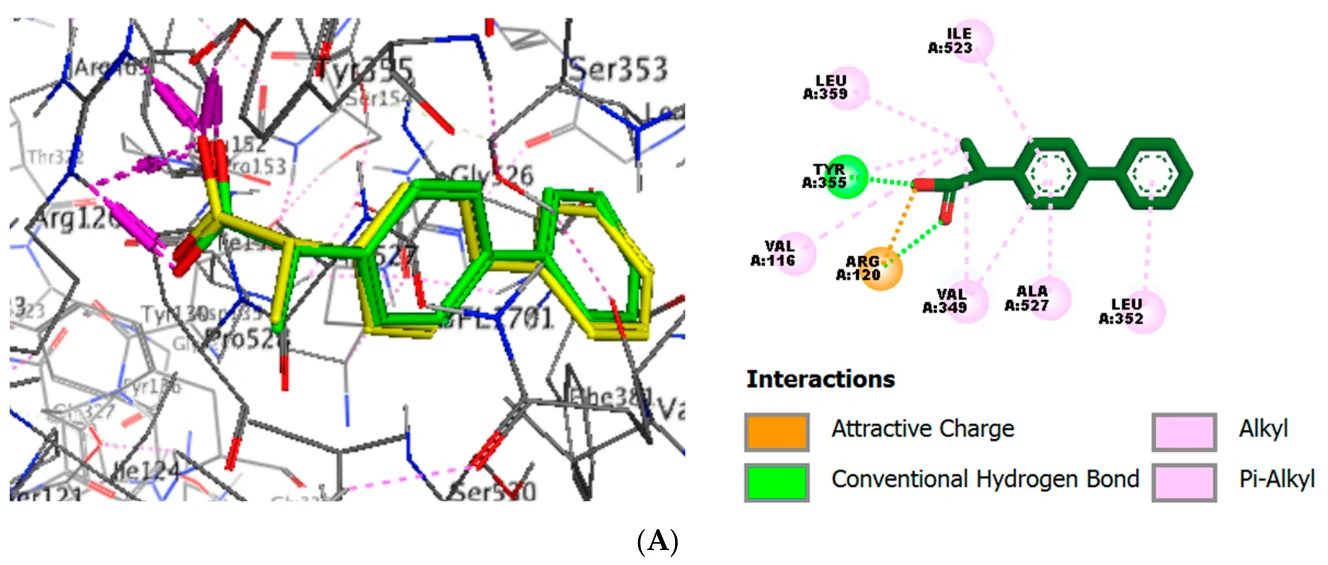

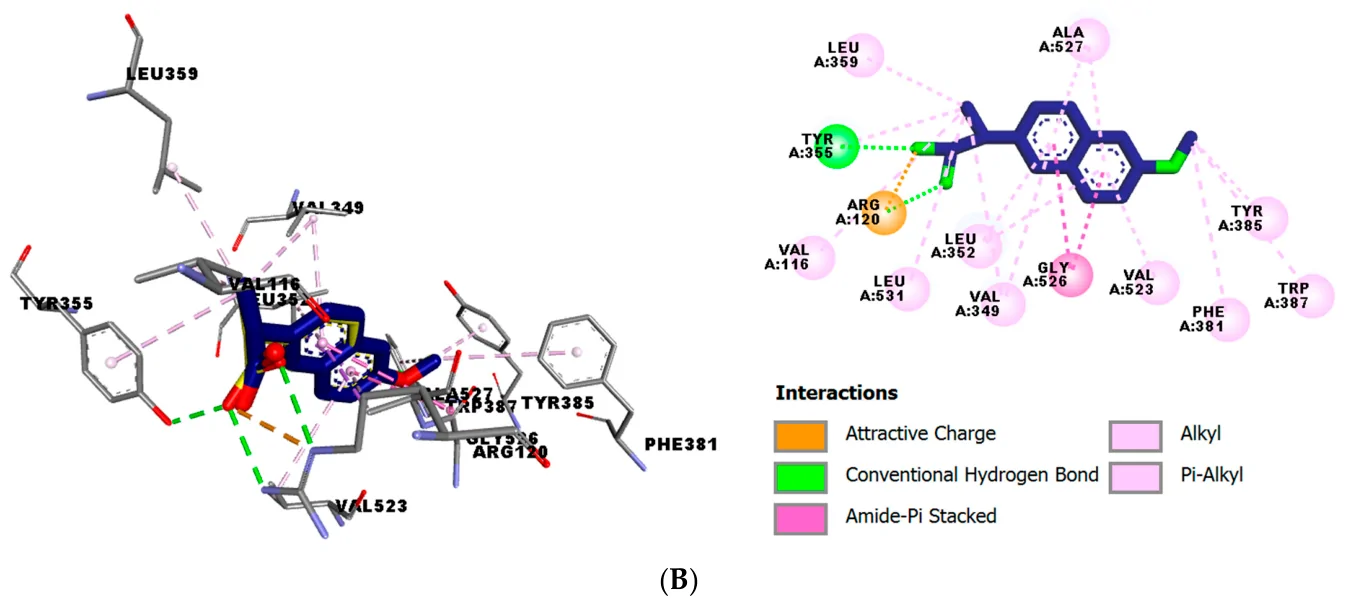
(**A**) 3D and (**B**) 2D validation of the docking protocol by redocking the crystallized ligand BFL and NPS into the COX-1 and COX-2 (PDB ID: 1Q4G and 3NT1), respectively.

The redocking validation using the crystallized ligands fluprofen and naproxen (PDB ligands: BFL and NPS) into COX-1 (PDB ID: 1Q4G) and COX-2 (PDB ID: 3NT1) active sites, respectively [88,89], confirming accurate placement of the ligand within the orthosteric site and validating the chosen docking parameters. The redocked pose maintained the expected network of interactions observed in the crystal structure, including the bidentate H-bond that defines the canonical anchoring region for acidic NSAIDs Arg120, Tyr355 at the mouth of the channel for both ligands while the naphthyl scaffold extends deep into the hydrophobic cavity and engages in van der Waals contacts with residues, such as Val349, Leu523 and Leu352 in case fluprofen; while in naproxen binds with residues such as Leu352, Val349, Gly526, Val523, Phe381, Tyr385, Trp387 and Ala627 supporting the protocol’s ability to recover experimentally observed binding modes (Figure 11).

Upon analyzing the data provided for the binding affinity of the docked phytocompounds into COX active sites (Figures S25 and S26), several compounds exhibited differential binding preferences (Table 6). Compounds such as *N*-*trans*-feruloyl-3-*O*-methyldopamine (−7.09), (*E*)-3-(4-hydroxy-3-methoxyphenyl)-*N*-(4-methoxyphenethyl) acrylamide (−6.69), and *N*-caffeoyltyramine (−6.48) showed the most favorable S-scores for COX-2, suggesting a higher binding affinity and potential selectivity towards this isoform. These compounds also maintained acceptable RMSD values, indicating stable binding poses. Conversely, kaempferide (−5.79), syringetin (−5.60), and scopoletin (coumarin) (−5.85) exhibited more favorable S-score values for COX-1, suggesting a preference for this enzyme. The observed binding preferences can be correlated with the distinct pharmacophore requirements of COX-1 and COX-2. Ligands that showed higher affinity for COX-2 likely possess structural features that enable them to exploit the larger active site and the unique side pocket. Especially, compounds with bulkier substituents or specific hydrophobic/aromatic moieties might fit well into the COX-2 side pocket, forming interactions with residues like Val523, Arg513, Gln192, His90, Ser353, or Leu252. The presence of hydrogen bond donors/acceptors in these ligands could facilitate interactions with polar residues within the side pocket or the main channel, such as Gln192 or Ser353. For compounds exhibiting COX-1 preference, it is plausible that their structures are better suited for the narrower active site of COX-1, avoiding steric clashes with Ile523. These ligands might primarily engage in interactions within the main hydrophobic channel and form hydrogen bonds with conserved residues like Arg120 and Tyr355 at the entrance, or Ser530 in the central pocket, without extending into the blocked side pocket region. Further detailed analysis of the individual docking poses for each ligand, including specific hydrogen bonding, hydrophobic interactions, and pi-stacking with the identified key residues (Arg120, Tyr355, Ser530, Val523/Ile523, Arg513/His513, Gln192, etc.), would provide a more precise understanding of the pharmacophore requirements for COX-1 and COX-2 selectivity among these plant-derived compounds.

Though both plants exhibited significant anti-inflammatory properties, the superior activity of *C. gileadensis* may be attributed to its broad phytochemical diversity, including flavonols such as quercetin, myricetin, and taxifolin that provided potential inhibition of both COXs, alongside coumarins like scopoletin and daphnetin, which showed a specific preference for COX-1. In contrast, *S. incanescens* displayed moderate activity characterized by highly potent but more targeted phenolic amides that dominate COX-2 docking results; notably, *N*-*trans*-feruloyl-3-*O*-methyldopamine showed the strongest affinity with an S-score of −7.09, followed by (*E*)-3-(4-hydroxy-3-methoxyphenyl)-*N*-(4-methoxyphenethyl) acrylamide (−6.69) and *N*-caffeoyltyramine (−6.48).

Thus, the superior pharmacological profile of *C. gileadensis* may be attributed to its diverse phytochemical composition, which includes unique triterpenoid and flavonoid derivatives capable of targeting multiple inflammatory pathways, such as COX-1, COX-2, and TLR4/MD-2 complex. This broader mechanism of action may underlie its enhanced anti-inflammatory activity compared with the phenolic amides of *S. incanescens*, which are thought to exhibit a more selective affinity for COX-2.

To summarize these findings, Table 7 provides a comparative overview of the phytochemical, biological, and computational results for the three investigated Arabian Desert plants. It highlights the potential multi-target anti-ulcerative colitis activity of *C. gileadensis* and indicates the moderate and limited anti-ulcerative colitis activities of *S. incanescens* and *S. parviflora*, respectively.

## 4. Materials and Methods

### 4.1. Plant Material

Aerial parts of *C. gileadensis*, *S. incanescens*, *and S. parviflora* were collected from regions in Makkah, Arar, and Hafar Al-Batin, Kingdom of Saudi Arabia, in December 2023, November 2023, and January 2024, respectively, without harming the environment during the collection of the plants and with approval given by the Ministry of Environment, KSA, for the collection of aerial parts for research purposes. The plant was defined by Hamdan Al-Hassan’s book (Wild Plants of the KSA) and the Camel and Range Research Center, Al-Jouf. Voucher specimens of the plants (5*Cgi*1/2026, 42*Sin*1/2026, and 4*Spa*2/2026, respectively) were preserved at the Pharmacognosy Department’s herbarium at the Faculty of Pharmacy, Capital University (formerly, Helwan University), Egypt.

### 4.2. Preparation of the Extracts

Air-dried aerial parts of *C. gileadensis* (598.585 g), *S. incanescens* (416.285 g), and *S. parviflora* (514.566 g) were exhaustively extracted by maceration with 98% methanol (Sigma-Aldrich, USA; 5 × 1 L for each sample) until complete extraction was achieved. The combined extracts were concentrated under reduced pressure using a rotary evaporator (R-215, BUCHI, Flawil, Switzerland) equipped with a V-850 vacuum controller at 48 °C to obtain the 98% aerial parts methanol extracts (AMEs). The dried AMEs yielded 71.280 g, 36.612 g, and 111.267 g, corresponding to extraction yields of 11.9%, 8.8%, and 21.6% (*w*/*w*) for *C. gileadensis*, *S. incanescens*, and *S. parviflora*, respectively.

### 4.3. LC-MS/MS Analysis

Secondary metabolites in the investigated extracts were tentatively characterized by LC–MS/MS. Samples (50 mg) were dissolved in water:methanol:acetonitrile (50:25:25, *v*/*v*/*v*), vortexed, ultrasonicated, centrifuged, and diluted to a final concentration of 2.5 μg/μL. Aliquots (10 μL) were analyzed in both positive and negative ionization modes using a Sciex ExionLC–TripleTOF 5600+ UPLC–QTOF–MS/MS system (SCIEX, Marlborough, MA, USA) equipped with a Waters XSelect HSS T3 column (2.1 × 150 mm, 2.5 μm). Chromatographic separation was achieved using a gradient of ammonium formate buffer (pH 3 for positive mode and pH 8 for negative mode) and acetonitrile at a flow rate of 0.3 mL/min and a column temperature of 40 °C. Raw data were processed using MS-DIAL 4.9, while peak detection and extraction were performed using PeakView^®^ 2.2 and MasterView™ software (SCIEX, USA). Metabolite annotations were categorized according to conceptual identification levels (Probable, Tentative, Putative) Schymanski et al. [27]. Due to the lack of reference standard comparisons, no Confirmed (Level 1) identifications were reported. Compounds exceeding a mass error of ±10 ppm were downgraded to the tentative or putative levels. Annotations were filtered using strict criteria, including signal-to-noise ratio > 10, mass error ≤ ±10 ppm, and library matching score ≥ 70%.

### 4.4. Determination of Total Phenolic Content (TPC)

The total phenolic content was determined using the Folin–Ciocalteu microplate assay described by Attard [90]. Dry extracts were dissolved in HPLC-grade methanol (10 mg/mL). Gallic acid (31.25–500 μg/mL) was used to construct the calibration curve. Briefly, 10 μL of sample or standard was mixed with 100 μL of 1:10 diluted 2 M Folin–Ciocalteu reagent and 80 μL of 1 M sodium carbonate in a 96-well plate. After incubation for 20 min at room temperature in the dark, absorbance was measured at 630 nm using a FLUOstar Omega microplate reader (BMG LABTECH, Ortenberg, Germany). Results were calculated from the gallic acid calibration curve after blank correction and expressed as μg gallic acid equivalents (GAE)/mg dry extract. All measurements were performed in triplicate.

### 4.5. Determination of Total Flavonoid Content (TFC)

Total flavonoid content (TFC) was determined using a modified aluminum chloride colorimetric microplate assay according to Kiranmai et al. [91]. Quercetin (31.25–500 μg/mL) served as the calibration standard. Aliquots of 15 μL of extract (10 mg/mL) or standard were mixed with 175 μL methanol, 30 μL of 1.25% AlCl_3_, and 30 μL of 0.125 M sodium acetate. After incubation for 5 min at room temperature, absorbance was measured at 420 nm. Sample color blanks were subtracted before calculation. TFC was expressed as μg quercetin equivalents (QE)/mg dry extract. Analyses were performed in triplicate.

### 4.6. In Vitro Assay

#### 4.6.1. Quantification of IL-6 and TNF-α by ELISA

RAW264.7 macrophages were seeded at 1–2.5 × 10^5^ cells/well in 24-well plates and cultured overnight. Cells were then pretreated with tested extracts or celecoxib at concentrations ranging from 1 to 100 µM for 1 h. The final DMSO concentration in all treatment groups did not exceed 0.1% (*v*/*v*). Subsequently, cells were stimulated with 1 µg/mL lipopolysaccharide (LPS) for 24 h. Negative control cells received neither LPS nor test compounds. Positive control cells received LPS only. Cell culture supernatants were then collected, centrifuged at 1000× *g* for 10 min at 4 °C, and stored at −80 °C until analysis. The concentrations of interleukin-6 (IL-6) and tumor necrosis factor-alpha (TNF-α) were measured using Abcam ELISA kits (ab178013, ab208348, respectively, Cambridge, UK), according to the manufacturers’ instructions.

Before analysis, all reagents and samples were allowed to equilibrate to room temperature (18–25 °C). Standard curves were generated for each assay by reconstituting the lyophilized cytokine standards in the supplied sample diluent, followed by serial dilution. Aliquots (50 μL) of standards, controls, or samples were transferred to the appropriate wells of anti-tag-coated microplates, after which 50 μL of the corresponding antibody cocktail was added. The plates were sealed and incubated for 1 h at room temperature with continuous shaking at 400 rpm. Following incubation, the wells were washed three times with 350 μL of 1× Wash Buffer PT. Subsequently, 100 μL of TMB substrate solution was added to each well, and the plates were incubated for 10 min in the dark while shaking at 400 rpm. The enzymatic reaction was terminated by the addition of 100 μL of stop solution, and absorbance was measured at 450 nm using a BioLine ELISA microplate reader. All samples were analyzed in duplicate, and three independent experiments were performed (*n* = 3). Cytokine concentrations were calculated from the corresponding standard curves and expressed as pg/mL (mean ± SEM).

#### 4.6.2. In Vitro Anti-Inflammatory Activities Against COX-1 and COX-2 Enzymes

The in vitro inhibition of COX-1/COX-2 was assessed using BioVision Fluorometric kits (Cat no: K548-100 and K547-100, respectively, Milpitas, CA, USA). The IC_50_ values of the tested compounds were calculated. In addition, the COX-2 selectivity index (S.I) was evaluated and compared with that of celecoxib, a known selective COX-2 inhibitor.

In brief, all reagents were prepared according to the manufacturers’ instructions. Test extracts were dissolved in DMSO and diluted to prepare concentrations ranging from 0.01 to 100 µM with COX Assay Buffer. For each assay, 10 µL of diluted test compound (or assay buffer for enzyme control) was added to the determined wells of a 96-well white opaque plate. For the inhibitor control, 2 µL of the provided control inhibitor (SC560 for COX-1; celecoxib for COX-2) was mixed with 8 µL of COX Assay Buffer.

Arachidonic acid was reconstituted in 100% ethanol, then diluted in NaOH and deionized water according to the kit protocols. The reaction was initiated by adding 10 µL of the diluted arachidonic acid solution to each well. Fluorescence was measured kinetically at 25 °C using a Tecan Spark plate reader with excitation at 535 nm and emission at 587 nm for 10 min.

The slope (change in relative fluorescence units per minute, ΔRFU/min) was calculated for each well using two time points (T_1_ and T_2_) within the linear range of the reaction. Percent inhibition was calculated relative to the enzyme control (no inhibitor). IC_50_ values were determined by non-linear regression analysis of inhibition curves and are expressed as µM (mean ± SD). Selectivity indices (SI) were calculated as IC_50_ (COX-1)/IC_50_ (COX-2). All measurements were performed in duplicate (*n* = 3 independent experiments).

#### 4.6.3. Nitric Oxide Scavenging Capacity Using Sodium Nitroprusside–Griess Assay

The nitric oxide (NO) radical scavenging activity of the AMEs was evaluated using the Griess reagent method, with ascorbic acid as a positive control.

Test samples were initially dissolved in DMSO and subsequently diluted with phosphate-buffered saline (PBS, pH 7.3) to obtain concentrations ranging from 10 to 200 μg/mL. The final concentration of DMSO in all assay mixtures was maintained below 0.1% (*v*/*v*). Nitric oxide scavenging activity was evaluated using sodium nitroprusside (SNP) as a nitric oxide donor. The reaction mixture contained 5 mM SNP in PBS, either alone (control) or in the presence of various concentrations of the test samples. The mixtures were incubated at 25 °C for 180 min under visible polychromatic light generated by a 25 W tungsten lamp to facilitate nitric oxide release. The liberated nitric oxide reacted with atmospheric oxygen to form nitrite ions. At 30 min intervals, 1.0 mL aliquots of the incubation mixture were combined with an equal volume of Griess reagent, consisting of 1% sulfanilamide in 5% phosphoric acid and 0.1% *N*-(1-naphthyl)ethylenediamine dihydrochloride. The resulting chromophore was quantified by measuring absorbance at 540 nm using a BioLine ELISA microplate reader. Each concentration was tested in triplicate, and the experiment was repeated independently three times (*n* = 3). Nitric oxide scavenging activity was expressed as percentage inhibition relative to the control group containing SNP without the test sample. The IC_50_ value, defined as the concentration required to inhibit 50% of nitric oxide radicals, was calculated and expressed as μg/mL.

### 4.7. In Vivo Assay

#### 4.7.1. Animals

Female healthy adult *Sprague Dawley* rats, weighing 150–180 g, were used in the present study. The rats were left for 1 week of acclimatization to the standard laboratory conditions at a temperature (23 °C ± 2 °C), humidity (55%), 12 h light-dark cycle, and free access to a pellet diet and water *ad libitum*. They were obtained from the breeding unit of the Egyptian Organization of Biological Products and Vaccines (Helwan, Cairo, Egypt). Animals were included only if they exhibited normal behavior, intact skin prior to treatment, and no signs of disease or injury. The experiment was held in the animal house at the Faculty of Pharmacy, Capital University. They were handled according to the guidelines for animal care approved by the Institutional Animal Care and Use Committee (IACUC), Capital University (formerly, Helwan University), Egypt (Protocol No.: 26 A2024).

Animals were excluded from analysis if they met predefined exclusion criteria established prior to the experiment, in accordance with ARRIVE guidelines. These included poor general health prior to induction, failure to develop acetic acid–induced colitis, or technical complications during induction (e.g., rectal perforation, leakage, or excessive bleeding). Animals that died during the early post-induction period were also excluded. Any deviations from the approved experimental protocol, including incorrect dosing or improper sample collection, result in exclusion from final analysis.

#### 4.7.2. Drugs and Chemicals

Acetic acid (AA) was obtained from CID Pharmaceutical Company (Giza, Egypt) and Sulfasalazine from Minapharm Company (Giza, Egypt).

#### 4.7.3. Acute Toxicity Study

The acute toxicity study for the AME of *C. gileadensis*, *S. incanescens*, and *S. parviflora* was conducted on healthy adult Swiss albino mice of both sexes, weighing 25–30 g. Mice were treated with different doses of the AME up to 5 g/kg based on OECD Guideline 423, and then were observed for any behavioral or motor changes and mortality for 24 h.

#### 4.7.4. Ulcerative Colitis (UC) Model Induction in Rats

The model of UC was established in rats by intrarectal (IR) administration of AA according to a previously published study by Ghasemi-Dehnoo et al. [5]. Rats were lightly anesthetized with ether, following a 24 h fast. A single dose (2 mL) of 4% AA was administered intrarectally (IR) to reach the colon using a 2 mm soft pediatric catheter lubricated with Vaseline. After the slow administration of AA, rats were maintained in a head-down position for 60 s to prevent the leakage of AA. Similarly, 2 mL of normal saline was administered to the control rats.

##### Experimental Design

Fifty-four rats were randomly divided into nine experimental groups (*n* = 6):

- Group I: Control group received normal saline (2 mL/kg/PO/14 days)
- Group II: UC group received normal saline (2 mL/kg/PO/14 days).
- Group III and IV: *C. gileadensis* AME groups, received 250 mg/kg/PO and 500 mg/kg/PO of the AME, respectively) for 14 days.
- Group V and VI: *S. incanescens* AME groups, received 250 mg/kg/PO and 500 mg/kg/PO of the AME, respectively) for 14 days.
- Group VII and VIII: *S. parviflora* AME groups, received 250 mg/kg/PO and 500 mg/kg/PO of the AME, respectively) for 14 days.
- Group IX: Standard group received sulfasalazine (500 mg/kg/PO) for 14 days [92].

Random allocation was performed by sequentially drawing individual rats from the holding cage and assigning them to one of the nine treatment groups using a lottery method (i.e., each rat was placed into a cage chosen at random without replacement until all groups reached *n* = 6).

Rats were orally pretreated once daily with the assigned extract or dexamethasone for 14 consecutive days before induction of ulcerative colitis. At the end of the experiment, the rats were fasted for 24 h, then a 2 mL single dose of AA was administered IR to establish the UC model, except the control group, which received 2 mL normal saline IR. 48 h post-IR injection, the disease activity index (DAI) was estimated. Then, the rats were anesthetized with an overdose of thiopental sodium (50 mg/kg) [93]. The distal colonic parts were separated, opened, cleaned with normal saline, and assessed macroscopically to estimate the Wallace scores. Part of the colon was stored in formalin (10%) for histopathological and immunohistochemical examinations; the other part was homogenized in phosphate-buffered saline, centrifuged at 10,000 rpm/4 °C/15 min, and stored at −80 °C for biochemical analyses.

Of note, no animals met the predefined exclusion criteria; therefore, no animals were excluded from the analysis. No expected or unexpected adverse events were observed during the study. Humane endpoints were predefined and included severe lethargy, inability to access food or water, persistent recumbency, or body weight loss exceeding 20% of baseline. Animals reaching these criteria were to be humanely euthanized and excluded from further analysis. No animals met the predefined humane endpoint criteria during the study.

All animals were maintained under identical experimental conditions. Potential confounders were addressed by standardizing handling, treatment administration, and measurement timing across all groups.

The authors responsible for administering treatments were not blinded to group allocation due to the nature of the treatment. However, blinding was implemented during outcome assessment and data analysis. The investigator evaluating outcomes (Wallace scoring, biochemical assays, histopathological examination) was blinded to group allocation, and all samples were coded before analysis to ensure unbiased interpretation.

##### Estimation of the Activity of UC Disease

The severity of UC was assessed using the DAI, a composite scoring system based on changes in body weight, stool consistency, and the presence of rectal bleeding. Body weight loss was graded as follows: 0, no weight loss; 1, 1–5%; 2, 6–10%; 3, 11–20%; and 4, >20% reduction from the initial weight. Stool consistency was scored as 0 for normal stools, 1 for soft but formed stools, 2 for very soft stools, 3 for mild diarrhea, and 4 for severe diarrhea. Rectal bleeding was evaluated using a scale ranging from 0 to 4, where 0 indicated the absence of bleeding, 1 indicated a positive hemoccult test, 2 indicated visible traces of blood in the feces, 3 represented mild bleeding, and 4 represented severe bleeding. The final DAI score was determined by calculating the mean of the three individual parameter scores [6].

##### Macroscopic Examinations

The macroscopic damage of the colon was measured using Wallace scores according to the previous study by El-Akabawy and El-Sherif [6] (Table 8).

##### Calculation of Colon Weight-to-Length Ratio

To evaluate the degree of edematous tissues of the colon and the severity of UC in rats, the weight-to-length ratio (mg/cm) was calculated for each rat [94].

##### Measuring the Oxidative Stress Parameters in the Colonic Tissue Homogenate

The content of MDA, a lipid peroxidation byproduct, in the colonic tissue was measured using the BioVision colorimetric kit (Catalog No: K739-100, Milpitas, CA, USA). The enzymatic antioxidant enzyme SOD activity was measured using the BioVision colorimetric kit (Catalog No: K335-100, Milpitas, CA, USA). Nitric oxide (NO) tissue content was measured using the BioVision colorimetric kit (Catalog No: K262-200, Milpitas, CA, USA).

##### Measuring the Inflammatory Markers in Colonic Homogenate

ELISA kits were used to measure the tissue content of inducible NO synthase (iNOS) (Catalog No: NBP2-80257, Novus Biologicals, CO, USA), TNF-α (Catalog No: 438205, BioLegend, San Diego, CA, USA), IL-6 (Catalog no: SEA079Ra, CloudClone Corp., Katy, TX, USA), and NF-κB p65 (Catalog No: ER1186, FineTest, Wuhan, China).

The tissue content of COX-2 was measured using an ELISA kit (Catalog No: DEIA6222, CreativeDiagnostics, New York, NY, USA).

##### Measuring PGE2 in Colonic Homogenate

The tissue content of PGE2 was measured using an ELISA kit (Catalog No: ER1800, FineTest, Wuhan, China).

##### Measuring Toll-like Receptor-4 by Quantitative Reverse Transcription Polymerase Chain Reaction (QRT-PCR)

Toll-like receptor-4 (TLR4) was measured in colonic tissue homogenate using QRT-PCR, as adopted by Ahmed et al. [95]. In brief, total RNA was separated from the tissue homogenates using the SV Total RNA Isolation System (Promega, Madison, WI, USA). An RNA amount ranging from 0.5 to 2 µg was utilized for complementary DNA (cDNA) synthesis using the High-Capacity cDNA Reverse Transcription Kit (Catalog No. K1621, Fermentas, Hanover, MD, USA). Quantitative real-time PCR (qRT-PCR) was carried out using the StepOne™ Real-Time PCR System (Applied Biosystems, Waltham, MA, USA) equipped with software version 3.1. The qPCR protocol was optimized for annealing temperature specific to the primer pairs. The sequences of the primers were as follows:

TLR4—Forward: 5′-ATGCCAGTGTGTTTCTGCTC-3′;

Reverse: 5′-TGG AAG TCC TGA AGT AGC CAA-3′;

*β*-actin—Forward: 5′-GAGACCTTCAACACCCCAGC-3′;

Reverse: 5′-ATGTCACGCACGATTTCCC-3′.

##### Measuring Phosphorylated-IRAK1 (p-IRAK1) by Western Blot Analysis

The relative tissue content of phosphorylated-IRAK1 (p-IRAK1) (Thr387) was measured using Western blot analysis as adopted by Ahmed et al. [95]. The phosphorylated-IRAK1 (p-IRAK1) antibody was purchased from Affinity Biotech. (Catalog No: AF8009, Cincinnati, OH, USA). In brief, Proteins were extracted from tissue samples using RIPA lysis buffer and quantified with the Bradford assay (Bio Basic Inc., Markham, ON, Canada). After electrophoresis and transfer onto PVDF membranes, the samples were incubated overnight at 4 °C with primary antibodies. Following TBST washes, membranes were treated with HRP-conjugated goat anti-rabbit secondary antibodies at room temperature. Protein bands were detected using Clarity™ Western ECL substrate (Bio-Rad, USA) and visualized with a CCD camera system. Band intensity was analyzed with the Chemi Doc MP imaging system and normalized to *β*-actin.

##### Determination of Colonic Tissue Protein Content

Protein content in the colonic tissue was determined according to the method of Lowry et al. [96].

##### Histopathological Examination

Colonic tissue samples were rinsed with saline and fixed in 10% neutral-buffered formalin for 72 h. The tissues were subsequently trimmed, dehydrated through ascending grades of ethanol, cleared in xylene, and embedded in Paraplast^®^ embedding medium. Serial sections (5 μm thick) were prepared using a rotary microtome and stained for histopathological examination of the colonic wall architecture and associated pathological alterations. Tissue sections were stained by the following and examined:

1. Hematoxylin and Eosin as a general morphological staining method.
2. Alcian Blue pH 2.5 for demonstration of goblet cells acidic mucins.

All standard procedures for sample fixation and staining according to Culling et al. [97].

##### Statistical Analysis

Our data was expressed as mean (M) ± standard error (SEM). Data distribution was assessed for normality using the Shapiro–Wilk test. ANOVA test followed by Tukey’s Post hoc test was used to determine the significance between groups. A *p*-value < 0.05 was considered significant. The analysis was performed using GraphPad Prism, 8th version (GraphPad Software Inc., La Jolla, CA, USA).

### 4.8. Docking Study

The potential binding modes of key identified phytocompounds from the extracts of *C. gileadensis*, *S. incanescens*, and *S. parviflora* with TLR4, COX-1, and COX-2 using PDB ID: 2Z65, 1Q4G, and 3NT1 were investigated using molecular docking [88,89]. Docking simulations were performed with AutoDock Vina v1.2.6 [98], while ligand and protein preparation (conversion to pdbqt format) was carried out using OpenBabel [99]. Visualization and interaction analyses were conducted in Discovery Studio [100]. Protein preparation involved removal of crystallographic water molecules and co-crystallized ligands, while polar hydrogen atoms and Gasteiger partial charges were added prior to conversion into the PDBQT format. Protein protonation states were assigned assuming physiological pH = 7.4. Ligand structures were obtained from the PubChem database, converted into 3D conformations, energy-minimized using the MMFF94 force field, protonated at physiological pH = 7.4, and subsequently converted to PDBQT format.

AutoDock Vina applies a united atom scoring function with an Amber force field, integrating a gradient-based local search genetic algorithm with global optimization to predict ligand binding conformations. The docking search space was centered on the binding pocket defined by the co-crystallized ligand (PDB IDs: E55, BFL, and NPS). For TLR4/MD-2 (PDB ID: 2Z65), the grid box was centered at x = −18.5, y = 12.8, z = 4.2 with dimensions of 20 × 20 × 20 Å, while for COX-1 (PDB ID: 1Q4G) and COX-2 (PDB ID: 3NT1), the grid boxes were centered at (x = 23.4, y = 34.1, z = 21.6) and (x = 28.5, y = 20.3, z = 15.8), respectively, with identical box dimensions, extensive the active sites. The docking protocol followed previously reported procedures for these targets [91,92]. The exhaustiveness parameter was set to 8, while all other parameters were maintained at their default AutoDock Vina settings.

To validate the docking protocol, the native co-crystallized ligands (E55 for TLR4/MD-2, BFL for COX-1, and NPS for COX-2) were removed from their respective crystal structures and independently redocked into the corresponding binding sites. The resulting docking poses reproduced the experimental binding orientations with RMSD values below 1.5 Å, confirming the reliability of the docking protocol. Docking results were evaluated based on predicted binding affinities and ligand–protein interaction profiles generated using Discovery Studio Visualizer.

## 5. Study Limitation

While the results of this study show potential, there are several important concerns that need to be addressed before these findings can be applied in clinical settings. Firstly, the AA-induced colitis model only reflects an acute chemical injury, which does not adequately mimic the chronic, relapsing, and multifactorial aspects of human ulcerative colitis. Secondly, the study focused solely on female rats, leaving the possibility of sex-related differences in therapeutic responses unexamined. Additionally, the changes observed in the TLR4/IRAK/NF-κB and NF-κB/iNOS/NO pathways were based on biochemical and molecular assessments as well as molecular docking analyses, but causal relationships were not confirmed using pathway-specific inhibitors or genetic methods. Furthermore, the complex nature of the crude extracts, particularly the metabolite-rich *C. gileadensis* extract, creates challenges for standardization and reproducibility; additionally, the bioavailability of several identified bioactive flavonoids may be hindered by issues like poor solubility, instability, and rapid metabolism. Moreover, most phytochemicals were assigned with probable or tentative confidence based on LC-MS/MS data, whereas confirmed identifications using authentic reference standards were not achieved. Finally, this study did not cover pharmacokinetics, formulation, or long-term safety. Future studies should include bioactivity-guided fractionation and isolation of the major active constituents, validation of their molecular targets, chronic colitis models, optimized formulations, and comprehensive pharmacokinetic and toxicity evaluations before clinical translation.

## 6. Conclusions

This study provides a comprehensive phytochemical and pharmacological investigation of *C. gileadensis*, *S. incanescens*, and *S. parviflora*, revealing numerous primary and secondary metabolites that are reported in these species for the first time. The methanolic extracts of the aerial parts of the three plants exerted significant dose-dependent protective effects against acetic acid-induced ulcerative colitis in rats, supporting their traditional medicinal use and highlighting their potential as sources of anti-inflammatory agents. Among the tested extracts, *C. gileadensis* exhibited the most potent activity, showing marked improvement in disease activity, preservation of colonic architecture, restoration of antioxidant defenses, and attenuation of inflammatory mediators, exhibiting efficacy comparable to the standard drug, sulfasalazine, at a dose of 500 mg/kg. Mechanistic investigations indicated that the observed protective effects are partially mediated through suppression of the TLR4/IRAK/NF-κB and NF-κB/iNOS/NO signaling pathways, in addition to selective COX-2 inhibitory activity. However, these findings were obtained in an acute AA-induced ulcerative colitis model using female rats and crude, chemically complex extracts rather than isolated compounds, and therefore should be considered preliminary evidence of therapeutic potential. Molecular docking analyses provided a preliminary in silico support for the biological findings, suggesting a hypothesis that the superior activity of *C. gileadensis* may be linked to its unique triterpenoid and flavonoid constituents. These compounds were predicted to interact favorably with key inflammatory targets, potentially supporting the stabilization of the TLR4/MD-2 complex in an inactive conformation, demanding further experimental validation. In contrast, the activity of *S. incanescens* and *S. parviflora* appeared to be associated mainly with phenolic amides and selective COX-2 inhibition. These findings support the traditional medicinal use of these medicinal plants and identify *C. gileadensis* as the most promising candidate for further investigation. Nevertheless, future studies are required to confirm the proposed mechanisms, isolate and characterize the principal active constituents, evaluate long-term pharmacokinetics and safety, and develop standardized formulations and advanced delivery systems to enable the potential clinical translation for ulcerative colitis management.

## Figures and Tables

**Table 1:** Comparative phytochemical profile of tentatively identified key bioactive and marker metabolites identified by LC-MS/MS in the methanolic extracts of *C. gileadensis*, *S. incanescens*, and *S. parviflora*.

Compound Name	R_t_ (min)	Formula	*C. gileadensis*		*S. incanescens*		*S. parviflora*	
			Code ^1^	Precursor Ion (Mode)	Code ^1^	Precursor Ion (Mode)	Code ^1^	Precursor Ion (Mode)
**I. Flavonoids**								
3,3′,4′,5,7-Pentahydroxyflavan; Epicatechin	1.24	C_15_H_14_O_6_	—	—	**2.2.1**	289.0741 (−)	—	—
Okanin-4′-*O*-glucoside	5.56–6.51	C_21_H_22_O_11_	**1.1.2**	449.113 (−)	—	—	**3.1.1**	449.1173 (−)
Gossypin	6.92	C_21_H_20_O_13_	**1.1.3**	481.0960 (+)	—	—	—	—
Hesperidin	7.31	C_28_H_34_O_15_	**1.1.4**	609.1549 (−)	**2.2.4**	609.1559 (−)	—	—
Delphinidin-3-*O*-beta-glucopyranoside	7.54	C_21_H_21_O_12_	**1.1.6**	463.0931 (−)	—	—	—	—
Daidzein-8-*C*-glucoside	7.67	C_21_H_20_O_9_	—	—	**2.2.5**	417.1135 (+)	—	—
Hyperoside	7.56	C_21_H_20_O_12_	**1.1.7**	465.1018 (+)	—	—	—	—
(+)-Taxifolin	8.08	C_15_H_12_O_7_	**1.1.11**	303.0534 (−)	—	—	—	—
Cyanidin-3-glucoside	8.16	C_21_H_21_O_11_	—	—	—	—	**3.1.8**	449.1065 (+)
Syringetin-3-*O*-galactoside	8.17	C_23_H_24_O_13_	—	—	**2.2.7**	507.2294 (−)	—	—
Isorhamnetin-3-*O*-glucoside	8.19–8.28	C_22_H_22_O_12_	**1.1.12**	477.108 (−)	**2.2.8**	477.1106 (−)	**3.1.9**	477.1133 (−)
Myricetin	8.90	C_15_H_10_O_8_	**1.1.14**	317.0327 (−)	—	—	—	—
Luteolin	10.11–10.12	C_15_H_10_O_6_	**1.1.19**	285.0425 (−); 287.0551 (+)	**2.2.12**	285.0444 (−)	**3.1.10**	285.0439 (−)
Quercetin	10.18	C_15_H_10_O_7_	**1.1.20**	301.0371 (−); 303.0495 (+)	—	—	—	—
Apigenin	10.21–11.20	C_15_H_10_O_5_	**1.1.22**	271.0601 (+)	**2.2.13**	269.0458 (−)		—
Naringenin	11.23	C_15_H_12_O_5_	**1.1.23**	271.0633 (−); 273.0753 (+)	—	—	—	—
Kaempferide	11.49	C_16_H_12_O_6_	**1.1.26**	299.0586 (−); 301.0714 (+)	—	—	**3.1.11**	299.061 (−)
Hesperetin	11.64	C_16_H_14_O_6_	**1.1.24**	301.0738 (−)	—	—	—	—
3′-Methoxy-4′,5,7-trihydroxyflavonol; Isorhamnetin	11.67–11.69	C_16_H_12_O_7_	**1.1.25**	315.0544 (−); 317.0653 (+)	**2.2.14**	315.0547 (−)	**3.1.12**	315.0551 (−)
Peonidin; 3,4′,5,7-tetrahydroxy-3′-methoxyflavylium	12.41	C_16_H_13_O6^+^	—	—	**2.2.15**	301.0701 (+)	—	—
**II. Triterpenes**								
Salsolin A	12.60	C_30_H_48_O_6_	—	—	**2.4.2**	503.3431 (−)	—	—
Salsolin B	15.20	C_30_H_48_O_6_	—	—	**2.4.4**	503.3441 (−)	—	—
Oleanonic aldehyde	17.39	C_30_H_46_O_2_	**1.9.1**	439.3563 (+)	—	—	—	—
11-Oxo-oleanonic acid	19.42	C_30_H_44_O_4_	**1.9.2**	469.3307 (+)	—	—	—	—
11-Oxo-ursonic acid	20.15	C_30_H_44_O_4_	**1.9.3**	469.3307 (+)	—	—	—	—
Oleanonic acid	21.03	C_30_H_46_O_3_	**1.9.4**	455.3505 (+)	—	—	—	—
Amasterol	22.46	C_28_H_44_O_2_	—	—	**2.10.2**	411.3173 (−)	—	—
Stigmasterol	22.52	C_29_H_48_O	**1.9.9**	413.3763 (+)	—	—	—	—
Commigileadin A	22.53	C_30_H_46_O_3_	**1.9.5**	455.351 (+)	—	—	—	—
Urosonic acid	22.80	C_30_H_46_O_3_	**1.9.6**	455.3505 (+)	—	—	—	—
Canophyllal	23.07	C_30_H_48_O_2_	**1.9.7**	441.3727 (+)	—	—	—	—
**III. Nitrogenous**								
Vulgaxanthin I	1.31	C_14_H_17_N_3_O_7_	—	—	**2.1.1**	340.122 (+)	—	—
Tyramine-*O*-*β*-D-glucoside	6.32	C_14_H_21_NO_6_	—	—	**2.1.2**	298.1323 (−)	—	—
Salsolidine or *N*-Methylisosalsolidine	6.33	C_12_H_17_NO_2_	—	—	**2.1.3**	208.1324 (+)	—	—
*N*-(3′,4′-Dimethoxy-cinnamoyl)-norepinephrine	6.76	C_19_H_21_NO_6_	—	—	**2.1.4**	360.1432 (+)	—	—
Cimicifugamide; *N*-(*Trans*-4-*O*-β-D-glucopyranoside feruloyl)-3′-*O*-methyldopamine	7.82	C_25_H_31_NO_10_	—	—	**2.1.8**	506.2007 (+)	—	—
2′-Hydroxy-3″-methyoxymoupinamide; (*E*)-*N*-(2-hydroxy-2-(3-hydroxy-4-methoxyphenyl)ethyl)-3-(4-hydroxy-3-methoxyphenyl)acrylamide	8.24	C_19_H_21_NO_6_	—	—	**2.1.9**	360.1436 (+)	—	—
*N*-Caffeoyltyramine	8.62	C_17_H_17_NO_4_	—	—	**2.1.11**	300.1223 (+)	—	—
*N*-*Trans*-feruloyl tyramine (Moupinamide)	9.39	C_18_H_19_NO_4_	—	—	**2.1.12**	314.1384 (+)	**3.7.1**	—
*N*-*Trans*-feruloyl-3-*O*-methyldopamine; *N-trans*-feruloyl-4′-*O*-methyldopamine	9.59	C_19_H_21_NO_6_	—	—	**2.1.13**	344.1492 (+)	—	—
Feruloyltyramine	9.71	C_18_H_19_NO_4_	—	—	—	—	**3.7.1**	314.1375 (+)
(*E*)-3-(4-Hydroxy-3-methoxyphenyl)-*N*-(4-methoxyphenethyl)acrylamide	10.36	C_19_H_21_NO_4_	—	—	**2.1.16**	328.1534 (+)	—	—
Erucamide	26.06–26.07	C_22_H_43_NO	—	—	**2.1.17**	—	**3.7.2**	338.3408 (+)
**IV. Phenolics**								
Chlorogenic acid	4.71	C_16_H_18_O_9_	**1.2.1**	353.091 (−); 355.1020 (+)	—	—	—	—
Vanillic acid glucoside	4.99–5.01	C_14_H_18_O_9_	**1.2.2**	329.0906 (−)	**2.3.2**	329.0911 (−)	**—**	—
Esculetin	6.36		**1.3.1**	177.0203 (−)		—		—
Scopoletin	7.26–8.02	C_10_H_8_O_4_	**1.3.2**	193.0494 (+)	**2.3.5**	193.0497 (+)	—	—
p-Coumaric acid	7.50	C_9_H_8_O_3_	**1.2.5**	163.0431 (−)	—	—	—	—
Sinapyl aldehyde	9.59–9.66	C_11_H_12_O_4_	**1.2.4**	207.0698 (−)	**2.3.8**	207.0696 (−)	**—**	—
**V. Other Classes**								
*E*-3,4,5′-Trihydroxy-3′-glucopyranosylstilbene	9.50	C_20_H_22_O_9_	**1.4.1**	405.1256 (−)	**2.3.7**	405.1601 (−)	—	—
L-Glutamine	1.20	C_5_H_10_N_2_O_3_	—	—	—	—	**3.3.1**	147.0757 (+)
Norvaline	1.30	C_5_H_11_NO_2_		—		—	**3.3.6**	116.0729 (−)
L-Tryptophan	4.86–4.87	C_11_H_12_N_2_O_2_	—	—	—	—	**3.3.13**	203.0845 (−); 205.0967 (+)
Gamma-Linolenic acid	17.77	C_18_H_30_O_2_	**1.6.3**	277.219 (−)	—	—	**3.5.3**	277.2216 (−)

^1^ Compound codes in Tables S1–S3, including complete LC–MS/MS identification data, including theoretical (reference) masses, key MS/MS fragment ions, MassBank accession numbers, and supporting spectral information, are provided in the Supplementary Material.

**Table 2:** Total phenolic and flavonoid contents of the investigated methanolic extracts and the corresponding aerial parts of examined plants.

Extract	TPC (µg GAE/mg Extract)	TPC (mg GAE/g Dry Plant)	TFC (µg QE/mg Extract)	TFC (mg QE/g Dry Plant)
*C. gileadensis*	10.65 ± 0.33	1.27 ± 0.04	13.67 ± 1.00	1.63 ± 0.12
*S. incanescens*	2.48 ± 0.20	0.22 ± 0.02	6.63 ± 0.08	0.58 ± 0.01
*S. parviflora*	26.29 ± 2.50	5.68 ± 0.54	11.35 ± 0.71	2.45 ± 0.15

**Table 3:** The inhibitory effect of the examined extracts on LPS-induced IL-6 and TNF-α production in RAW264.7 macrophages ^1^.

Sample	IL-6 (pg/mL)	IL-6 (Relative to LPS)	TNF-α (pg/mL)	TNF-α (Relative to LPS)
LPS	247.66 ± 6.80	1	1772.79 ± 48.64	1
*C. gileadensis* + LPS	26.36 ± 0.72	0.11	279.67 ± 7.64	0.16
*S. incanescensis* + LPS	67.41 ± 1.85	0.27	532.46 ± 14.64	0.30
*S. parviflora* + LPS	118.34 ± 3.25	0.48	859.46 ± 23.62	0.48
Celecoxib + LPS	28.83 ± 0.79	0.2	257.65 ± 7.14	0.15

^1^ RAW264.7 cells were stimulated with lipopolysaccharide (LPS) in the presence or absence of the indicated compounds. Culture supernatants were collected, and cytokine concentrations were measured by ELISA (ab178013 for IL-6; ab208348 for TNF-α). Data are presented as mean ± SEM (*n* = 2 replicates). LPS is considered a positive control. Celecoxib was used as a reference anti-inflammatory compound. Relative values were calculated by setting the LPS control to 1.00.

**Table 4:** Inhibitory effects of the examined extracts on COX-1 and COX-2 enzyme activity, selectivity index, and NO radical scavenging activity ^1^.

Sample	COX-1 IC_50_ (µM)	COX-2 IC_50_ (µM)	Selectivity Index (SI) (COX-1/COX-2)	NO Scavenging IC_50_ (µg/mL)
*C. gileadensis*	8.70 ± 0.29	1.96 ± 0.07	4.44	27.31 ± 1.15
*S. incanescensis*	13.76 ± 0.46	7.91 ± 0.27	1.74	43.35 ± 1.75
*S. parviflora*	45.18 ± 1.52	34.06 ± 1.15	1.33	52.24 ± 2.11
Celecoxib	22.85 ± 0.84	0.41 ± 0.02	55.33	-
Ascorbic acid	-	-	-	37.92 ± 1.53

^1^ Celecoxib is shown as a reference selective COX-2 inhibitor, while ascorbic acid was used as the reference standard for the NO scavenging assay. NO scavenging Activity was determined using the Griess reagent method with SNP as the NO source. Data are presented as mean ± SEM (*n* = 3, 2 replicates). The selectivity index (SI) was calculated as IC_50_ (COX-1)/IC_50_ (COX-2).

**Table 5:** Results of molecular docking of the crystallized ligand and key identified bioactive compounds in conjunction with TLR4 (PDBID: 2Z65).

Docked Compounds	S (kcal/mol)	RMSD (Å)	Plant Source
Crystallized ligand; Eritoran	−10.1264	1.59	-
Isorhamnetin	−6.1780	0.897	*C. gileadensis*, *S. incanescens*, *S. parviflora*
Syringetin	−6.4448	1.3414	*S. incanescens*
Daidzein-8-*C*-glucoside	−6.0153	1.5229	*S. incanescens*
*N*-Feruloyltyramine (Moupinamide)	−6.5013	1.2544	*S. incanescens*, *S. parviflora*
*N*-Caffeoyltyramine	−6.1018	1.7405	*S. incanescens*
(*E*)-3-(4-Hydroxy-3-methoxyphenyl)-*N*-(4-methoxyphenethyl) acrylamide	−6.5671	1.6482	*S. incanescens*
2′-Hydroxy-3″-methyoxymoupinamide	−6.5108	1.3620	*S. incanescens*
*N*-*Trans*-feruloyl-3-*O*-methyldopamine; *N-trans*-feruloyl-4′-*O*-methyldopamine	−6.6437	0.7423	*S. incanescens*
*N*-(3′,4′-Dimethoxy-cinnamoyl)-norepinephrine	−6.1253	1.0882	*S. incanescens*
Erucamide (fatty acid amide)	−7.1364	1.2028	*S. incanescens*, *S. parviflora*
Commigileadin A	−6.6811	1.4608	*C. gileadensis*
Urosonic acid	−6.3571	1.1278	*C. gileadensis*
Canophyllal	−6.2468	1.4735	*C. gileadensis*
Amasterol	−6.6611	1.6326	*S. incanescens*
Stigmasterol	−6.7817	1.1190	*C. gileadensis*

**Table 6:** Results of molecular docking of the crystallized ligand and key identified bioactive compounds in conjunction with COX-1 (PDBID: 1Q4G) and COX-2 (PDB ID: 3NT1).

Docked Compounds	COX-1 (PDB: 1Q4G)		COX-2 (PDB: 3NT1)		Binding Preference	Plant Source
	S (kcal/mol)	RMSD (Å)	S (kcal/mol)	RMSD (Å)		
Crystallized ligand	−7.66	1.17	−7.89	0.9983		-
Luteolin	−5.01	0.79	−5.48	1.4193	COX-2	*C. gileadensis*, *S. incanescens*, *S. parviflora*
Kaempferide	−5.79	1.36	−5.28	1.006	COX-1	*C. gileadensis*, *S. incanescens*, *S. parviflora*
Acacetin	−5.45	1.32	−5.19	0.85	COX-1	*C. gileadensis*, *S. incanescens*
Peonidin	−5.26	1.49	−5.26	1.52	COX-2	*S. incanescens*
Syringetin	−5.61	0.96	−5.10	1.1	COX-1	*S. incanescens*
*N*-Feruloyltyramine (Moupinamide)	−5.84	1.66	−6.24	1.04	COX-2	*S. incanescens*, *S. parviflora*
*N*-Caffeoyltyramine	−5.42	0.98	−6.48	1.07	COX-2	*S. incanescens*
(*E*)-3-(4-Hydroxy-3-methoxyphenyl)-*N*-(4-methoxyphenethyl) acrylamide	−6.52	0.88	−6.69	0.84	COX-2	*S. incanescens*
2′-Hydroxy-3″-methyoxymoupinamide	−6.87	0.87	−6.84	1.05	COX-1	*S. incanescens*
*N-trans*-feruloyl-4′-*O*-methyldopamine	−6.55	1.05	−7.09	1.03	COX-2	*S. incanescens*
Chlorogenic acid	−5.07	1.65	−5.19	1.45	COX-2	*C. gileadensis*
Scopoletin	−5.85	1.36	−5.68	0.69	COX-1	*C. gileadensis*, *S. incanescens*
Daphnetin	−5.18	1.51	−5.09	0.79	COX-1	*C. gileadensis*

**Table 7:** Comparative summary of the investigated Arabian Desert plant extracts: *C. gileadensis*, *S. incanescens* and *S. parviflora* and their observed bioactivity.

Plant Extract	Dominant Chemical Classes	No. of Compounds	Key Marker Metabolites	Proposed Bioactivity Relevance
*Commiphora gileadensis* (Mecca myrrh)	Flavonoids (Flavonols), Triterpenoids (Oleanane/Ursane), Phenolic acids.	59	Commigileadin A, canophyllal, gossypin, isorhamnetin.	Highest efficacy: Observed in vivo attenuation of TLR4, NF-κB, and iNOS pathways. Molecular docking suggested potential multi-target interactions with TLR4/MD-2 and COXs, owing to its unique triterpenoids and flavonoids.
*Salsola incanescens* (Al-khithraf)	Phenolic amides, Flavonoids, Polar Triterpenoids.	69	*N*-*trans*-Feruloyl tyramine (moupinamide), salsolins A/B, salsolic acid.	Moderate efficacy: Observed in vitro and in vivo anti-inflammatory activity. In silico predictions suggest a more targeted binding preference for COX-2 via the specific phenolic amide content.
*Savignya parviflora* (Gilgilan)	Amino acids (Brassicaceae-type), Flavonoids, Alkyl amides.	41	Feruloyltyramine, erucamide, okanin-4′-*O*-glucoside.	Weakest efficacy: An amino acid-rich profile, which indicated its nutritional value primarily. Exhibited the lowest potency across all inflammatory markers. Molecular docking showed generally lower affinities than markers from other extracts.

**Table 8:** Macroscopic evaluation scale for UC.

Score	Criterion
0	No ulcer or inflammation
1	No ulcer with local hyperemia
2	Ulceration without hyperemia
3	Ulceration and inflammation at one site only
4	Two or more sites of ulceration and inflammation
5	Ulceration extending more than 2 cm

## Data Availability

The original data are included in the manuscript, and further queries can be directed to the corresponding author.

## Supplementary Materials

The following supporting information can be downloaded at https://www.mdpi.com/article/10.3390/ph19081178/s1: Tables S1–S3: LC–MS/MS-based phytochemical profiling of *C. gileadensis*, *S. incanescens*, and *S. parviflora* extracts. Figures S1–S21: TOF-MS/MS spectra and proposed fragmentation patterns of selected identified metabolites. Figures S22 and S23: Calibration curves for total phenolic content (TPC) and total flavonoid content (TFC), respectively. Tables S4 and S5: TPC and TFC of the methanolic extracts. Figure S24 and Table S6: Binding interactions and molecular docking results of selected compounds with TLR4/MD-2 (PDB ID: 2Z65). Figures S25 and S26: Two- and three-dimensional binding interaction poses of selected compounds within the COX-1 (PDB ID: 1Q4G) and COX-2 (PDB ID: 3NT1) active sites, respectively [101,102,103,104,105,106,107,108,109].

## Abbreviations

The following abbreviations are used in this manuscript:

AA	Acetic acid
AME	Aerial parts methanol extract
ANOVA	Analysis of variance
CAT	Catalase
cDNA	Complementary DNA
COX-1	Cyclooxygenase-1
COX-2	Cyclooxygenase-2
DAI	Disease activity index
DAMPs	Damage-associated molecular patterns
DMSO	Dimethyl sulfoxide
ELISA	Enzyme-linked immunosorbent assay
GAE	Gallic acid equivalent
GIT	Gastrointestinal tract
H&E	Hematoxylin and eosin
HO-1	Heme Oxygenase-1
HRP	Horseradish peroxidase
IACUC	Institutional Animal Care and Use Committee
IBD	Inflammatory bowel disease
IC_50_	Half maximal inhibitory concentration
IL-6	Interleukin-6
iNOS	Inducible nitric oxide synthase
IRAK	Interleukin-1 receptor-associated kinase
IR	Intrarectal
LC-MS/MS	Liquid chromatography–tandem mass spectrometry
LPS	Lipopolysaccharide
MAMPs	Microbe-associated molecular patterns
MAPK	Mitogen-Activated Protein Kinase
MDA	Malondialdehyde
MS	Mass spectrometry
MD-2	Myeloid Differentiation factor 2
NF-κB	Nuclear factor-kappa B
NO	Nitric oxide
Nrf2	Nuclear Factor Erythroid 2-Related Factor 2
OECD	Organisation for Economic Co-operation and Development
PBS	Phosphate-buffered saline
PCR	Polymerase chain reaction
PGE2	Prostaglandin E2
p-IRAK1	Phosphorylated interleukin-1 receptor-associated kinase 1
PO	Per os (oral administration)
qRT-PCR	Quantitative real-time polymerase chain reaction
QTOF-MS/MS	Quadrupole time-of-flight tandem mass spectrometry
QE	Quercetin equivalent
RAW264.7	Murine macrophage cell line
RNA	Ribonucleic acid
ROS	Reactive oxygen species
RMSD	Root mean square deviation
R*_t_*	Retention time
SD	Standard deviation
SEM	Standard error of the mean
SI	Selectivity index
SNP	Sodium nitroprusside
SOD	Superoxide dismutase
TBST	Tris-buffered saline with Tween 20
TIC	Total ion chromatogram
TLR4	Toll-like receptor 4
TNF-α	Tumor necrosis factor-alpha
TFC	Total flavonoid content
TPC	Total phenolic content
UC	Ulcerative colitis
UPLC	Ultra-performance liquid chromatography

## References

[1] Feuerstein J.D., Cheifetz A.S. (2014). Ulcerative colitis: Epidemiology, diagnosis, and management. Mayo Clin. Proc..

[2] Fabia R., Willén R., Ar’Rajab A., Andersson R., Ahrén B., Bengmark S. (1992). Acetic acid-induced colitis in the rat: A reproducible experimental model for acute ulcerative colitis. Eur. Surg. Res..

[3] Ibrahim H.A., Elshaarawy F.S., Abd-elhady M.I.S., Hamdy W., Ali M.E., Ahmed A.A., El-Sayed E.K. (2026). *Pimenta racemosa* extract ameliorates chemically induced ulcerative colitis in rats by suppressing inflammation and oxidative stress. Inflammopharmacology.

[4] Randhawa P.K., Singh K., Singh N., Jaggi A.S. (2014). A review on chemical-induced inflammatory bowel disease models in rodents. Korean J. Physiol. Pharmacol..

[5] Ghasemi-Dehnoo M., Lorigooini Z., Amini-Khoei H., Sabzevary-Ghahfarokhi M., Rafieian-Kopaei M. (2023). Quinic acid ameliorates ulcerative colitis in rats, through the inhibition of two TLR4-NF-κB and NF-κB-INOS-NO signaling pathways. Immun. Inflamm. Dis..

[6] El-Akabawy G., El-Sherif N.M. (2019). Zeaxanthin exerts protective effects on acetic acid-induced colitis in rats via modulation of pro-inflammatory cytokines and oxidative stress. Biomed. Pharmacother..

[7] Aslam N., Lo S.W., Sikafi R., Barnes T., Segal J., Smith P.J., Limdi J.K. (2022). A review of the therapeutic management of ulcerative colitis. Ther. Adv. Gastroenterol..

[8] Shaito A., Thuan D.T.B., Phu H.T., Nguyen T.H.D., Hasan H., Halabi S., Abdelhady S., Nasrallah G.K., Eid A.H., Pintus G. (2020). Herbal Medicine for Cardiovascular Diseases: Efficacy, Mechanisms, and Safety. Front. Pharmacol..

[9] Mokaizh A.A.B., Hamid A.N., Yunus R.M., Elnour A.A., Modather R.H. (2024). Phytochemical and pharmacological activities of *Commiphora gileadensis*: A review. J. Chem. Eng. Ind. Biotechnol..

[10] Eslamieh J. (2011). *Commiphora* *gileadensis*. Cact. Succ. J..

[11] Abdallah H., Mohamed G., Ibrahim S.R.M., Koshak A., Alnashri I., Alghamdi A., Aljohani A., Khairy A. (2022). Commigileadin A: A new triterpenoid from *Commiphora gileadensis* aerial parts. Pharmacogn. Mag..

[12] Iluz D., Hoffman M., Gilboa-Garber N., Amar Z. (2010). Medicinal properties of *Commiphora gileadensis*. Afr. J. Pharm. Pharmacol..

[13] Hanif Z., Ali H.H., Rasool G., Tanveer A., Chauhan B.S. (2018). Genus Salsola: Its benefits, uses, environmental perspectives and future aspects—A review. J. Rangel. Sci..

[14] ElNaggar M.H., Eldehna W.M., Abourehab M.A., Abdel Bar F.M. (2022). The old world salsola as a source of valuable secondary metabolites endowed with diverse pharmacological activities: A review. J. Enzym. Inhib. Med. Chem..

[15] Mollaei S. (2021). Metabolic Profiling and Inhibitory Properties of Different Parts of *Salsola Vermiculata* Towards Acetylcholinesterase and α-glucosidase. Res. Sq..

[16] Neghabi M. (2021). Introduction of *Salsola incanescens* as a Native Species with a Medicinal, Economic, and Suitable Value for Plantation in Arid and Semi-Arid Regions of Iran. Turk. J. Comput. Math. Educ..

[17] El-Raey M.A., Ahmed G.F., El Hawary S.S.E. (2025). An updated overview on certain plants of family Brassicaceae: Traditional uses, Phytochemistry and Pharmacological activities. Egypt. J. Chem..

[18] Alqaraawi S.S., Almujaydil M.S. (2025). Nutritional properties and potential therapeutic uses of *Brassica nigra*. Discov. Food..

[19] Zhao J., Zhang X., Li F., Lei X., Ge L., Li H., Zhao N., Ming J. (2024). The effects of interventions with glucosinolates and their metabolites in cruciferous vegetables on inflammatory bowel disease: A review. Foods.

[20] Gandhi G.R., Mohana T., Athesh K., Hillary V.E., Vasconcelos A.B.S., Farias de Franca M.N., Montalvão M.M., Ceasar S.A., Jothi G., Sridharan G. (2023). Anti-inflammatory natural products modulate interleukins and their related signaling markers in inflammatory bowel disease: A systematic review. J. Pharm. Anal..

[21] Zhao Y., Wu J., Liu X., Chen X., Wang J. (2025). Decoding nature: Multi-target anti-inflammatory mechanisms of natural products in the TLR4/NF-κB pathway. Front. Pharmacol..

[22] Zhao Y.Q., Zhang Y., Qin Y., Zhang R.Y., Wang J.P. (2026). Cedrol ameliorates ulcerative colitis via myeloid differentiation factor 2-mediated inflammation suppression, with barrier restoration and microbiota modulation. World J. Gastroenterol..

[23] Qin X., Lu Y., Luo Y., Cui Y., Zhao K., He Y., ur Rahman M.A., Min S., Wang W., Yang F. (2025). Alfalfa Flavonoids Mitigate Salmonella-Induced Colitis via the Keap1-Nrf2 and TLR4/NF-κB/COX-2 Pathways. Food Front..

[24] Liu M., Guan G., Wang Y., Lu X., Duan X., Xu X. (2024). p-Hydroxy benzaldehyde, a phenolic compound from Nostoc commune, ameliorates DSS-induced colitis against oxidative stress via the Nrf2/HO-1/NQO-1/NF-κB/AP-1 pathway. Phytomedicine.

[25] Guo F., Zhang S. (2025). Chinese Medicine-Derived Natural Compounds and Intestinal Regeneration: Mechanisms and Experimental Evidence. Biomolecules.

[26] Xue H.-H., Li J.-J., Li S.-F., Guo J., Yan R.-P., Chen T.-G., Shi X.-H., Wang J.-D., Zhang L.-W. (2023). Phillygenin Attenuated Colon Inflammation and Improved Intestinal Mucosal Barrier in DSS-induced Colitis Mice via TLR4/Src Mediated MAPK and NF-κB Signaling Pathways. Int. J. Mol. Sci..

[27] Schymanski E.L., Jeon J., Gulde R., Fenner K., Ruff M., Singer H.P., Hollender J. (2014). Identifying small molecules via high resolution mass spectrometry: Communicating confidence. Environ. Sci. Technol..

[28] Al-Howiriny T., Al-Sohaibani M., Al-Said M., Al-Yahya M., El-Tahir K., Rafatullah S. (2005). Effect of *Commiphora opobalsamum* (L.) Engl. (Balessan) on experimental gastric ulcers and secretion in rats. J. Ethnopharmacol..

[29] Shehab N.G., Abu-Gharbieh E. (2014). Phenolic Profiling and Evaluation of Contraceptive Effect of the Ethanolic Extract of *Salsola imbricata* Forssk. in Male Albino Rats. Evid. Based Complement. Altern. Med..

[30] Alanazi F.K., Hashad N., Ahmed A.A., Ibrahim H.A., Ibrahim R.R., Abdelhady M.I., Haggag E.G., Abdel Bar F.M. (2026). Chemical Composition and Biological Activities of Diverse Products from *Commiphora gileadensis*: A Comparative Review. Pharmaceuticals.

[31] Neumann S., Meier R., Wenk M., Elapavalore A., Nishioka T., Schulze T., Stravs M., Tsugawa H., Matsuda F., Schymanski E.L. (2026). MassBank: An open and FAIR mass spectral data resource. Nucleic Acids Res..

[32] Elbana R.M., Taie H.A., Moustafa A.M.Y., Marzouk M. (2024). LC-MS/MS analyses of *Khaya grandifoliola* and in vitro Antioxidant Activity and Cytotoxicity of *Khaya senegalensis* and *Khaya grandifoliola* against Ehrlich Ascites Carcinoma Cells. Egypt. J. Chem..

[33] Truchado P., Vit P., Heard T.A., Tomás-Barberán F.A., Ferreres F. (2015). Determination of interglycosidic linkages in *O*-glycosyl flavones by high-performance liquid chromatography/photodiode-array detection coupled to electrospray ionization ion trap mass spectrometry. Its application to *Tetragonula carbonaria* honey from Australia. Rapid Commun. Mass. Spectrom..

[34] Abdl Aziz F.T., Temraz A.S., Hassan M.A., Sciences M. (2024). Metabolites profiling by LC-ESI-MS/MS technique and in-vitro antioxidant activity of *Bauhinia madagascariensis* Desv. and *Bauhinia purpurea* L. aerial parts cultivated in Egypt: A comparative study. Al-Azhar Int. J. Pharm. Sci..

[35] Badawy S.A., Hassan A.R., Abu Bakr M.S., Mohammed A.E.-S.I. (2025). UPLC-qTOF-MS/MS profiling of phenolic compounds in *Fagonia arabica* L. and evaluation of their cholinesterase inhibition potential through in-vitro and in-silico approaches. Sci. Rep..

[36] Yuzuak S., Ballington J., Li G., Xie D.-Y. (2024). High-Performance Liquid Chromatography–Quadrupole Time-of-Flight Tandem Mass Spectrometry-Based Profiling Reveals Anthocyanin Profile Alterations in Berries of Hybrid Muscadine Variety FLH 13-11 in Two Continuous Cropping Seasons. Agronomy.

[37] Lech K. (2020). Universal analytical method for characterization of yellow and related natural dyes in liturgical vestments from Krakow. J. Cult. Herit..

[38] Shi F., Pan H., Lu Y., Ding L. (2018). An HPLC–MS/MS method for the simultaneous determination of luteolin and its major metabolites in rat plasma and its application to a pharmacokinetic study. J. Sep. Sci..

[39] Attia R.A., El-Dahmy S.I., Abouelenein D., Abdel-Ghani A.E.-S. (2022). LC-ESI-MS profile, cytotoxic, antioxidant, insecticidal and antimicrobial activities of wild and in vitro propagated *Tanacetum sinaicum* Del. ex DC. Zagazig J. Pharm. Sci..

[40] Dantas-Medeiros R., Furtado A.A., Zanatta A.C., Torres-Rêgo M., Lourenço E.M.G., Alves J.S.F., Galinari É., de Oliveira Rocha H.A., Guerra G.C.B., Vilegas W. (2021). Mass spectrometry characterization of *Commiphora leptophloeos* leaf extract and preclinical evaluation of toxicity and anti-inflammatory potential effect. J. Ethnopharmacol..

[41] Huang Z., Hashida K., Makino R., Ohara S., Amartey S., Gillah P. (2010). Flavonoids with antifungal activity from heartwood of Tanzanian wood species: *Commiphora mollis* (Burseraceae). Int. Wood Prod. J..

[42] Al-Abdallat A.M., Adayileh B.K., Sawwan J.S., Shibli R., Al-Qudah T.S., Abu-Irmaileh B., Albdaiwi R.N., Almaliti J., Bustanji Y. (2023). Secondary metabolites profiling, antimicrobial and cytotoxic properties of *Commiphora gileadensis* L. leaves, seeds, callus, and cell suspension extracts. Metabolites.

[43] Abdel-Kader M.S., Ibnouf E.O., Alqarni M.H., AlQutaym A.S., Salkini A.A., Foudah A.I. (2022). Terpenes from the fresh stems of *Commiphora gileadensis* with antimicrobial activity. Rec. Nat. Prod..

[44] Abbas F.A., Al-Massarany S.M., Khan S., Al-Howiriny T.A., Mossa J.S., Abourashed E.A. (2007). Phytochemical and biological studies on Saudi *Commiphora opobalsamum* L.. Nat. Prod. Res..

[45] Van der Doelen G.A., Van den Berg K.J., Boon J.J., Shibayama N., De La Rie E.R., Genuit W.J.L. (1998). Analysis of fresh triterpenoid resins and aged triterpenoid varnishes by high-performance liquid chromatography–atmospheric pressure chemical ionisation (tandem) mass spectrometry. J. Chromatogr. A.

[46] Ancillotti C., Ulaszewska M., Mattivi F., Del Bubba M. (2018). Untargeted metabolomics analytical strategy based on liquid chromatography/electrospray ionization linear ion trap quadrupole/orbitrap mass spectrometry for discovering new polyphenol metabolites in human biofluids after acute ingestion of *vaccinium myrtillus* berry supplement. J. Am. Soc. Mass. Spectrom..

[47] Compaoré M., Meda R.N.-T., Bakasso S., Vlase L., Kiendrebeogo M. (2016). Antioxidative, anti-inflammatory potentials and phytochemical profile of *Commiphora africana* (A. Rich.) Engl. (Burseraceae) and *Loeseneriella africana* (Willd.) (Celastraceae) stem leaves extracts. Asian Pac. J. Trop. Biomed..

[48] Farid M.M., Aboul Naser A.F., Salem M.M., Ahmed Y.R., Emam M., Hamed M.A. (2022). Chemical compositions of *Commiphora opobalsamum* stem bark to alleviate liver complications in streptozotocin-induced diabetes in rats: Role of oxidative stress and DNA damage. Biomarkers.

[49] Yahagi T., Yamashita Y., Daikonnya A., Wu J.-b., Kitanaka S. (2010). New feruloyl tyramine glycosides from *Stephania hispidula* Y AMAMOTO. Chem. Pharm. Bull..

[50] Elsharabasy F.S., Hosney A.M. (2013). Chemical constituents from the aerial parts of *Salsola inermis*. Egypt. Pharm. J..

[51] Wang Z., Wang Q., Zhang M., Hu X., Ding G., Jiang M., Bai G. (2017). Cimicifugamide from *Cimicifuga rhizomes* functions as a nonselective β-AR agonist for cardiac and sudorific effects. Biomed. Pharmacother..

[52] ElNaggar M.H., Abdelmohsen U.R., Bar F.M.A., Kamer A.A., Bringmann G., Elekhnawy E. (2024). Investigation of bioactive components responsible for the antibacterial and anti-biofilm activities of *Caroxylon volkensii* by LC-QTOF-MS/MS analysis and molecular docking. RSC Adv..

[53] Jiang C., Gates P. (2024). Systematic characterisation of the fragmentation of flavonoids using high-resolution accurate mass electrospray tandem mass spectrometry. Molecules.

[54] Ahmad Z., Mehmood S., Fatima I., Malik A., Ifzal R., Afza N., Iqbal L., Latif M., Nizami T.A. (2008). Structural determination of salsolins A and B, new antioxidant polyoxygenated triterpenes from *Salsola baryosma*, by 1D and 2D NMR spectroscopy. Magn. Reson. Chem..

[55] Ahmad Z., Mehmood S., Ifzal R., Malik A., Afza N., Rashid F., Mahmood A., Iqbal L. (2007). Butyrylcholinesterase inhibitory triterpenes from *Salsola baryosma*. Pol. J. Chem..

[56] Hegazi N.M., El Shabrawy M., Aboulthana W.M., Ragheb A.Y., Ibrahim L.F., Marzouk M.M. (2025). UPLC-HRMS/MS Guided Isolation and NMR Investigation of Major Flavonoids from *Enarthrocarpus strangulatus* Boissier (Brassicaceae) with In Vitro Enzymes Inhibitory Potential. Chem. Biodivers..

[57] Xiong J.-L., Ma N. (2022). Transcriptomic and metabolomic analyses reveal that fullerol improves drought tolerance in *Brassica napus* L.. Int. J. Mol. Sci..

[58] Jahangir M., Kim H.K., Choi Y.H., Verpoorte R. (2009). Health-affecting compounds in Brassicaceae. Compr. Rev. Food Sci. Food Saf..

[59] Chen Y.-Y., Lu H.-Q., Jiang K.-X., Wang Y.-R., Wang Y.-P., Jiang J.-J. (2022). The flavonoid biosynthesis and regulation in *Brassica napus*: A review. Int. J. Mol. Sci..

[60] Manshood M.A., Al-Halbosiy M.M., Radhwan M.M. (2019). In vitro cytotoxic activity of some wild plants extracts against RAW264. 7 cell line. Plant Arch..

[61] Al-Mazroa S., Al-Wahaibi L., Mousa A., Al-Khathlan H. (2015). Essential oil of some seasonal flowering plants grown in Saudi Arabia. Arab. J. Chem..

[62] Ahmed N.Z., Ahmed-Farid O.A. (2017). Assessment of in-vitro and in vivo bioactivities potential of some Egyptian wild plants as anti-ulcerogenic effect on male albino rats. Pharm. Chem. J..

[63] Janbaz K., Aslam N., Imran I., Jabeen Q. (2021). Evaluation of anti-inflammatory, analgesic and antipyretic activities of *Salsola imbricata* Forssk in rats. J. Anim. Plant Sci..

[64] Elwekeel A., Hassan M.H., Almutairi E., AlHammad M., Alwhbi F., Abdel-Bakky M.S., Amin E., Mohamed E.I. (2023). Anti-inflammatory, anti-oxidant, GC-MS profiling and molecular docking analyses of non-polar extracts from five *Salsola* species. Separations.

[65] Alghamdi Y.S., Saleh O.M., Alqadri N., Mashraqi M.M., Bahattab O., Awad N.S. (2021). Effect of *Ducrosia flabellifolia* and *Savignya parviflora* extracts on inhibition of human colon and prostate cancer cell lines. Curr. Issues Mol. Biol..

[66] Al-Khayri J.M., Sahana G.R., Nagella P., Joseph B.V., Alessa F.M., Al-Mssallem M.Q. (2022). Flavonoids as Potential Anti-Inflammatory Molecules: A Review. Molecules.

[67] Kim H.P., Son K.H., Chang H.W., Kang S.S. (2004). Anti-inflammatory plant flavonoids and cellular action mechanisms. J. Pharmacol. Sci..

[68] Rathee P., Chaudhary H., Rathee S., Rathee D., Kumar V., Kohli K. (2009). Mechanism of action of flavonoids as anti-inflammatory agents: A review. Inflamm. Allergy Drug Targets.

[69] Abdel Bar F.M., Alonazi R., Elekhnawy E., Samra R.M., Alqarni M.H., Badreldin H., Magdy G. (2025). HPLC-PDA and in vivo anti-inflammatory potential of isorhamnetin-3-*O*-β-D-glucoside from *Zygophyllum simplex* L.. J. Ethnopharmacol..

[70] Choy K.W., Murugan D., Leong X.F., Abas R., Alias A., Mustafa M.R. (2019). Flavonoids as Natural Anti-Inflammatory Agents Targeting Nuclear Factor-Kappa B (NFκB) Signaling in Cardiovascular Diseases: A Mini Review. Front. Pharmacol..

[71] Ferraz C.R., Carvalho T.T., Manchope M.F., Artero N.A., Rasquel-Oliveira F.S., Fattori V., Casagrande R., Verri W.A. (2020). Therapeutic Potential of Flavonoids in Pain and Inflammation: Mechanisms of Action, Pre-Clinical and Clinical Data, and Pharmaceutical Development. Molecules.

[72] Dong X., Li J.-J., Ma N., Liu A.-Z., Liu J.-W. (2022). Anti-inflammatory and anti-oxidative effects of isorhamnetin for protection against lung injury in a rat model of heatstroke in a dry-heat environment. Med. Sci. Monit..

[73] Rana J.N., Gul K., Mumtaz S. (2025). Isorhamnetin: Reviewing Recent Developments in Anticancer Mechanisms and Nanoformulation-Driven Delivery. Int. J. Mol. Sci..

[74] Han Y., Yuan C., Zhou X., Han Y., He Y., Ouyang J., Zhou W., Wang Z., Wang H., Li G. (2021). Anti-Inflammatory Activity of Three Triterpene from Hippophae rhamnoides L. in Lipopolysaccharide-Stimulated RAW264.7 Cells. Int. J. Mol. Sci..

[75] Apaza Ticona L., Sánchez Sánchez-Corral J., Montoto Lozano N., Prieto Ramos P., Sánchez Á.R. (2024). Study of Pentacyclic Triterpenes from *Lyophilised Aguaje*: Anti-Inflammatory and Antioxidant Properties. Int. J. Mol. Sci..

[76] Bao T.R., Long G.Q., Wang Y., Wang Q., Liu X.L., Hu G.S., Gao X.X., Wang A.H., Jia J.M. (2022). New Lanostane-Type Triterpenes with Anti-Inflammatory Activity from the Epidermis of Wolfiporia cocos. J. Agric. Food Chem..

[77] Jiang Y., Yu L., Wang M.-H. (2015). N-trans-feruloyltyramine inhibits LPS-induced NO and PGE2 production in RAW 264.7 macrophages: Involvement of AP-1 and MAP kinase signalling pathways. Chem. Biol. Interact..

[78] Habib A., Bernard C., Lebret M., Creminon C., Esposito B., Tedgui A., Maclouf J. (1997). Regulation of the expression of cyclooxygenase-2 by nitric oxide in rat peritoneal macrophages. J. Immunol..

[79] Xia Z., Xu T.-Q., Xu W., Zhang H.-X., Liang Q., Zhou G.-X. (2019). Lyciyunin, a new dimer of feruloyltyramine and five bioactive tyramines from the root of *Lycium yunnanense* Kuang. Nat. Prod. Res..

[80] Cuiying Q., Feng C.-R., He Z.-Y., Wang Z., Qin C., Wang Q., Guo J., Yang B., Zang L. (2021). Study on the Mechanism of Erucamide Mediating Anti-stress Liver Injury in Rats by Inhibiting Glycine Cleavage. Res. Sq..

[81] Xie Y., Peng Q., Ji Y., Xie A., Yang L., Mu S.-Z., Li Z., He T., Xiao Y., Zhao J. (2021). Isolation and Identification of Antibacterial Bioactive Compounds From Bacillus megaterium L2. Front. Microbiol..

[82] Miao P., Wang H., Wang W., Wang Z., Ke H., Cheng H.-Y., Ni J.-J., Liang J., Yao Y.-F., Wang J. (2025). A widespread plant defense compound disarms bacterial type III injectisome assembly. Science.

[83] Dai J., Mumper R.J. (2010). Plant phenolics: Extraction, analysis and their antioxidant and anticancer properties. Molecules.

[84] Shadid K.A., Shakya A.K., Naik R.R., Al-Qaisi T.S., Oriquat G.A., Atoom A.M., Farah H.S. (2023). Exploring the Chemical Constituents, Antioxidant, Xanthine Oxidase and COX Inhibitory Activity of *Commiphora gileadensis* Commonly Grown Wild in Saudi Arabia. Molecules.

[85] Ohto U., Fukase K., Miyake K., Shimizu T. (2012). Structural basis of species-specific endotoxin sensing by innate immune receptor TLR4/MD-2. Proc. Natl. Acad. Sci. USA.

[86] Park B.S., Song D.H., Kim H.M., Choi B.S., Lee H., Lee J.O. (2009). The structural basis of lipopolysaccharide recognition by the TLR4-MD-2 complex. Nature.

[87] Kim H.M., Park B.S., Kim J.I., Kim S.E., Lee J., Oh S.C., Enkhbayar P., Matsushima N., Lee H., Yoo O.J. (2007). Crystal structure of the TLR4-MD-2 complex with bound endotoxin antagonist Eritoran. Cell.

[88] Duggan K.C., Walters M.J., Musee J., Harp J.M., Kiefer J.R., Oates J.A., Marnett L.J. (2010). Molecular basis for cyclooxygenase inhibition by the non-steroidal anti-inflammatory drug naproxen. J. Biol. Chem..

[89] Gupta K., Selinsky B.S., Kaub C.J., Katz A.K., Loll P.J. (2004). The 2.0 Å resolution crystal structure of prostaglandin H2 synthase-1: Structural insights into an unusual peroxidase. J. Mol. Biol..

[90] Attard E. (2013). A rapid microtitre plate Folin-Ciocalteu method for the assessment of polyphenols. Cent. Eur. J. Biol..

[91] Kiranmai M., Kumar C.B., Ibrahim M. (2011). Comparison of total flavanoid content of *Azadirachta indica* root bark extracts prepared by different methods of extraction. Res. J. Pharm. Biol. Chem. Sci..

[92] Rafeeq M., Murad H.A.S., Abdallah H.M., El-Halawany A.M. (2021). Protective effect of 6-paradol in acetic acid-induced ulcerative colitis in rats. BMC Complement. Med. Ther..

[93] Ince S., Ozer M., Kadioğlu B.G., Kuzucu M., Yilmaz S.K., Özkaraca M., Gezer A., Suleyman H. (2021). The effect of adenosine triphosphate on bevacizumab-induced ovarian damage and reproductive dysfunction in rats. Gen. Physiol. Biophys..

[94] Labib D.A.A., Shaker O.G., Elfarouk L.O. (2016). Protective effects of nebivolol on acetic acid-induced ulcerative colitis in rats. Kasr Al Ainy Med. J..

[95] Ahmed H.A., Fahmy E.M., Abdelkreem E., Mahmoud E.A., Nafady A., Ahmed E.H., Cancer (2023). Frequency of toll-like receptor 4 variants and association with treatment response in children with primary immune thrombocytopenia. Pediatr. Blood Cancer.

[96] Lowry O., Rosebrough N., Farr A.L., Randall R. (1951). Protein measurement with the Folin phenol reagent. J. Biol. Chem..

[97] Culling C.F.A. (2013). Handbook of Histopathological and Histochemical Techniques: Including Museum Techniques.

[98] Trott O., Olson A.J. (2010). AutoDock Vina: Improving the speed and accuracy of docking with a new scoring function, efficient optimization, and multithreading. J. Comput. Chem..

[99] O’Boyle N.M., Banck M., James C.A., Morley C., Vandermeersch T., Hutchison G.R. (2011). Open Babel: An open chemical toolbox. J. Cheminform..

[100] BIOVIA D.S. (2021). Discovery Studio Visualizer.

[101] Lee J.-T., Pao L.-H., Hsieh C.-D., Huang P.-W., Hu O.Y.-P. (2017). Development and validation of an LC-MS/MS method for simultaneous quantification of hesperidin and hesperetin in rat plasma for pharmacokinetic studies. Anal. Methods.

[102] Emir A., Emir C., Yıldırım H. (2020). Characterization of phenolic profile by LC-ESI-MS/MS and enzyme inhibitory activities of two wild edible garlic: *Allium nigrum* L. and *Allium subhirsutum* L. J. Food Biochem..

[103] Wood K.V., Bonham C.C., Miles D., Rothwell A.P., Peel G., Wood B.C., Rhodes D. (2002). Characterization of betaines using electrospray MS/MS. Phytochemistry.

[104] Rozenberg R., Ruibal-Mendieta N.L., Petitjean G., Cani P., Delacroix D.L., Delzenne N.M., Meurens M., Quetin-Leclercq J., Habib-Jiwan J.-L. (2003). Phytosterol analysis and characterization in spelt (*Triticum aestivum* ssp. *spelta* L.) and wheat (*T. aestivum* L.) lipids by LC/APCI-MS. J. Cereal Sci..

[105] Xiang Y., Li Y.B., Zhang J., Li P., Yao Y.Z. (2007). Studies on chemical constituents of *Salsola collina*. Zhongguo Zhong Yao Za Zhi.

[106] Park C.H., Bong S.J., Lim C.J., Kim J.K., Park S.U. (2020). Transcriptome analysis and metabolic profiling of green and red mizuna (*Brassica rapa* L. var. *japonica*). Foods.

[107] Tomas M., Zhang L., Zengin G., Rocchetti G., Capanoglu E., Lucini L. (2021). Metabolomic insight into the profile, in vitro bioaccessibility and bioactive properties of polyphenols and glucosinolates from four Brassicaceae microgreens. Food Res. Int..

[108] Ressurreição S., Salgueiro L., Figueirinha A. (2025). *Diplotaxis muralis* as an emerging food crop: Chemical composition, nutritional profile and antioxidant activities. Plants.

[109] Lucarini M., Di Cocco M.E., Raguso V., Milanetti F., Durazzo A., Lombardi-Boccia G., Santini A., Delfini M., Sciubba F. (2020). NMR-based metabolomic comparison of *Brassica oleracea* (var. *italica*): Organic and conventional farming. Foods.

