# Supplementary material for: Chemical Profiling of Commiphora gileadensis, Salsola incanescens, and Savignya parviflora and Their Protective Effects in an Acute Rat Model of Ulcerative Colitis
Source: Pharmaceuticals (Basel). 2026 Jul 27;19(8):1178. doi: 10.3390/ph19081178 (PMC13516978; doi:10.3390/ph19081178)
Supplement: Supplementary file 1 [file pharmaceuticals-19-01178-s001.zip › pharmaceuticals-4403190-supplementary.pdf]

Supplementary materials

Article

Chemical Profiling of *Commiphora gileadensis*, *Salsola incanescens*, and *Savignya parviflora* and Their Protective Effects in an Acute Rat Model of Ulcerative Colitis

Fawaz K. Alanazi [1,†], Nashwa Hashad [1,†], Asmaa A. Ahmed [2], Elsayed K. El-Sayed [2], Yara E. Mansour [3,4], Mohamed I.S. Abdelhady [1], Eman G. Haggag [1,‡] and Fatma M. Abdel Bar [5,6,‡,*]

1 Department of Pharmacognosy, Faculty of Pharmacy, Capital University (Formerly Helwan University), Cairo 11795, Egypt; ph.fawaz@hotmail.com (F.K.A.); nashwa.elsayed@pharm.capu.edu.eg (N.H.); mohamed_abdelhady@pharm.capu.edu.eg (M.I.S.A.); eman.g.haggag@pharm.capu.edu.eg (E.G.H.)
2 Department of Pharmacology and Toxicology, Faculty of Pharmacy, Capital University (Formerly Helwan University), Cairo 11795, Egypt; dr.asmaa_ali@pharm.capu.edu.eg (A.A.A); elsayed_elsayed@pharm.capu.edu.eg (E.K.E.)
3 Department of Pharmaceutical Chemistry and Pharmacognosy, College of Pharmacy, Qassim University, Unaizah 51911, Saudi Arabia; y.mansour@qu.edu.sa (Y.E.M.)
4 Pharmaceutical Organic Chemistry Department, Faculty of Pharmacy, Capital University (Formerly Helwan University), Cairo 11795, Egypt
5 Department of Pharmacognosy, College of Pharmacy, Prince Sattam Bin Abdulaziz University, Al-Kharj 11942, Saudi Arabia; f.abdelbar@psau.edu.sa (F.M.A)
6 Department of Pharmacognosy, Faculty of Pharmacy, Mansoura University, Mansoura 35516, Egypt
* Correspondence: ; f.abdelbar@psau.edu.sa
† These authors share equal contributions of the first authorship
‡ These authors share equal contributions of the last authorship

Table of Contents	Page
**Table S1.** Phytochemical compounds identified in the total extract of *C. gileadensis* by LC-/MS in positive and negative ionization modes.	3
**Table S2.** Phytochemical compounds identified in the total extract of *S. incanescens* by LC-MS/MS in positive and negative ionization modes.	8
**Table S3.** Phytochemical compounds identified in the total extract of *S. parviflora* by LC-MS/MS in positive and negative ionization modes.	15
**Figure S1.** Spectrum of compound **1.1.3** (TOF MS^2) for the precursor ion (R_t 6.918 min) at *m/z* 481.2572; gossypin.	20
**Figure S2.** Spectrum of compound **1.1.13** (TOF MS^2) for the precursor ion (R_t 8.879 min) at *m/z* 611.2037; neohesperidin dihydrochalcone.	20
**Figure S3.** Spectrum of compound **1.1.21** (TOF MS^2) for the precursor ion (R_t 10.26 min) at *m/z* 447.0986; isorhamnetin pentoside.	20
**Figure S4.** Spectrum of compound **1.1.17** (TOF MS^2) for the precursor ion (R_t 9.388 min) at *m/z* 431.1047; Kaempferol-3-*O*-alpha-L-rhamnoside.	21
**Figure S5.** Spectrum of compound **1.1.10** (TOF MS^2) for the precursor ion (R_t 7.674 min) at *m/z* 447.1019; quercitrin.	21
**Figure S6.** Spectrum of compound **1.1.4** (TOF MS^2) for the precursor ion (R_t 7.311 min) at *m/z* 609.1585; hesperidin/ neohesperidin.	21
**Figure S7.** Proposed MS^2 fragmentation of commigileadin A.	22
**Figure S8.** Proposed MS^2 fragmentation of canophyllal.	22
**Figure S9.** Spectrum of compound **1.2.1** (TOF MS^2 negative mode) for the precursor ion (R_t 4.73 min) at *m/z* 353.0942; chlorogenic acid.	22
**Figure S10.** Spectrum of compound **1.2.1** (TOF MS^2 positive mode) for the precursor ion (R_t 4.71 min) at *m/z* 355.1024; chlorogenic acid.	23

**Figure S11.** Spectrum of compound **1.2.4** (TOF MS^2) for the precursor ion (R_t 9.587 min) at *m/z* 207.0752; sinapyl aldehyde.	23
**Figure S12.** Spectrum of compound **1.2.5** (TOF MS^2) for the precursor ion (R_t 7.502 min) at *m/z* 163.0435; 3-(4-hydroxyphenyl) prop-2-enoic acid (*p*-coumaric acid)	23
**Figure S13.** Spectrum of compound **1.4.1** (TOF MS^2) for the precursor ion (R_t 9.497 min) at *m/z* 405.1726; *E*-3,4,5'-trihydroxy-3'-glucopyranosylstilbene.	24
**Figure S14.** Spectrum of compound **1.6.1** (TOF MS^2) for the precursor ion (R_t 17.471 min) at *m/z* 293.2113; 12-oxo-10,15(Z)-phytodienoic acid.	24
**TOF MS^2 spectra of selected compounds identified from *S. incanescens* aerial parts AME in positive or negative ionization modes.**	24
**Figure S15.** Spectrum of compound **2.1.7** (TOF MS^2) for the precursor ion (R_t 7.7287 min) at *m/z* 476.1903; *trans-N*-feruloyl tyramine-4```-*O*-*β*-D-glucopyranoside.	24
**Figure S16.** Spectrum of compound **2.1.8** (TOF MS^2) for the precursor ion (R_t 7.8222 min) at *m/z* 506.2007; cimicifugamide; *N*-(*trans*-4-*O*-*β*-D-glucopyranoside feruloyl)-3`-*O*-methyldopamine.	25
**Figure S17.** Spectrum of compound **2.1.13** (TOF MS^2) for the precursor ion (R_t 9.586783 min) at *m/z* 344.1492; *N-trans*-feruloyl-3-*O*-methyldopamine; *N-trans*-feruloyl-4`-*O*-methyldopamine.	25
**Figure S18.** Spectrum of compound **2.1.9** (TOF MS^2) for the precursor ion (R_t 8.24435 min) at *m/z* 360.1436; 2`-hydroxy- 3``-methyoxymoupinamide; (*E*)-*N*-(2-hydroxy-2-(3-hydroxy-4-methoxyphenyl)ethyl)-3-(4-hydroxy-3-methoxyphenyl) acrylamide.	25
**Figure S19.** Spectrum of compound **2.1.4** (TOF MS^2) for the precursor ion (R_t 6.674 min) at *m/z* 360.1432; *N*-(3`,4`-dimethoxy-cinnamoyl)-norepinephrine.	26
**TOF MS^2 spectra of selected compounds identified from *S. parviflora* aerial parts AME in positive or negative ionization modes.**	26
**Figure S20.** Spectrum of compound **3.7.2** (TOF MS^2) for the precursor ion (R_t 26.06min) at *m/z* 338.3408; erucamide.	26
**Figure S21.** Spectrum of compound **3.7.1** (TOF MS^2) for the precursor ion (R_t 9.71min) at *m/z* 314.1375; feruloyltyramine.	26
**Figure S22.** Calibration curve of gallic acid for determination of total phenolic content in the extracts. Y-axis represents the absorbance and X-axis represents gallic acid concentration in μg/mL.	27
**Figure S23.** Calibration curve of quercetin for determination of total flavonoid content in the extracts. Y-axis represents the absorbance and X-axis represents quercetin concentration in μg/mL.	27
**Table S4.** TPC of the methanol extracts of the aerial parts of *C. gileadensis*, *S. incanescens*, and *S. parviflora.*	27
**Table S5.** TFC of the methanol extracts of the aerial parts of *C. gileadensis*, *S. incanescens*, and *S. parviflora.*	27
**Figure S24.** 2D (right) and 3D (left) visualization structure interaction poses of TLR4/MD-2 active site (PDB ID: 2Z65) with selected key identified compounds in the three investigated extracts.	32
**Table S6.** Results of molecular docking of the crystallized ligand and selected key bioactive compounds from investigated plants in conjunction with TLR4 (PDBID: 2Z65).	33
**Figure S25.** 2D (right) and 3D (left) visualization structure interaction poses of COX-1 (PDB: 1Q4G) with selected key identified compounds in the three investigated extracts.	43
**Figure S26.** 2D (right) and 3D (left) visualization structure interaction poses of COX-2 (PDB: 3NT1) with selected key identified compounds in the three investigated extracts.	48
**References**	48

Table S1. Phytochemical compounds identified in *C. gileadensis* the methanolic extracts of the aerial parts (AME) by LC-MS/MS in positive and negative ionization modes.

No.	Identified compounds	R_t (min)	Precursor (*m/z*)	Reference (*m/z*)	Formula	Error (ppm)	Mode	MS² spectrum	Confidence level [1]	References
**1.1. Flavonoids**										
**1.1.1**	Acacetin-7-*O*-rutinoside (Linarin)	5.91	591.163	591.17194	$C_{28}H_{32}O_{14}$	-15.1	-ve	591	Tentative	[1]
**1.1.2**	Okanin-4'-*O*-glucoside	6.51	449.113	449.10895	$C_{21}H_{22}O_{11}$	9.0	-ve	449	Tentative	[1]
**1.1.3**	Gossypin	6.92	481.0960	481.09766	$C_{21}H_{20}O_{13}$	–3.45	+ve	169, 319, 481	Probable	MSBNK-RIKEN-PR100353[2], (Figure S1)
**1.1.4**	Hesperidin/ Neohesperidin	7.31	609.1549	609.1825	$C_{28}H_{34}O_{15}$	+6.57	-ve	301, 302, 607, 609	Probable	MSBNK-ACES_SU-AS001489 MSBNK-BS-BS003651 [2] (Figure S6)
**1.1.5**	Apigenin 8-*C*-glucoside	7.46	433.1127	433.11292	$C_{21}H_{20}O_{10}$	-0.5	+ve	313, 343	Tentative	[3]
**1.1.6**	Delphinidin-3-*O*-beta-glucopyranoside	7.54	463.0931	463.08765	$C_{21}H_{21}O_{12}$	11.8	-ve	165, 301, 302	Tentative	[4]
**1.1.7**	Hyperoside	7.56	465.1018	465.10275	$C_{21}H_{20}O_{12}$	-2.0	+ve	91, 145, 303, 305	Probable	MSBNK-Fiocruz-FIO00151 [2]
**1.1.8**	Kaempferol-3-glucuronide	7.60	461.0821	461.07254	$C_{21}H_{18}O_{12}$	+20.7	-ve	299, 415, 461	Tentative	[5]
**1.1.9**	Luteolin-4'-*O*-glucoside	7.66	449.1074	449.10785	$C_{21}H_{20}O_{11}$	-1.0	+ve	287, 288, 449, 450	Probable	MSBNK-IPB_Halle-PN000062 [2]
		7.63	447.0965	447.09329	$C_{21}H_{20}O_{11}$	7.2	-ve	284, 285	Probable	[5]
**1.1.10**	Quercitrin	7.67	447.1019	447.09329	$C_{21}H_{20}O_{11}$	+10.5	-ve	284, 285, 447	Tentative	[1] (Figure S5)
**1.1.11**	(+)-Taxifolin	8.08	303.0534	303.05103	$C_{15}H_{12}O_7$	7.8	-ve	285, 125	Tentative	[6]
**1.1.12**	Isorhamnetin-3-*O*-glucoside	8.19	477.108	477.10385	$C_{22}H_{22}O_{12}$	8.7	-ve	285, 299, 314	Tentative	[5]
**1.1.13**	Neohesperidin dihydrochalcone	8.88	611.1856	611.19812	$C_{28}H_{36}O_{15}$	–20.5	-ve	447, 611	Tentative	[1], MSBNK-BS-BS003658[2]

										(Figure S2)
**1.1.14**	Myricetin	8.90	317.0327	317.0303	$C_{15}H_{10}O_8$	7.6	-ve	165, 317	Tentative	[4]
**1.1.15**	Isorhamnetin-3-*O*-rutinoside	9.01	623.1683	623.16174	$C_{28}H_{32}O_{16}$	10.5	-ve	300, 315	Tentative	[5]
		9.01	625.1736	625.17633		-4.4	+ve	317, 479	Tentative	[7]
**1.1.16**	Kaempferol-3-*O*-(6-p-coumaroyl)-glucoside	9.10	593.1356	593.13007	$C_{30}H_{26}O_{13}$	9.3	-ve	447, 593	Probable	MSBNK-RIKEN-PR100819
**1.1.17**	Kaempferol-3-*O*-alpha-L-rhamnoside	9.39	431.1023	431.09836	$C_{21}H_{20}O_{10}$	+9.1	-ve	285, 431	Probable	[5], MSBNK-RIKEN-PR100970 [2] (Figure S4)
**1.1.18**	3', 4', 5, 7-tetrahydroxyflavanone (eriodictoyl)	10.04	287.0588	287.05612	$C_{15}H_{12}O_6$	9.3	-ve	135, 151, 229, 241, 255, 256, 257, 285, 283, 284, 285, 287	Probable	MSBNK-BS-BS003464[2]
**1.1.19**	Luteolin	10.12	285.0425	285.04047	$C_{15}H_{10}O_6$	7.1	-ve	133, 151, 175, 199, 285, 286	Probable	[8], MSBNK-BS-BS003099[2]
		10.12	287.0551	287.05502		0.28	+ve	153, 287	Probable	MSBNK-BGC_Munich-RP004802 [2]
**1.1.20**	Quercetin	10.18	301.0371	301.03537	$C_{15}H_{10}O_7$	5.75	-ve	65, 107, 121, 151, 179, 273, 301	Probable	[1], MSBNK-BAFG-CSL2311091866[2]
		10.20	303.0495	303.04993		1.42	+ve	153, 165, 201, 229, 303	Probable	MSBNK-BGC_Munich-RP012402[2]
**1.1.21**	Isorhamnetin pentoside	10.26	447.0928	447.09329	$C_{21}H_{20}O_{11}$	–1.10	-ve	447, 314, 315, 327, 299, 285	Tentative	[9], **(**Figure S3**)**
**1.1.22**	Apigenin	11.20	271.0601	271.06009	$C_{15}H_{10}O_5$	0.04	+ve	153, 271	Probable	MSBNK-BGC_Munich-RP015102[2]
**1.1.23**	Naringenin	11.23	271.0633	271.06119	$C_{15}H_{12}O_5$	7.8	-ve	119, 125, 151, 177, 253, 271	Probable	[4]
		11.23	273.0753	273.07574		-1.6	+ve	273	Probable	MSBNK-Washington_State_Univ-BML81780 [2]
**1.1.24**	Hesperetin	11.64	301.0738	301.07175	$C_{16}H_{14}O_6$	6.8	-ve	164	Tentative	[10]
**1.1.25**		11.67	315.0544	315.0510	$C_{16}H_{12}O_7$	8.79	-ve	315, 300	Probable	[5]

	3'-Methoxy-4',5,7-Trihydroxyflavonol (isorhamnetin)	11.67	317.0653	317.06558		-0.9	+ve	55, 67, 69, 77, 79, 81, 91, 109, 121, 123, 124, 128, 137, 142,149, 151, 153, 158, 217, 229, 245, 273, 287, 301, 302,316, 317	Probable	MSBNK-Fac_Eng_Univ_Tokyo-JP000694[2]
**1.1.26**	3, 5, 7-trihydroxy-4'-methoxyflavone (Kaempferide)	14.19	299.0586	299.05612	$C_{16}H_{12}O_6$	8.3	-ve	165, 243, 271, 299	Probable	[1]
		11.49	301.0714	301.07068	$C_{16}H_{12}O_6$	2.4	+ve	258, 286, 301	Probable	[7], MSBNK-RIKEN-PR040032 [2]
**1.1.27**	Acacetin	14.03	283.063	283.06119	$C_{16}H_{12}O_5$	6.4	-ve	268	Tentative	[6]
**1.2. Simple phenolic derivatives**										
**1.2.1**	Chlorogenic acid	4.73	353.091	353.0878	$C_{16}H_{18}O_9$	9.1	-ve	117, 135, 145, 163	Probable	[1] MSBNK-Fiocruz-FIO00627 [2], (Figure S9)
		4.71	355.1020	355.10236		–1.0	+ve	89, 117, 135, 145, 162, 163	Probable	MSBNK-Fiocruz-FIO00622 [2], (Figure S10)
**1.2.2**	Vanillic acid glucoside	4.99	329.0906	329.0878	$C_{14}H_{18}O_9$	8.5	-ve	167	Tentative	[11]
**1.2.3**	3-(4-Hydroxy-3,5-dimethoxyphenyl)-2-propenoic acid (sinapic acid)	6.37	223.0658	223.0612	$C_{11}H_{12}O_5$	20.6	-ve	79, 96, 224	Tentative	MSBNK-BGC_Munich-RP017402 [2]
**1.2.4**	Sinapyl aldehyde	9.59	207.0698	207.06628	$C_{11}H_{12}O_4$	-0.9	-ve	79, 96, 162.9, 192, 207	Probable	[1], MSBNK-Osaka_Univ-OUF00460 [2], (Figure S11)
**1.2.5**	3-(4-Hydroxyphenyl) prop-2-enoic acid (*p*-coumaric acid)	7.50	163.0431	163.0401	$C_9H_8O_3$	+18.4	-ve	93, 117, 119, 163	Tentative	[12], MSBNK-BGC_Munich-RP016811[2], (Figure S12)
**1.3. Coumarins**										
**1.3.1**	6,7-Dihydroxycoumarin (Cichorigenin, Esculetin)	6.36	177.0203	177.01933	$C_9H_6O_4$	5.5	-ve	65, 67, 68, 77, 79, 81, 89, 89, 91, 93, 105, 107, 109, 121, 133,149, 177	Probable	MSBNK-RIKEN-PR100939 [2]

**1.3.2**	Daphnetin	8.08	177.0186	177.01933	$C_9H_6O_4$	-4.1	-ve	121	Tentative	[5]
**1.3.3**	Scopoletin	14.60	193.0493	193.04953	$C_{10}H_8O_4$	-1.2	+ve	134, 137, 149, 150, 161, 165,178, 193	Probable	MSBNK-Fiocruz-FIO00949 [2]
**1.4. Stilbenes**										
**1.4.1**	*E*-3,4,5'-Trihydroxy-3'-glucopyranosylstilbene	9.50	405.1256	405.11911	$C_{20}H_{22}O_9$	+16.0	-ve	243, 405	Tentative	[1], MSBNK-RIKEN_ReSpect-PS122909 [2] (Figure S13)
**1.5. carboxylic acids**										
**1.5.1**	D-(-)-Quinic acid	1.26	191.0575	191.05611	$C_7H_{12}O_6$	7.3	-ve	58, 85, 87, 93, 109, 111, 127, 137, 171,173, 190, 191	Tentative	[1]
**1.5.2**	Maleic acid	1.32	115.0042	115.00368	$C_4H_4O_4$	4.5	-ve	71	Tentative	[5]
**1.5.3**	3,4-Dihydroxybenzoic acid	4.70	153.0203	153.01933	$C_7H_6O_4$	6.3	-ve	109, 153	Tentative	[1]
**1.5.4**	3,4-Dihydroxyphenylacetic acid	6.42	167.0369	167.03499	$C_8H_8O_4$	11.4	-ve	123	Tentative	MSBNK-RIKEN_ReSpect-PS046607[2]
**1.6. Fatty acids and fatty acid derivatives:**										
**1.6.1**	12-Oxo-10,15(Z)-phytodienoic acid	17.47	293.2108	293.21112	$C_{18}H_{28}O_3$	–1.1	+ve	79, 107, 275, 293	Probable	MSBNK-RIKEN-PR310790 [2], (Figure S14)
**1.6.2**	Suberic acid	7.55	173.0845	173.08194	$C_8H_{14}O_4$	+14.8	-ve	83, 111, 173	Tentative	[1], MSBNK-BGC_Munich-RP015311 [2]
**1.6.3**	*gamma*-Linolenic acid	17.77	277.219	277.21732	$C_{18}H_{30}O_2$	6.1	-ve	277, 233	Probable	MSBNK-Antwerp_Univ-METOX_N104928_B8BB [2]
**1.6.4**	(9Z, 12Z)-Octadecadienoate; Linoleic acid	22.10	279.2355	279.2330	$C_{18}H_{32}O_2$	+8.96	-ve	261, 277, 279, 280	Probable	MSBNK-Antwerp_Univ-METOX_N105527_9CB7 [2]
**1.7. Amino acid derivatives:**										
**1.7.1**	Glycine-Betaine	1.22	118.0861	118.08626	$C_5H_{11}NO_2$	-1.4	+ve	58, 59	Tentative	[13]
**1.7.2**	L-Proline	1.26	116.0702	116.0706	$C_5H_9NO_2$	-3.4	+ve	53, 68, 70, 116	Probable	MSBNK-Antwerp_Univ-METOX_P100401_EF88 [2]

**1.7.3**	S-Adenosyl-L-methionine	9.03	399.1405	399.1445	$C_{15}H_{22}N_6O_5S$	-10.0	+ve	135, 162, 399	Probable	MSBNK-Keio_Univ-KO002074 [2]
**1.8. Sugars**										
**1.8.1**	D-(+)-Trehalose	12.10	341.1071	341.10895	$C_{12}H_{22}O_{11}$	-5.4	-ve	121, 205	Tentative	[5]
**1.9. Triterpenes and sterols**										
**1.9.1**	Oleanonic aldehyde	17.39	439.3563	439.3571	$C_{30}H_{46}O_2$	-1.8	+ve	393,421,439	Tentative	[14]
**1.9.2**	11-Oxo-oleanonic acid	19.42	469.3307	469.3312	$C_{30}H_{44}O_4$	-1.07	+ve	423, 451, 469	Tentative	[14]
**1.9.3**	11-Oxo-ursonic acid	20.15	469.3307	469.3312	$C_{30}H_{44}O_4$	-1.07	+ve	423, 451, 469	Tentative	[14]
**1.9.4**	Oleanonic acid	21.03	455.3505	455.3520	$C_{30}H_{46}O_3$	-3.3	+ve	409, 437, 455	Tentative	[14]
**1.9.5**	Commigileadin A	22.53	455.351	455.3520	$C_{30}H_{46}O_3$	-2.2	+ve	455	Probable	[15] Proposed MS^2 fragmentation (Figure S7)
**1.9.6**	Urosonic acid	22.80	455.3505	455.3520	$C_{30}H_{46}O_3$	-3.3	+ve	409, 437, 455	Tentative	[14]
**1.9.7**	Canophyllal	23.07	441.3727	441.3723	$C_{30}H_{48}O_2$	-0.**91**	+ve	441	Probable	[16] Proposed MS^2 fragmentation (Figure S8)
**1.9.8**	Urosonic aldehyde	23.34	439.3561	439.3571	$C_{30}H_{46}O_2$	-2.3	+ve	393, 411., 421, 439	Tentative	[14]
**1.9.9**	Stigmasterol	22.52	413.3763	413.3778	$C_{29}H_{48}O$	-3.6	+ve	395	Tentative	[17]
**1.10. Miscellaneous**										
**1.10.1**	Zearalenone	8.42	317.1459	317.13943	$C_{18}H_{22}O_5$	0.3	-ve	299, 317	Probable	MSBNK-HBM4EU-HB002990 [2]
**1.10.2**	*all-trans*-Retinoic acid	17.61	301.2155	301.21622	$C_{20}H_{28}O_2$	-2.4	+ve	91, 105, 119, 123, 131, 133, 145, 159, 201, 301	Probable	MSBNK-RIKEN-PR100470 [2]

[1] Confidence levels were assigned according to the identification scale proposed by Schymanski et al. [18]. No **Standard** (Level 1: Confirmed structure) identifications were achieved, as authentic reference standards were not analyzed. **Probable** (Level 2) identifications were assigned based on unambiguous library spectral matches (Level 2a) or structural rationales supported by diagnostic MS2 fragmentation patterns (Level 2b). **Tentative/ putative** (Level 3) identifications denote candidates where evidence exists for a structural class or specific compound, but the assignment remains speculative due to high mass errors (> ±10 ppm) or unresolved structural features.

Table S2. Phytochemical compounds identified *S. incanescens* AME by LC-MS/MS in positive and negative ionization modes.

No.	Identified compounds	R_t (min)	Precursor (*m/z*)	Reference (*m/z*)	Formula	Error (ppm)	Mode	MS^2 spectrum	Confidence level [1]	References
**2.1. Alkaloids and nitrogenous compounds:**										
**2.1.1**	Vulgaxanthin I	1.31	340.122	340.1139	$C_{14}H_{17}N_3O_7$	-23.8	+ve	171, 85, 157, 175, 340	Tentative	[19]
**2.1.2**	Tyramine-*O*-*β*-D-glucoside	6.32	298.1323	298.1291	$C_{14}H_{21}NO_6$	10.73	-ve	298, 252, 121 (100), 77	Tentative	[19]
**2.1.3**	Salsolidine or *N*-Methylisosalsolidine	6.33	208.1324	208.1332	$C_{12}H_{17}NO_2$	3.8	+ve	208, 149, 105, 77, 53	Probable	[19,20]
**2.1.4**	*N*-(3`,4`-Dimethoxy-cinnamoyl)-norepinephrine	6.76	360.1432	360.1442	$C_{19}H_{21}NO_6$	2.8	+ve	117, 130, 145, 167, 177, 342, 360	Probable	(Figure S19)[19]
**2.1.5**	Phenaturic acid	6.81	192.0684	192.06662	$C_{10}H_{11}NO_3$	-0.9	-ve	74, 192	Probable	MSBNK Fac_Eng_Univ_Tokyo-JP002233 [2]
**2.1.6**	4```-*O*-β-D-Glucopyranosyl caffeoyltyramine	7.57	462.1739	462.1759	$C_{23}H_{27}NO_9$	4.3	+ve	118, 163, 300, 341, 445, 462	Probable	[19]
**2.1.7**	*Trans*-*N*-Feruloyl tyramine-4```-*O*-β-D-glucopyranoside	7.73	476.1903	476.1915	$C_{24}H_{29}NO_9$	2.5	+ve	476, 314 (100), 191, 177, 145, 121	Probable	Not reported before from the plant (Figure S15)[21-23]
**2.1.8**	Cimicifugamide; *N*-(*Trans*-4-*O*-β-D-glucopyranoside feruloyl)-3`-*O*-methyldopamine	7.82	506.2007	506.2021	$C_{25}H_{31}NO_{10}$	2.8	+ve	506, 314, 191, 177 (100), 145	Probable	Not reported before from the plant (Figure S16) [24]
**2.1.9**	2`-Hydroxy- 3``-methyoxymoupinamide; (*E*)-*N*-(2-hydroxy-2-(3-hydroxy-4-methoxyphenyl)ethyl)-3-(4-hydroxy-3-methoxyphenyl)acrylamide	8.24	360.1436	360.1442	$C_{19}H_{21}NO_6$	1.7	+ve	117, 145, 177 (100), 218, 342	Probable	(Figure S18) [19]
**2.1.10**	1*H*-indole-3-carboxylic acid	8.55	160.0414	160.04041	$C_9H_7NO_2$	1	-ve	116, 117, 160	Probable	MSBNK-RIKEN_ReSpect-PT110910 [2]

**2.1.11**	*N*-Caffeoyltyramine	8.62	300.1223	300.1230	$C_{17}H_{17}NO_4$	2.3	+ve	117, 121, 135, 163 (100), 300	Probable	[19]
**2.1.12**	*N-Trans*-feruloyl tyramine (Moupinamide)	9.39	314.1384	314.1387	$C_{18}H_{19}NO_4$	1.0	+ve	177, 145, 314, 121, 117	Probable	[19]
**2.1.13**	*N-Trans*-feruloyl-3-*O*-methyldopamine; *N-trans*-feruloyl-4`-*O*-methyldopamine	9.59	344.1492	344.1492	$C_{19}H_{21}NO_6$	0.0	+ve	177 (100), 145, 117, 344, 151	Probable	(Figure S17) [19]
**2.1.14**	4-Nitrophenol	9.69	138.0204	138.01967	$C_6H_5NO_3$	-0.9	-ve	92, 108, 138	Probable	MSBNK-LCSB-LU098252 [2]
**2.1.15**	Dopamine; 3-*O*-*β*-D-glucopyranoside / octopamine-*O*-*β*-D-glucoside	9.72	312.1279	312.1245	$C_{14}H_{21}NO_7$	10.89	-ve	312	Tentative	[19]
**2.1.16**	(*E*)-3-(4-Hydroxy-3-methoxyphenyl)-*N*-(4-methoxyphenethyl)acrylamide	10.36	328.1534	328.1543	$C_{19}H_{21}NO_4$	2.7	+ve	177, 328, 121, 145, 117	Probable	[19]
**2.1.17**	Erucamide	26.07	338.3414	338.3417	$C_{22}H_{43}NO$	0.9	+ve	338, 321, 303, 57, 69	Probable	[19]
**2.2. Flavonoids:**										
**2.2.1**	3 3' 4' 5 7-Pentahydroxyflavan; Epicatechin	1.24	289.0741	289.07175	$C_{15}H_{14}O_6$	-1.4	-ve	289	Probable	MSBNK-RIKEN-PR100688 MSBNK-RIKEN-PR100689 [2]
**2.2.2**	3'-Methoxy-4',5,7-trihydroxyflavonol; Isorhamnetin	1.31	315.134	315.05103	$C_{16}H_{12}O_7$	0.6	-ve	123, 146, 149, 151, 193, 211, 255, 271, 314, 314, 315	Probable	MSBNK-RIKEN-PR040022 [2]
**2.2.3**	Luteolin	4.52	285.0653	285.04047	$C_{15}H_{10}O_6$	-0.6	-ve	107, 133, 151, 165, 223, 241, 243, 267, 285	Probable	MSBNK-BS-BS003099 [2]
**2.2.4**	Kaempferol-3-*O*-alpha-L-arabinoside	4.97	417.1093	417.08273	$C_{20}H_{18}O_{10}$	-0.2	-ve	149, 151, 177, 193, 207, 285, 417	Probable	[25]
**2.2.5**	Kaempferol-3-*O*-alpha-L-rhamnoside	5.26	431.1259	431.09836	$C_{21}H_{20}O_{10}$	-0.8	-ve	151, 177, 207, 285, 287, 431	Probable	[25]
**2.2.6**	Apigenin	6.54	269.1093	269.04553	$C_{15}H_{10}O_5$	0.9	-ve	207, 269, 270	Probable	MSBNK-Fiocruz-FIO00012 [2]

**2.2.7**	Hesperidin	7.31	609.1559	609.1825	$C_{28}H_{34}O_{15}$	-1	-ve	151, 243, 255, 271, 300, 301, 609	Probable	MSBNK-Washington_State_Univ-BML00690 [2]
**2.2.8**	Daidzein-8-C-glucoside	7.67	417.1135	417.11801	$C_{21}H_{20}O_9$	-10.8	+ve	169, 220, 417	Tentative	MSBNK-RIKEN-PR302166 [2]
**2.2.9**	Isorhamnetin-3-*O*-rutinoside	7.94	623.17	623.16174	$C_{28}H_{32}O_{16}$	0.3	-ve	243, 271, 287, 299, 300, 313, 314, 315, 623, 624	Probable	MSBNK-RIKEN-PR040099 [2]
**2.2.10**	Syringetin-3-*O*-galactoside	8.17	507.2294	507.11441	$C_{23}H_{24}O_{13}$	0.1	-ve	93, 342, 342, 507	Probable	MSBNK-RIKEN-PR305757 [2]
**2.2.11**	Isorhamnetin-3-*O*-glucoside	8.28	477.1106	477.10385	$C_{22}H_{22}O_{12}$	-0.5	-ve	151, 243, 271, 271, 285, 285, 289, 299, 300, 313, 314, 315, 477	Probable	MSBNK-RIKEN-PR040095 [2]
**2.2.12**	(+)-3 3' 4' 5 7-Pentahydroxyflavan; (+)-catechin	8.81	289.1143	289.07175	$C_{15}H_{14}O_6$	1	-ve	289	Probable	MSBNK-RIKEN-PR100687 [2]
**2.2.13**	Kaempferol-7-neohesperidoside	8.88	593.1588	593.15118	$C_{27}H_{30}O_{15}$	0.6	-ve	151, 193, 227, 289, 593	Probable	[25] MSBNK-Fiocruz-FIO00175 [2]
**2.2.14**	Acacetin	9.52	283.0645	283.06119	$C_{16}H_{12}O_5$	-0.1	-ve	117, 133, 157, 195, 211, 239, 283	Probable	MSBNK-BS-BS003497 [2]
**2.2.15**	Peonidin; 3,4',5,7-tetrahydroxy-3'-methoxyflavylium	12.41	301.0701	301.0707	$C_{16}H_{13}O6^{+}$	-2.0	+ve	255, 257, 258, 268, 269, 273, 283, 285, 286, 300, 301	Probable	MSBNK-RIKEN-PR100907 [2]
**2.2.16**	3 5 7-trihydroxy-4'-methoxyflavone; Diosmetin	12.41	299.0592	299.05612	$C_{16}H_{12}O_6$	0.5	-ve	123, 133, 135, 188, 200, 211, 227, 228, 239, 240, 255., 256, 283, 284, 285, 299, 300	Probable	MSBNK-BS-BS003184 [2]
**2.3. Coumarins, stilbenes, phenolic acids:**										

**2.3.1**	*Trans-ortho*-Coumaric Acid	1.31	163.061	163.04007	$C_9H_8O_3$	2.1	-ve	117, 119, 162	Probable	MSBNK-Keio_Univ-KO000443 [2]
**2.3.2**	Vanillic acid glucoside	5.01	329.0911	329.0878	$C_{14}H_{18}O_9$	10.03	-ve	329	Tentative	[19]
**2.3.3**	Salicylic acid	5.89	137.0252	137.02441	$C_7H_6O_3$	-0.7	-ve	65, 93, 137	Probable	MSBNK-Eawag-EQ369656 [2]
**2.3.4**	5-Methoxysalicylic acid	6.43	167.0358	167.03499	$C_8H_8O_4$	0.2	-ve	108, 152, 167	Probable	MSBNK-RIKEN_ReSpect-PS104208 [2]
**2.3.5**	Scopoletin	7.26	193.0497	193.04953	$C_{10}H_8O_4$	1.0	+ve	149, 150., 178, 193	Probable	MSBNK-IPB_Halle-PB002203 [2]
**2.3.6**	Salicylic acid	8.10	137.0253	137.02441	$C_7H_6O_3$	-0.7	-ve	65, 93, 137	Probable	MSBNK-Eawag-EQ369656 [2]
**2.3.7**	*E*-3,4,5'-Trihydroxy-3'-glucopyranosylstilbene	9.37	405.1601	405.11911	$C_{20}H_{22}O_9$	-0.3	-ve	159, 200, 405	Probable	MSBNK-RIKEN-PR100927 [2]
**2.3.8**	Sinapyl aldehyde	11.22	207.0674	207.06628	$C_{11}H_{12}O_4$	1.6	-ve	77, 148, 149, 177, 207	Probable	MSBNK-RIKEN_ReSpect-PS079103 [2]
**2.4. Triterpenoid derivatives:**										
**2.4.1**	2α,3β,23,24-Tetrahydroxyurs-12-en-28-oic acid	12.60	503.3431	503.3378	$C_{30}H_{48}O_6$	10.53	-ve	503	Tentative	[19]
**2.4.2**	Salsolin A; 3β,11β,24,30-Tetrahydroxyolean-12-en-28-oic acid	12.60	503.3431	503.3378	$C_{30}H_{48}O_6$	-10.5	-ve	329, 401, 427, 457, 459, 485, 501, 503	Tentative	[21,26]
**2.4.3**	Salsoloside C	13.62	925.4938	925.4802	$C_{47}H_{74}O_{18}$	14.70	-ve	925	Tentative	[19]
**2.4.4**	Salsolin B; 2α,3β,23,24-Tetrahydroxyurs-12-en-28-oic acid	15.20	503.3441	503.3378	$C_{30}H_{48}O_6$	-11.9	-ve	73, 385, 395, 407, 411, 413, 423, 429, 443, 452, 459, 467, 485, 502	Tentative	[21,26]
**2.4.5**	Salsolic acid; 3β,6α,24-Trihydroxyolean-12-en-28-oic acid	17.512	487.3487	487.3429	$C_{30}H_{48}O_5$	-12.5	-ve	159, 277, 385, 385, 395, 411,	Tentative	[21,27]

								425, 427, 429, 441, 443, 469, 485, 487		
**2.5. Amino acid derivatives:**										
**2.5.1**	Histidine	1.19	156.0416	156.07675	$C_6H_9N_3O_2$	0.7	+ve	112, 156	Probable	MSBNK-Washington_State_Univ-BML01070 [2]
**2.5.2**	L-Glutamic acid	1.20	146.0469	146.04588	$C_5H_9NO_4$	-0.4	-ve	102, 128, 146	Probable	MSBNK-EPA-ENTACT_AGILENT001254[2]
**2.5.3**	*N*-Acetyl-beta-alanine	1.44	130.088	130.05096	$C_5H_9NO_3$	-1	-ve	58, 88 130	Probable	MSBNK-Keio_Univ-KO000218 [2]
**2.6. Miscellaneous carboxylic acids:**										
**2.6.1**	Methylsuccinic acid	1.06	130.9842	131.03499	$C_5H_8O_4$	0.1	-ve	86, 130, 131	Probable	MSBNK-RIKEN-PR100834 [2]
**2.6.2**	2-Mercaptoethanesulfonic acid	1.26	141.0676	140.96857	$C_2H_6O_3S_2$	0.6	-ve	141	Probable	MSBNK-RIKEN-PR100753 [2]
**2.6.3**	Maleic acid	1.31	115.0042	115.00368	$C_4H_4O_4$	-0.4	-ve	71	Probable	MSBNK-Keio_Univ-KO001363 [2]
**2.6.4**	Isocitrate	1.35	191.0209	191.01973	$C_6H_8O_7$	1	-ve	57, 59, 67., 85, 87, 191	Probable	MSBNK-Keio_Univ-KO001164[2]
**2.6.5**	2-Isopropylmalic acid	5.37	175.0632	175.0612	$C_7H_{12}O_5$	0.5	-ve	59, 85, 86, 87, 113, 115, 116, 129, 131, 175	Probable	MSBNK-Keio_Univ-KO001236[2]
**2.6.6**	D-3-Phenyllactic acid	8.07	165.0572	165.05573	$C_9H_{10}O_3$	-1.6	-ve	74, 103, 117, 119, 147, 165	Probable	MSBNK-Keio_Univ-KO001712 [2]
**2.6.7**	Shikimic acid	9.07	173.1199	173.08194	$C_8H_{14}O_4$	0.2	-ve	55, 99, 109, 127, 129, 137, 173	Probable	MSBNK-Keio_Univ-KO001791[2]

**2.6.8**	(+-)-Jasmonic acid	11.46	209.1201	209.11832	$C_{12}H_{18}O_3$	-0.1	-ve	109, 165, 209	Probable	MSBNK-Washington_State_Univ-BML00677[2]
**2.7. Nucleobases, Nucleosides, and Nucleotides:**										
**2.7.1**	Adenine	1.50	136.0611	136.06177	$C_5H_5N_5$	1.2	+ve	119, 136	Probable	MSBNK-EPA-ENTACT_AGILENT001281[2]
**2.7.2**	Uridine 5'-monophosphate	5.49	323.121	323.02859	$C_9H_{13}N_2O_9P$	-0.1	-ve	96.96, 323.1244	Probable	MSBNK-RIKEN_ReSpect-PS037208 [2]
**2.7.3**	Dihydroorotate	5.99	157.0517	157.02548	$C_5H_6N_2O_4$	-0.5	-ve	113, 157	Probable	MSBNK-RIKEN_ReSpect-PS063007[2]
**2.7.4**	Thymidine-5'-monophosphate	6.89	321.157	321.04932	$C_{10}H_{15}N_2O_8P$	0.8	-ve	96, 321, 322	Probable	MSBNK-RIKEN_ReSpect-PS071009 [2]
**2.7.5**	2'-Deoxyuridine	7.41	227.1313	227.06735	$C_9H_{12}N_2O_5$	-0.3	-ve	226, 227	Probable	MSBNK-RIKEN_ReSpect-PS063307 [2]
**2.7.6**	Thymine	8.81	125.0982	125.03565	$C_5H_6N_2O_2$	-0.9	-ve	125	Probable	MSBNK-Keio_Univ-KO001886[2]
**2.7.7**	Thymidine	16.62	241.1837	241.08299	$C_{10}H_{14}N_2O_5$	-0.5	-ve	59, 181, 197, 225, 241	Probable	MSBNK-Keio_Univ-KO001882[2]
**2.8. Oligosaccharides and sugar derivatives:**										
**2.8.1**	Mucate (*meso*-galactaric acid)	1.29	209.0686	209.03029	$C_6H_{10}O_8$	0.7	-ve	57, 85, 129, 159, 209	Probable	MSBNK-Keio_Univ-KO001427[2]
**2.8.2**	D-(+)-Raffinose	5.67	503.146	503.16177	$C_{18}H_{32}O_{16}$	-0.4	-ve	143, 161, 167, 181, 203, 239, 239, 249, 305, 341, 401, 441, 503	Probable	MSBNK-Metabolon-MT000046 [2]
**2.9. Vitamins:**										

**2.9.1**	(-)-Riboflavin (Vitamin B2)	7.67	377.1212	377.14557	$C_{17}H_{20}N_4O_6$	-0.4	+ve	137, 302, 375, 377	Probable	MSBNK-BGC_Munich-RP013101 [2]
**2.10. Sterols and fatty acid derivatives:**										
**2.10.1**	9,12,13-Trihydroxy octadeca-10(*E*),15(*Z*)-dienoic acid	11.18	327.221	327.2173	$C_{18}H_{32}O_5$	11.31	-ve	327	Tentative	[19]
**2.10.2**	Amasterol	22.46	411.3173	411.3269	$C_{28}H_{44}O_2$	-23.34	-ve	411	Tentative	[19]

[1] Confidence levels were assigned according to the identification scale proposed by Schymanski et al. [18]. No **Standard** (Level 1: Confirmed structure) identifications were achieved, as authentic reference standards were not analyzed. **Probable** (Level 2) identifications were assigned based on unambiguous library spectral matches (Level 2a) or structural rationales supported by diagnostic MS2 fragmentation patterns (Level 2b). **Tentative/ putative** (Level 3) identifications denote candidates where evidence exists for a structural class or specific compound, but the assignment remains speculative due to high mass errors (> ±10 ppm) or unresolved structural features.

Table S3. Phytochemical compounds identified in *S. parviflora* AME by LC-MS/MS in positive and negative ionization modes.

No.	Identified compounds	R_t (min)	Precursor (*m/z*)	Reference (*m/z*)	Formula	Error (ppm)	Mode	MS² spectrum	Confidence level ¹	References
**3.1. Flavonoids and flavonoid glycosides**										
**3.1.1**	Okanin-4'-*O*-glucoside	5.56	449.1173	449.10895	$C_{21}H_{22}O_{11}$	-2.5	-ve	125, 165, 229, 241, 243, 272, 272.06, 273, 285, 287, 313 389, 449	Probable	MSBNK-RIKEN-PR306103[2]
**3.1.2**	Luteolin-3', 7-di-*O*-glucoside	5.94	609.1555	609.14612	$C_{27}H_{30}O_{16}$	0.8	-ve	255, 283.01, 283.03 284, 285, 609, 610, 611	Probable	MSBNK-BS-BS003236 MSBNK-BS-BS003234[2]
**3.1.3**	Quercetin-3,4'-*O*-di-beta-glucopyranoside	6.65	625.1533	625.14105	$C_{27}H_{30}O_{17}$	-2.4	-ve	178.9, 255, 271 299, 300, 301, 463,625.	Probable	MSBNK-RIKEN-PR100914 MSBNK-RIKEN-PR100915 [2] [28-31]
**3.1.4**	Luteolin-6-*C*-glucoside	6.78	449.1068	449.10785	$C_{21}H_{20}O_{11}$	-0.9	+ve	109, 137, 163, 165, 177, 203, 215, 245, 255, 283, 286, 287 295, 297, 299, 300, 301, 311, 313, 321, 323, 324, 325, 327.05, 327.08, 329, 337, 339, 349, 353, 355, 365, 367 369, 377, 383, 395, 413, 431, 449	Probable	MSBNK-RIKEN-PR301968 [2]
**3.1.5**	Luteolin-8-*C*-glucoside; Orientin	7.49	447.1005	447.09329	$C_{21}H_{20}O_{11}$	-0.8	-ve	327, 447, 448	Probable	MSBNK-BS-BS003370 [2]
**3.1.6**	Luteolin-4'-*O*-glucoside	7.64	447.0987	447.09329	$C_{21}H_{20}O_{11}$	1.8	-ve	285, 286	Probable	MSBNK-BS-BS003262 [2]
**3.1.7**	Luteolin-7-*O*-glucoside; Cynaroside	8.16	447.1011	447.09329	$C_{21}H_{20}O_{11}$	-0.8	-ve	285	Probable	MSBNK-IPB_Halle-PN000059 [2], [28,31]
**3.1.8**	Cyanidin-3-glucoside	8.16	449.1065	449.1073	$C_{21}H_{21}O_{11}$	-0.3	+ ve	213, 287, 449.1, 449.2	Probable	MSBNK-RIKEN-PR040254[2], [31,32]
**3.1.9**	Isorhamnetin-3-*O*-glucoside	8.27	477.1133	477.10385	$C_{22}H_{22}O_{12}$	-1.9	-ve	151, 242, 242, 243, 257, 270, 271, 285, 286, 299, 300, 314, 315	Probable	MSBNK-RIKEN-PR100657[2], [28]

**3.1.10**	Luteolin	10.12	285.0439	285.04047	$C_{15}H_{10}O_6$	0.8	-ve	107,133,149,151, 173, 175, 183, 198, 201, 217, 223, 229, 241, 243, 257, 267, 285, 286	Probable	MSBNK-BS-BS003099 [2], [28-31]
**3.1.11**	3 5 7-Trihydroxy-4'-methoxyflavone; Kaempferide	11.49	299.061	299.05612	$C_{16}H_{12}O_6$	-1	-ve	150, 151,285, 285, 300, 300.08122:14	Probable	MSBNK-RIKEN-PR020013 [2]
**3.1.12**	3'-Methoxy-4',5,7-trihydroxyflavonol; Isorhamnetin	15.92	315.0556	315.05103	$C_{16}H_{12}O_7$	-0.4	-ve	227, 243, 255, 255.05, 271, 300, 301, 315, 316	Probable	MSBNK-RIKEN-PR040022 [2]
**3.2. Phenolic acids**										
**3.2.1**	Salicylic acid	6.02	137.0243	137.02441	$C_7H_6O_3$	1.7	-ve	65,93, 137	Probable	MSBNK-Athens_Univ-AU238262 [2] [30,31,33]
**3.2.2**	*p*-Hydroxybenzoic acid	8.28	137.0252	137.02441	$C_7H_6O_3$	-0.8	-ve	65, 93	Probable	MSBNK-BAFG-CSL2311091424 [2], [30,31,33]
**3.3. Amino acids**										
**3.3.1**	L-Glutamine	1.20	147.0757	147.07642	$C_5H_{10}N_2O_3$	1	+ve	83, 84, 85, 101, 102, 130, 147	Probable	MSBNK-BGC_Munich-RP001301 [2] [32,34]
**3.3.2**	N-Methylalanine	1.23	104.0708	104.0706	$C_4H_9NO_2$	-4.2	+ve	86,87,104	Probable	MSBNK-Keio_Univ-KO003357 [2]
**3.3.3**	DL-alpha,epsilon-Diaminopimelic acid	1.23	191.1024	191.10263	$C_7H_{14}N_2O_4$	1.7	+ve	128, 191	Probable	MSBNK-RIKEN-PR100129 [2] [32,35]
**3.3.4**	Glycine betaine; trimethylglycine; betaine	1.24	118.086	118.08626	$C_5H_{11}NO_2$	0.8	+ve	57, 58, 74, 102, 118	Probable	MSBNK-IPB_Halle-PB001709[2], [32,35]
**3.3.5**	L-Proline	1.25	116.0704	116.0706	$C_5H_9NO_2$	-0.7	+ve	53, 68, 70, 116	Probable	MSBNK-Antwerp_Univ-METOX_P100401_EF88[2] [32,34]
**3.3.6**	Norvaline	1.30	116.0729	116.0717	$C_5H_{11}NO_2$	-1.1	-ve	116	Probable	MSBNK-Keio_Univ-KO001508- [2]
**3.3.7**	Pipecolate	1.32	130.0872	130.08626	$C_6H_{11}NO_2$	-5.8	+ve	69, 70, 77, 80, 82 112	Probable	MSBNK-Keio_Univ-KO003743 MSBNK-Keio_Univ-KO003746[2]

**3.3.8**	L-Valine	1.32	118.0862	118.08626	$C_5H_{11}NO_2$	-0.9	+ve	55, 58, 59, 72	Probable	MSBNK-Antwerp_Univ-METOX_P100403_EF88[2] [30,34]
**3.3.9**	Threonine	1.38	118.0514	118.05096	$C_4H_9NO_3$	0	-ve	72.00, 72.01 74, 118.	Probable	MSBNK-MPI_for_Chemical_Ecology-CE000408 MSBNK-MPI_for_Chemical_Ecology-CE000409[2], [32,34]
**3.3.10**	L-5-Oxoproline	1.44	128.0364	128.03532	C_5H7NO_3	-1.2	-ve	128	Probable	MSBNK-RIKEN-PR100586[2] [32,35]
**3.3.11**	L-Norleucine	1.66	132.101	132.1019	$C_6H_{13}NO_2$	2.4	+ve	67,86, 132	Probable	MSBNK-BGC_Munich-RP002202 MSBNK-BGC_Munich-RP002201[2]
**3.3.12**	L-(-)-Phenylalanine	3.09	164.0735	164.0717	$C_9H_{11}NO_2$	-2.3	-ve	72, 91, 103 147, 164	Probable	MSBNK-RIKEN-PR100497[2] [30,32,34]
**3.3.13**	L-Tryptophan	4.86	203.0845	203.0826	$C_{11}H_{12}N_2O_2$	0.9	-ve	72, 74, 116, 117, 159 186, 203	Probable	MSBNK-BGC_Munich-RP000512[2], [30,32,34]
		4.87	205.0967	205.09715	$C_{11}H_{12}N_2O_2$	0.2	+ve	51, 74, 77, 89, 91, 103, 115, 116, 117, 118, 127, 130, 132 142, 143, 144, 146, 149, 159	Probable	MSBNK-Antwerp_Univ-METOX_P100401_F638[2] [30,32,34]
**3.4. Carboxylic ~~acids~~**										
**3.4.1**	Succinic acid	1.86	117.0196	117.01933	$C_4H_6O_4$	0.8	-ve	73, 99,117	Probable	MSBNK-Eawag-EQ01155052[2] [32,35]
**3.4.2**	2-Isopropylmalic acid	5.50	175.0632	175.0612	$C_7H_{12}O_5$	-0.2	-ve	85, 113,115,131, 157, 175	Probable	MSBNK-RIKEN-PR100745[2]

**3.4.3**	1*H*-indole-3-carboxylic acid	8.55	160.0409	160.04041	$C_9H_7NO_2$	1.9	-ve	116,117.051, 117.058,160	Probable	MSBNK-RIKEN-PR100988[2]
**3.5. Fatty acids and their derivatives**										
**3.5.1**	Citramalic acid	1.47	147.0311	147.02989	$C_5H_8O_5$	0.1	-ve	85, 101, 103, 129, 147	Probable	MSBNK-MetaboLights-ML002851[2]
**3.5.2**	13*S*-hydroxy-6*Z*,9*Z*,11*E*-octadecatrienoic acid	7.29	293.2163	293.2122	$C_{18}H_{30}O_3$	+13.9	-ve	293, 294	Tentative	MSBNK-Chubu_Univ-UT000047 [2]
**3.5.3**	13*S*-hydroxy-9*Z*,11*E*,15*Z*-octadecatrienoic acid	18.68	295.227	295.2273	$C_{18}H_{30}O_3$	−1.02	+ve	97.06, 97.10, 109.06, 109.10, 117, 119, 121, 123.08, 123.12, 125, 129, 131, 133, 135.08, 135.12, 137, 143, 147, 149, 151, 153, 161, 163.11, 163.145, 165, 171, 173, 175, 177, 179, 185, 189, 193, 199, 203, 207, 217, 221, 231, 235, 240, 241, 259, 276, 277, 295	Probable	MSBNK-Chubu_Univ-UT000062 MSBNK-Chubu_Univ-UT000048 [2]
**3.5.4**	*gamma*-Linolenic acid	21.38	277.2216	277.21732	$C_{18}H_{30}O_2$	-0.7	-ve	83, 97, 205, 233, 257 259, 277	Probable	MSBNK-Antwerp_Univ-METOX_N104927_9CB7 [2]
**3.5.5**	Monostearin	23.89	359.3149	359.3161	$C_{21}H_{42}O_4$	−3.34	+ve	55, 57.03, 57.07, 67, 69, 70, 71, 75, 81, 83, 85, 95, 97, 99, 103, 109, 111, 113, 117, 123, 125, 127, 137, 141, 151, 153, 165, 167, 179, 249, 267, 284, 285, 341, 359	Probable	MSBNK-Fac_Eng_Univ_Tokyo-JP005843 [2]
**3.6. Nucleosides**										
**3.6.1**	5'-Deoxy-5'-Methylthioadenosine	5.52	298.0975	298.09683	$C_{11}H_{15}N_5O_3S$	-1.8	+ve	55, 57,61, 85, 127, 137	Probable	MSBNK-Keio_Univ-KO002725 [2]
**3.7. Alkyl amides**										
**3.7.1**	Feruloyltyramine	9.71	314.1375	314.1387	$C_{18}H_{19}NO_4$	–3.82	+ve	77, 93, 100, 103,117, 121, 145, 146, 149, 177, 314	Probable	MSBNK-MSSJ-MSJ00002 [2]

										(Figure S21)
**3.7.2**	Erucamide	26.06	338.3408	338.3417	$C_{22}H_{43}NO$	+2.66	+ve	95, 97, 135, 153, 156, 163, 303, 321, 338	Probable	MSBNK-Fiocruz-FIO00883[2] (Figure S20)
**3.8. Glycerophospholipids**										
**3.8.1**	1-(9,12-octadecadienoyl)-sn-glycero-3-phosphocholine; OR 2-Linoleoyl-sn-glycero-3-phosphocholine	16.67	520.3388	520.3403	$C_{26}H_{50}NO_7P$	−2.88	+ve	60, 86, 104, 124, 166, 184, 258, 337, 443, 502, 520	Probable	MSBNK-RIKEN-PR310843[2]
**3.8.2**	1-Hexadecanoyl-sn-glycero-3-phosphocholine; 1-Palmitoyl-sn-glycero-3-phosphocholine	17.35	496.3383	496.3398	$C_{24}H_{50}NO_7P$	−3.02	+ve	56, 60, 71, 81, 86, 98, 104, 124, 184, 258, 313, 478, 496	Probable	MSBNK-BAFG-CSL23111015297[2]

[1] Confidence levels were assigned according to the identification scale proposed by Schymanski et al. [18]. No **Standard** (Level 1: Confirmed structure) identifications were achieved, as authentic reference standards were not analyzed. **Probable** (Level 2) identifications were assigned based on unambiguous library spectral matches (Level 2a) or structural rationales supported by diagnostic MS2 fragmentation patterns (Level 2b). **Tentative/ putative** (Level 3) identifications denote candidates where evidence exists for a structural class or specific compound, but the assignment remains speculative due to high mass errors (> ±10 ppm) or unresolved structural features.

- **TOF MS² spectra of selected compounds identified from *C. gileadensis* aerial parts AME in positive and/or negative ionization modes:**

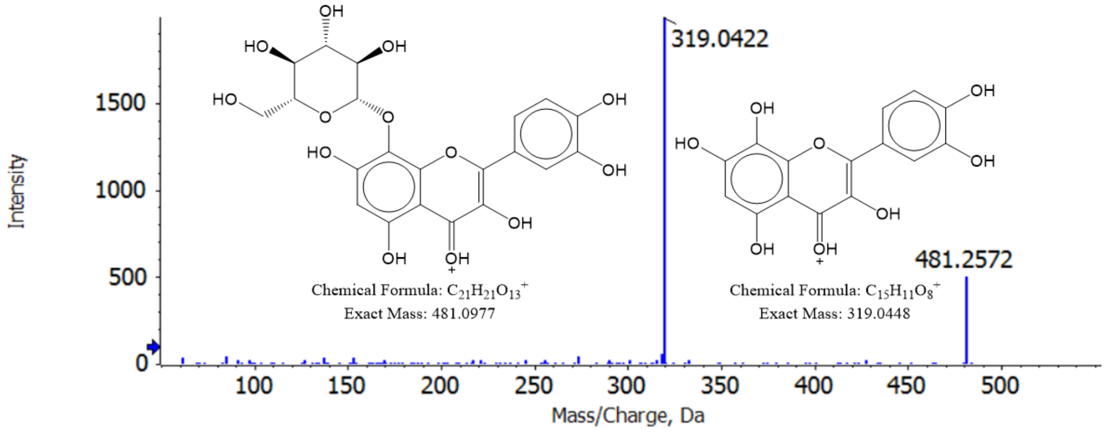

Figure S1. Spectrum of compound **1.1.3** (TOF MS²) for the precursor ion (R_t 6.918 min) at *m/z* 481.2572; gossypin.

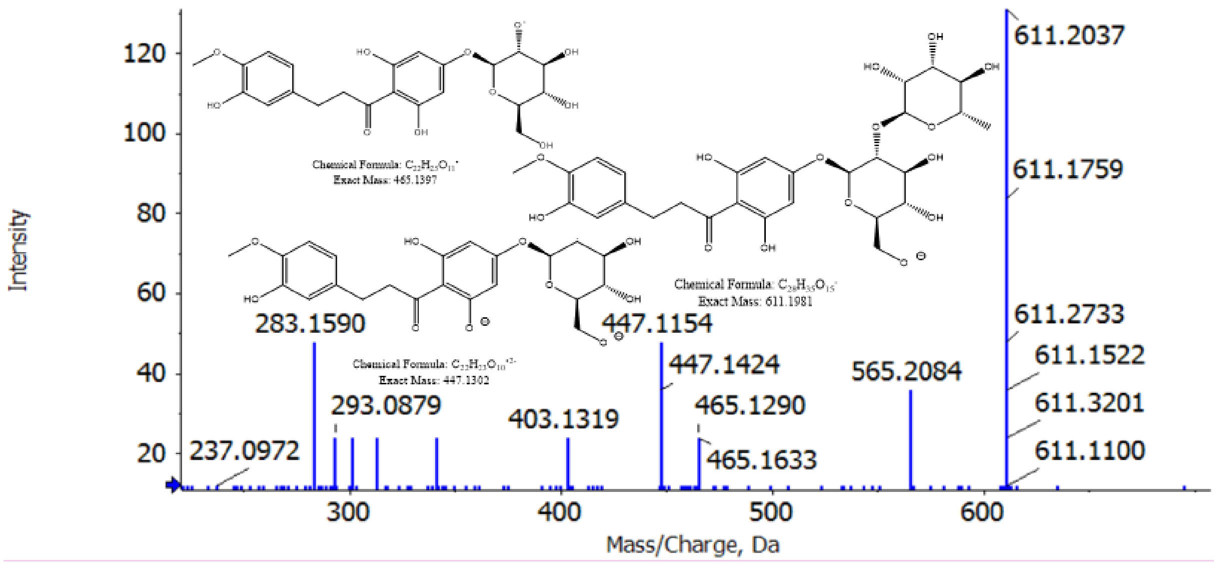

Figure S2. Spectrum of compound **1.1.13** (TOF MS²) for the precursor ion (R_t 8.879 min) at *m/z* 611.2037; neohesperidin dihydrochalcone.

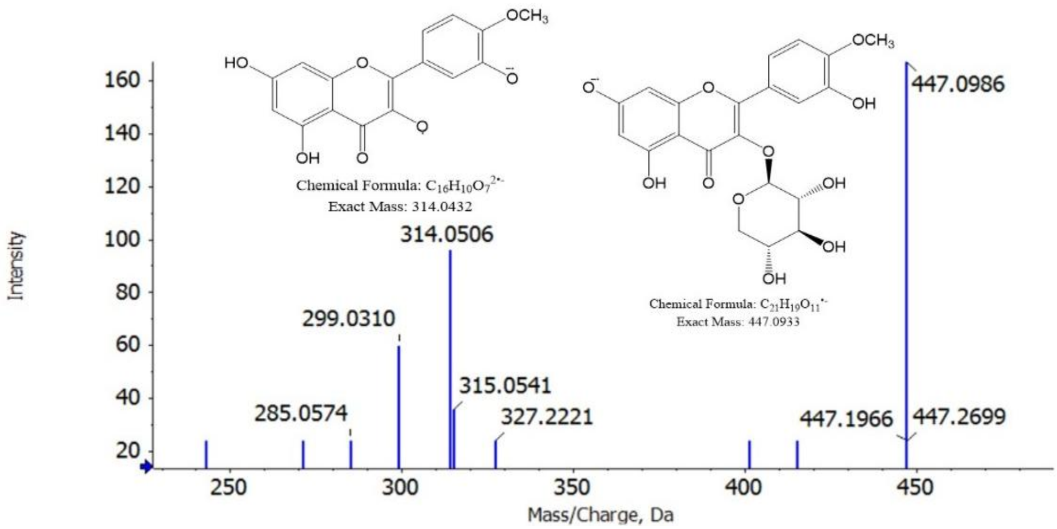

Figure S3. Spectrum of compound **1.1.21** (TOF MS²) for the precursor ion (R_t 10.26 min) at *m/z* 447.0986; isorhamnetin pentoside.

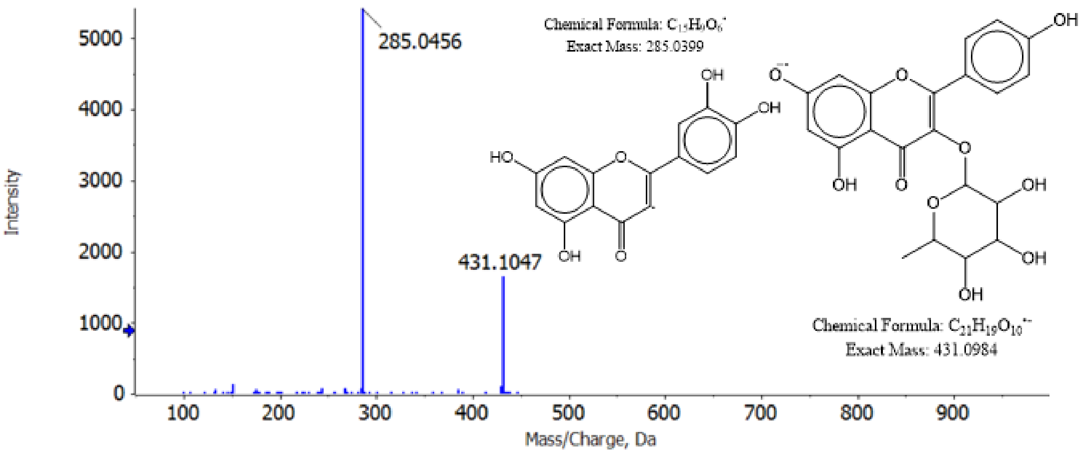

Figure S4. Spectrum of compound **1.1.17** (TOF MS2) for the precursor ion (R_t 9.388 min) at *m*/*z* 431.1047; kaempferol-3-*O*-alpha-L-rhamnoside.

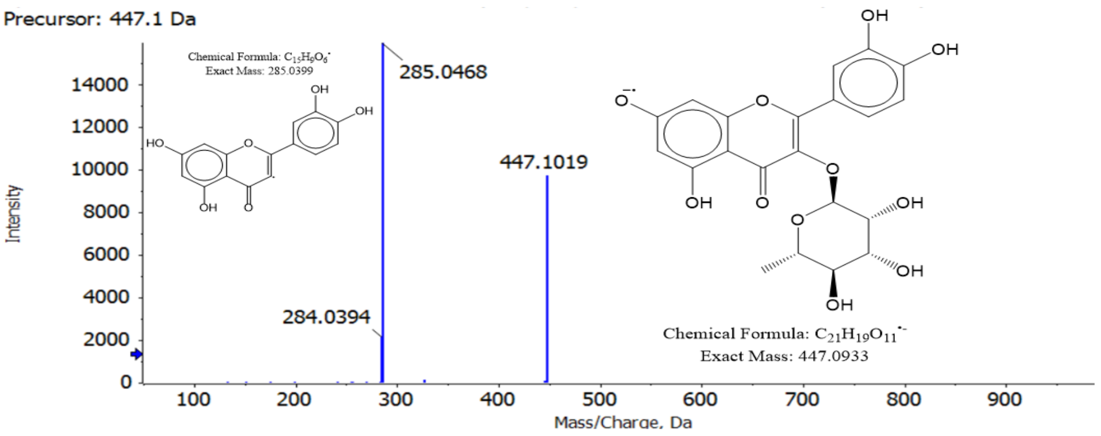

Figure S5. Spectrum of compound **1.1.10** (TOF MS2) for the precursor ion (R_t 7.674 min) at *m*/*z* 447.1019; quercitrin.

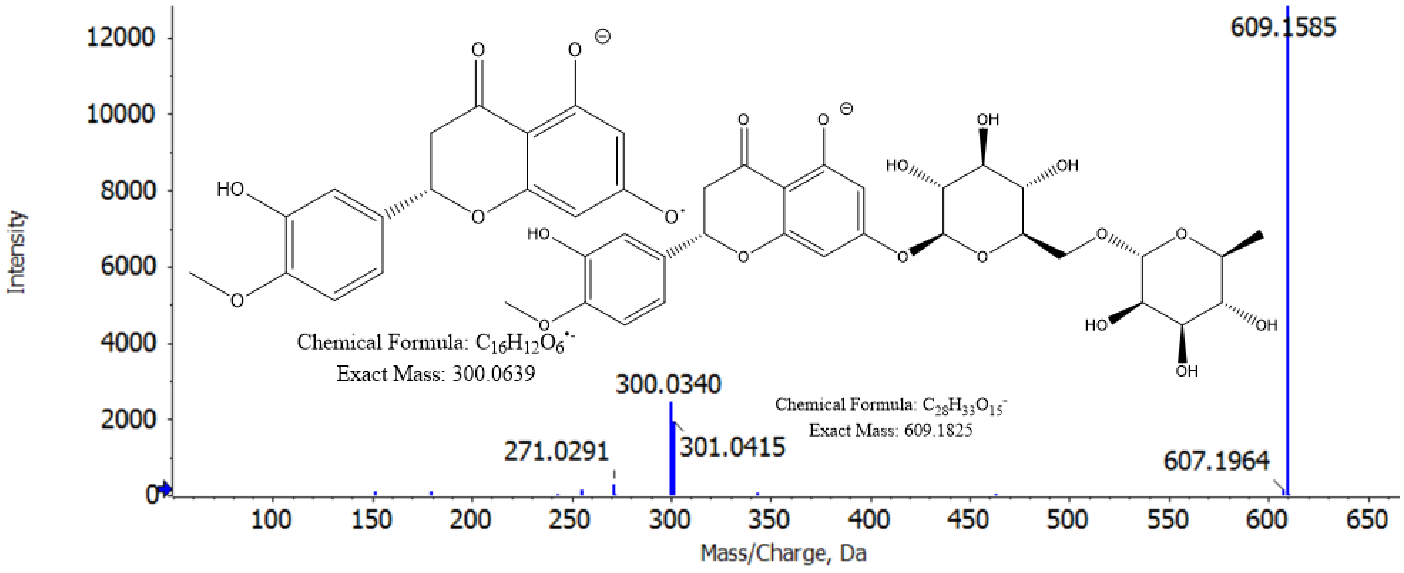

Figure S6. Spectrum of compound **1.1.4** (TOF MS2) for the precursor ion (R_t 7.311 min) at *m*/*z* 609.1585; hesperidin/ neohesperidin.

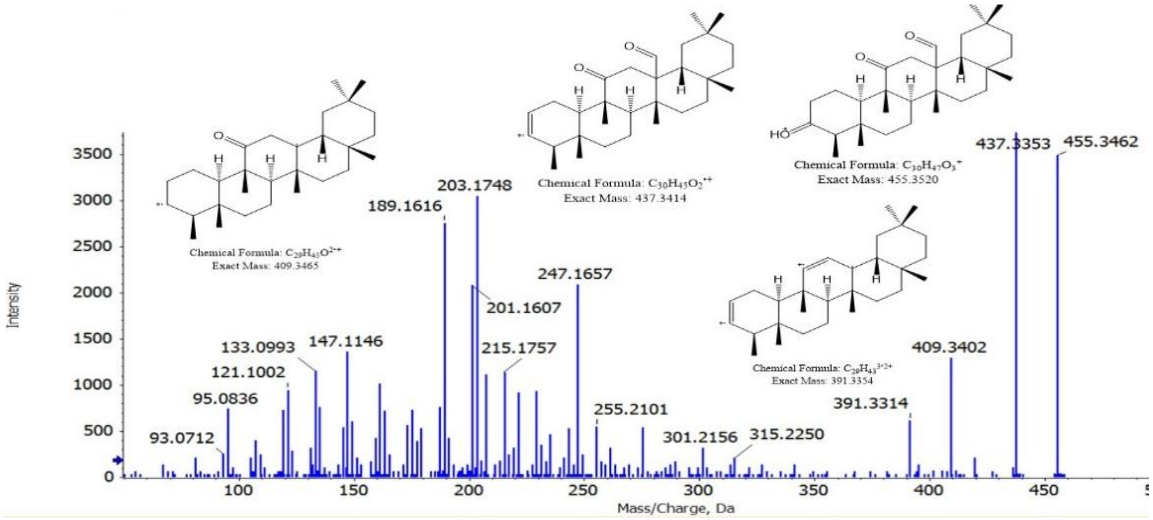

Figure S7. Proposed MS² fragmentation of commigileadin A.

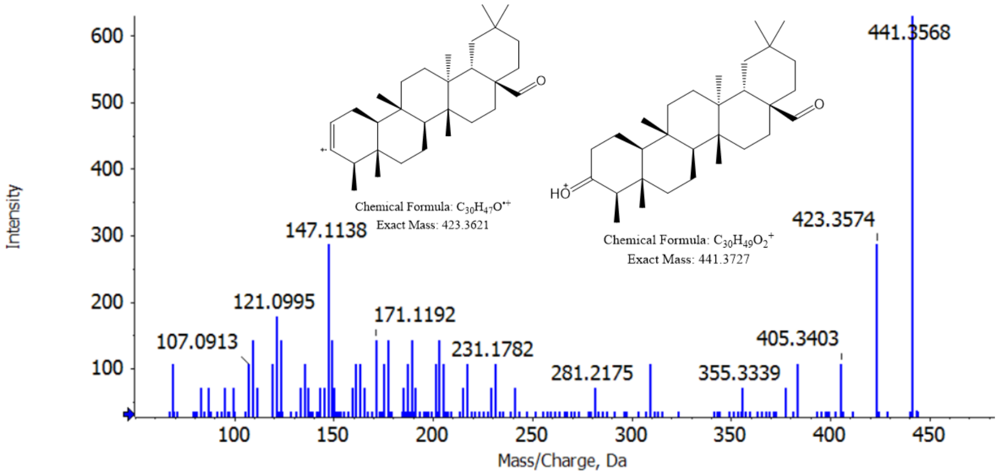

Figure S8. Proposed MS² fragmentation of canophyllal.

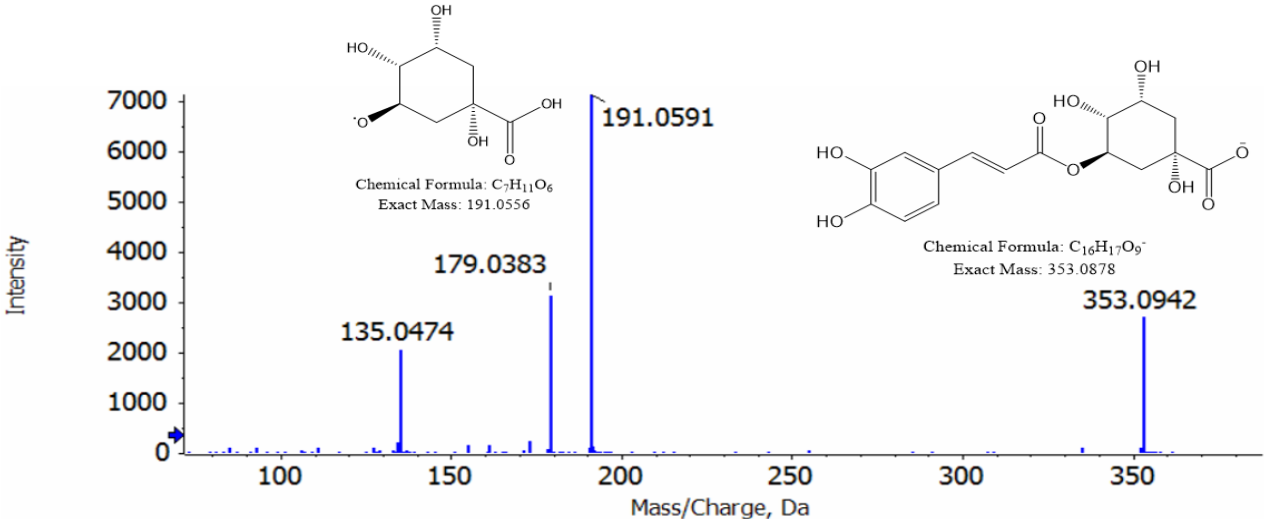

Figure S9. Spectrum of compound **1.2.1** (TOF MS² negative mode) for the precursor ion (R_t 4.73 min) at *m*/*z* 353.0942; chlorogenic acid.

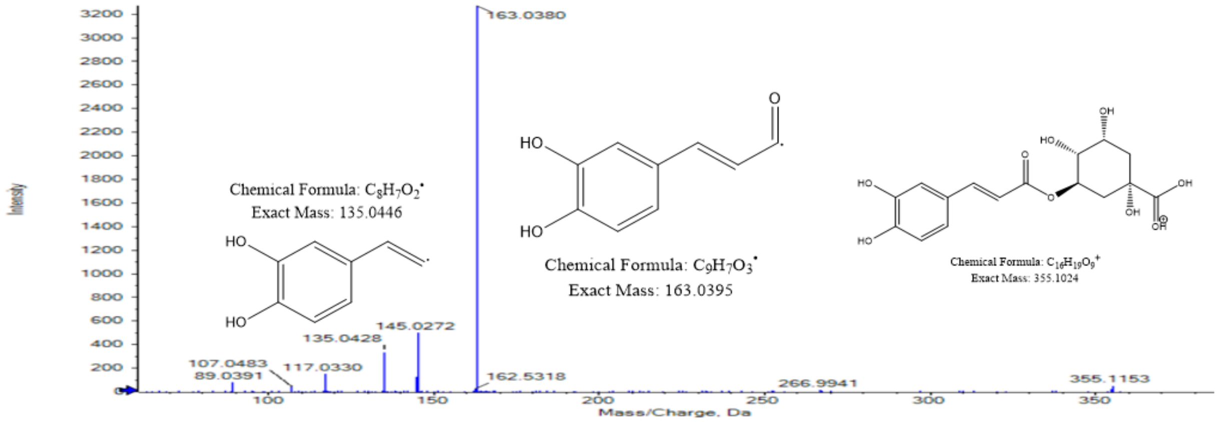

Figure S10. Spectrum of compound **1.2.1** (TOF MS[2] positive mode) for the precursor ion (R_t 4.71 min) at *m/z* 355.1024; Chlorogenic acid.

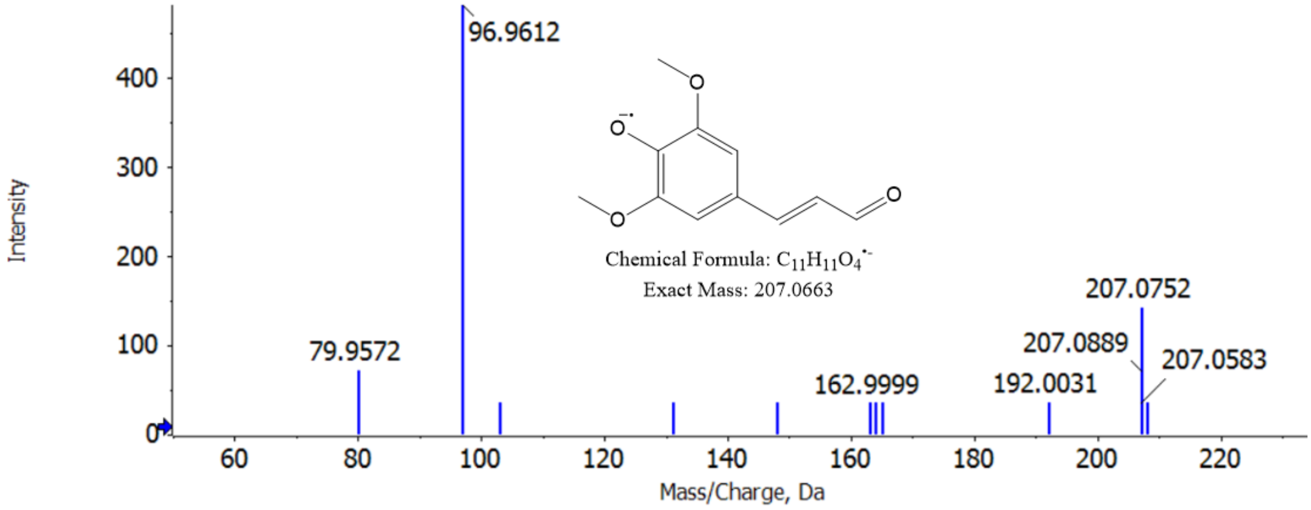

Figure S11. Spectrum of compound **1.2.4** (TOF MS[2]) for the precursor ion (R_t 9.587 min) at *m/z* 207.0752; sinapyl aldehyde.

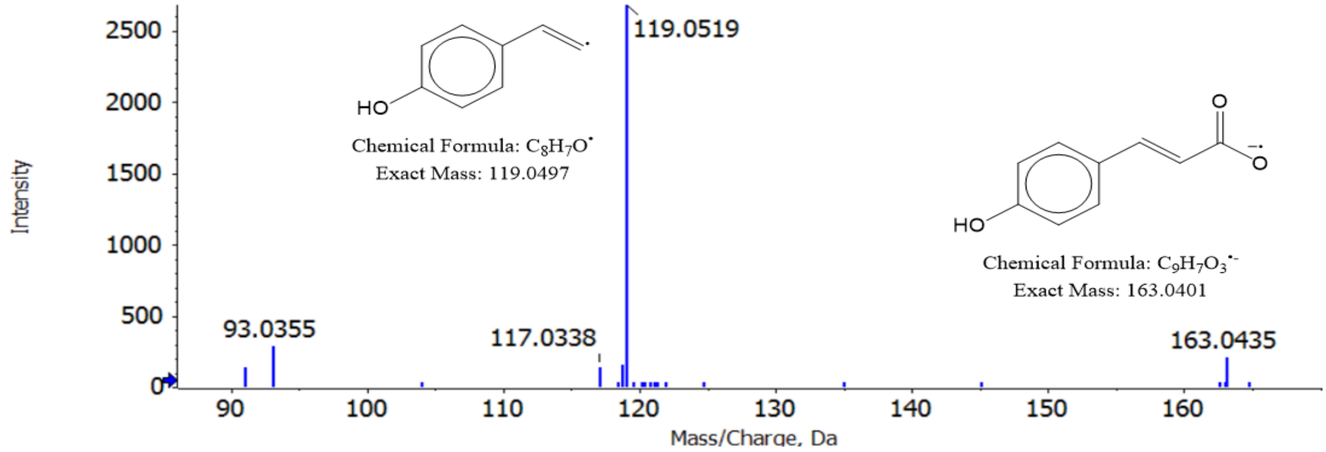

Figure S12. Spectrum of compound **1.2.5** (TOF MS[2]) for the precursor ion (R_t 7.502 min) at *m/z* 163.0435; 3-(4-hydroxyphenyl) prop-2-enoic acid (*p*-coumaric acid).

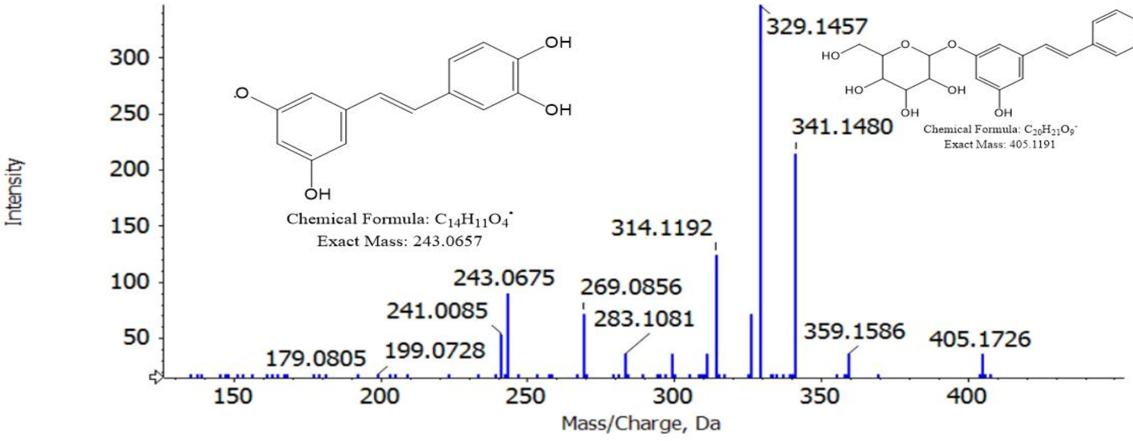

Figure S13. Spectrum of compound **1.4.1** (TOF MS²) for the precursor ion (R_t 9.497 min) at *m*/*z* 405.1726; E-3,4,5'-trihydroxy-3'-glucopyranosylstilbene.

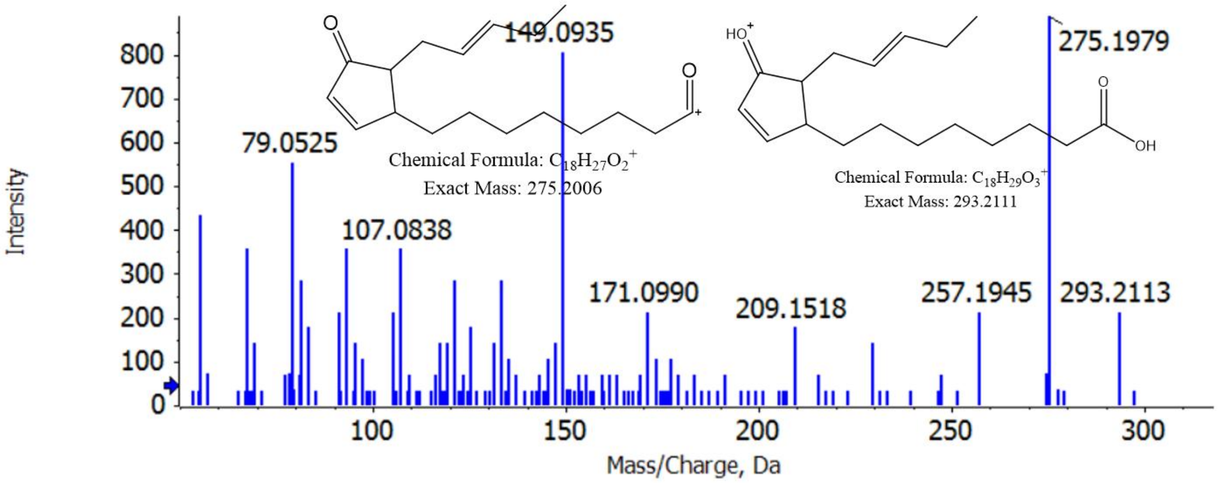

Figure S14. Spectrum of compound **1.6.1.** (TOF MS²) for the precursor ion (R_t 17.471 min) at *m*/*z* 293.2113; 12-Oxo-10,15(*Z*)-phytodienoic acid.

TOF MS2 spectra of selected compounds identified from *S. incanescens* aerial parts AME in positive or negative ionization modes.

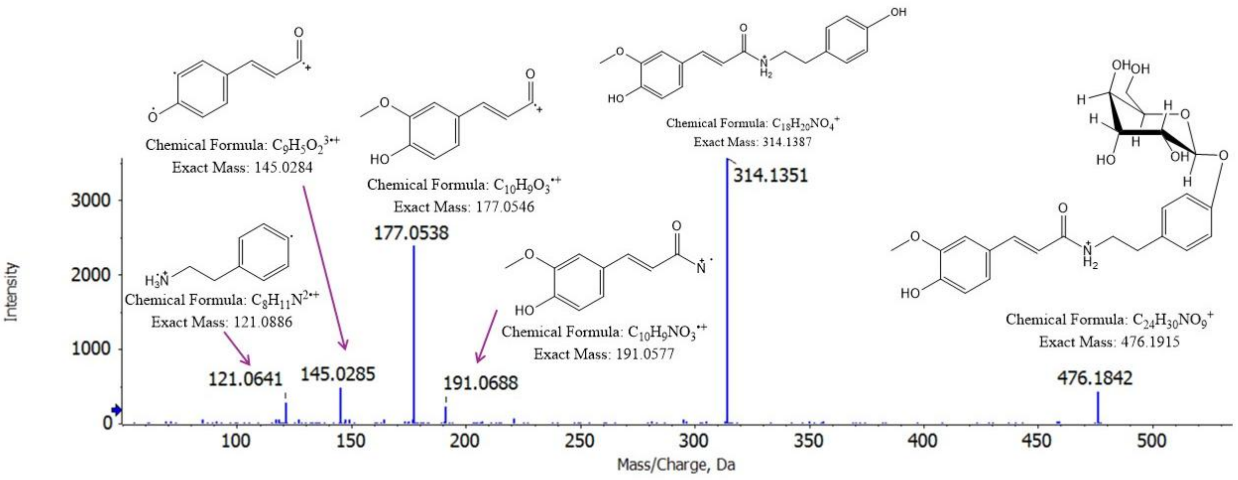

Figure S15. Spectrum of compound **2.1.7** (TOF MS²) for the precursor ion (R_t 7.7287 min) at *m*/*z* 476.1903; *trans*-*N*-feruloyl tyramine-4```-*O*-*β*-D-glucopyranoside.

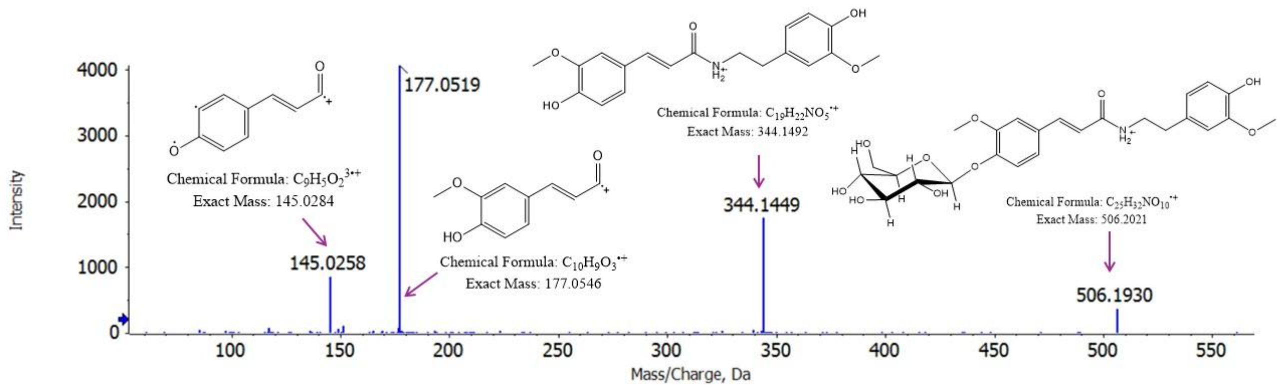

Figure S16. Spectrum of compound **2.1.8** (TOF MS2) for the precursor ion (R_t 7.8222 min) at *m /z* 506.2007; cimicifugamide; *N*-(*trans*-4-*O*-*β*-D-glucopyranoside feruloyl)-3`-*O*-methyldopamine.

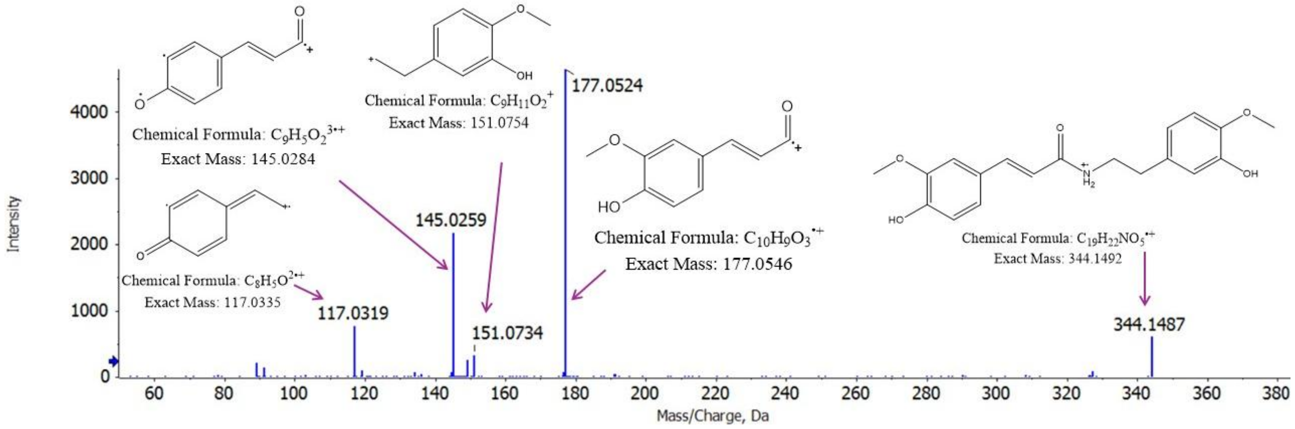

Figure S17. Spectrum of compound **2.1.13** (TOF MS2) for the precursor ion (R_t 9.586783 min) at *m/z* 344.1492; *N*-t*rans*-feruloyl-3-*O*-methyldopamine; *N*-*trans*-feruloyl-4`-*O*-methyldopamine.

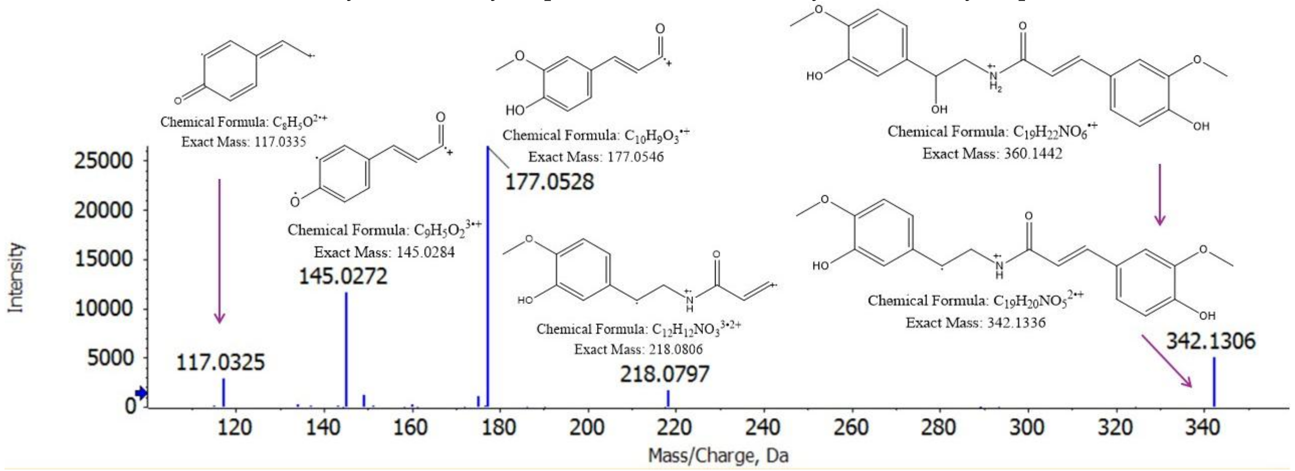

Figure S18. Spectrum of compound **2.1.9** (TOF MS2) for the precursor ion (R_t 8.24435 min) at *m/z* 360.1436; 2`-hydroxy- 3``-methyoxymoupinamide; (*E*)-*N*-(2-hydroxy-2-(3-hydroxy-4-methoxyphenyl)ethyl)-3-(4-hydroxy-3-methoxyphenyl)acrylamide.

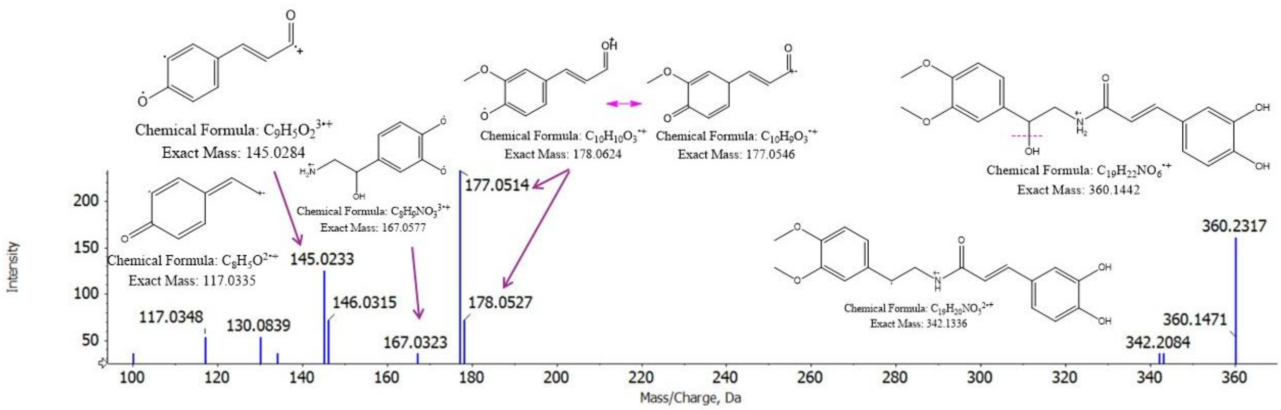

Figure S19. Spectrum of compound **2.1.4** (TOF MS²) for the precursor ion (R_t 6.674 min) at *m/z* 360.1432; *N*-(3`,4`-dimethoxy-cinnamoyl)-norepinephrine.

TOF MS2 spectra of selected compounds identified from *S. parviflora* aerial parts AME in positive or negative ionization modes.

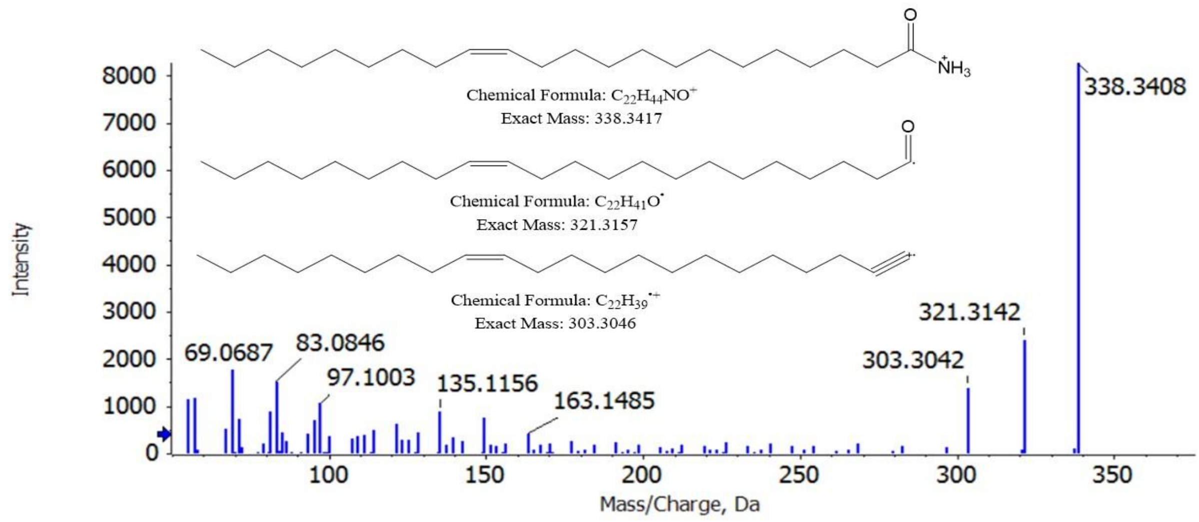

Figure S20. Spectrum of compound **3.7.2** (TOF MS²) for the precursor ion (R_t 26.06 min) at *m/z* 338.3408; erucamide

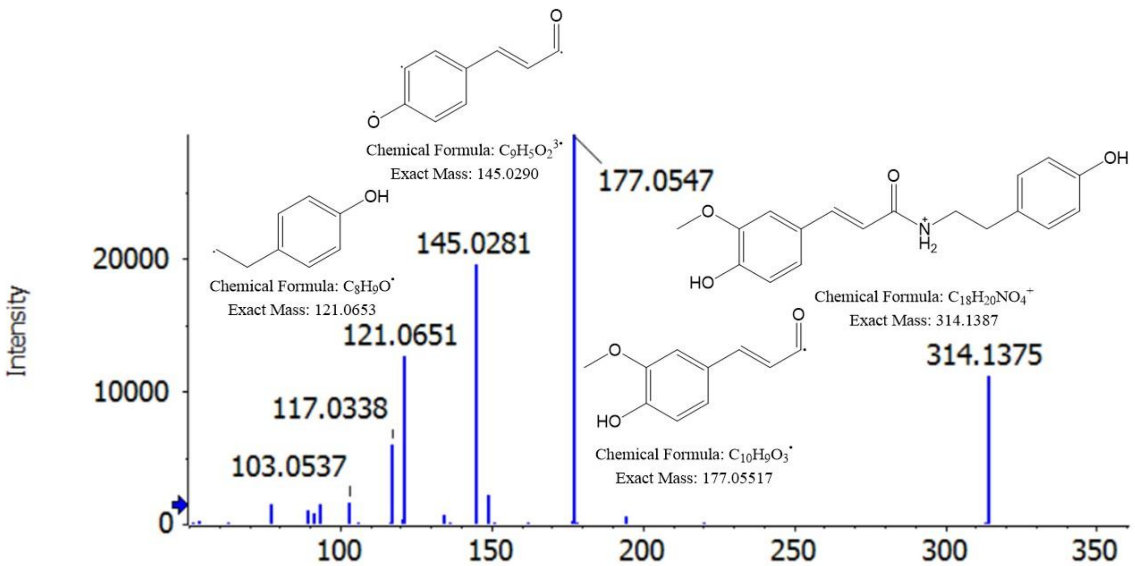

Figure S21. Spectrum of compound **3.7.1** (TOF MS²) for the precursor ion (R_t 9.71 min) at *m/z* 314.1375; feruloyltyramine.

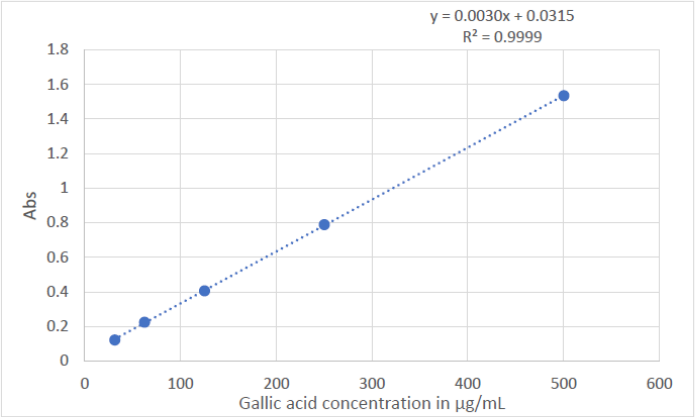

Figure S22. Calibration curve of gallic acid for determination of total phenolic content in the extracts. Y-axis represents the absorbance and X-axis represents gallic acid concentration in µg/mL.

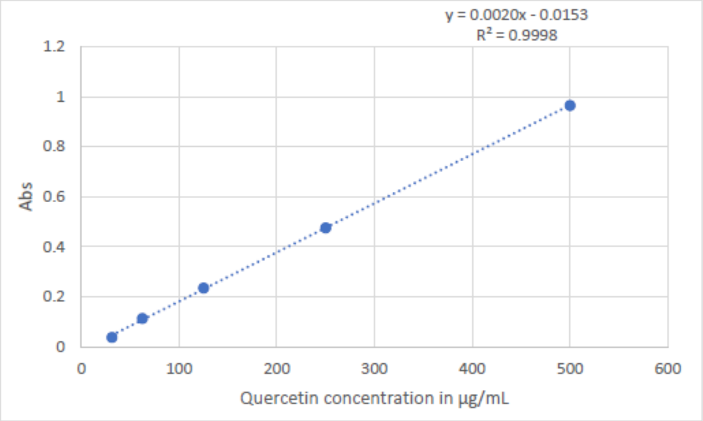

Figure S23. Calibration curve of quercetin for determination of total flavonoid content in the extracts. Y-axis represents the absorbance and X-axis represents quercetin concentration in µg/mL.

Table S4. TPC of the methanol extracts of the aerial parts of *C. gileadensis*, *S. incanescens*, and *S. parviflora.*

Extract	Average absorbance	Substitution in cal. curve equation (µg GA/mg extract)	Total phenolics content (µg gallic acid per mg extract)	Standard deviation
**C. gileadensis**	0.35	106.5	10.65	0.33
S. incanescens	0.11	24.83	2.48	0.20
S. parviflora	0.82	262.94	26.29	2.50

Table S5. TFC of the methanol extracts of the aerial parts of *C. gileadensis*, *S. incanescens*, and *S. parviflora.*

Extract	Average absorbance	Substitution in cal. curve equation (µg Q/mg extract)	Total flavonoid content (µg Q/ mg extract)	Standard deviation
C. gileadensis	0.26	136.65	13.67	1
S. incanescens	0.12	66.32	6.63	0.08
S. parviflora	0.21	113.48	11.35	0.71

<table>
<tr><th>Compound / Class</th><th>Structure</th></tr>
<tr><td>Eritoran
(crystallized molecule)
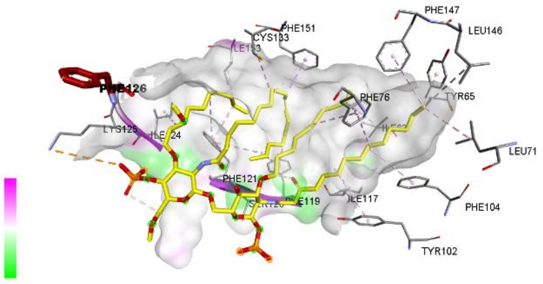
</td><td>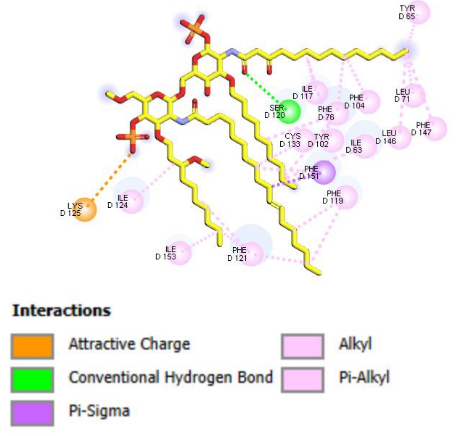
</td></tr>
<tr><td>Isorhamnetin
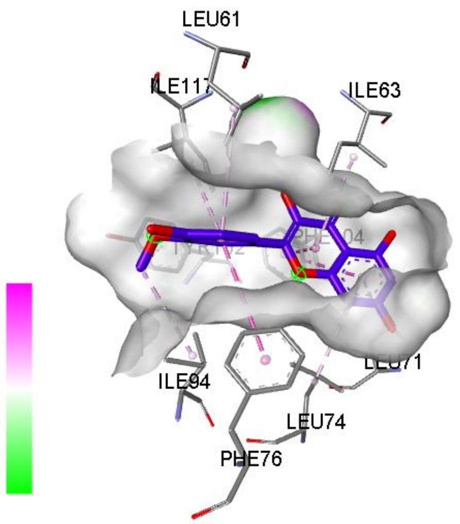
</td><td>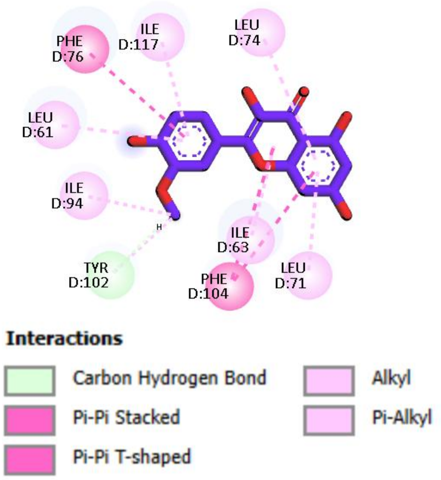
</td></tr>
<tr><td>Syringetin
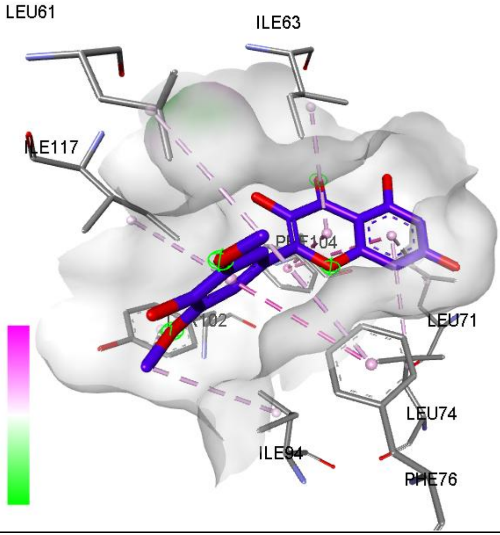
</td><td>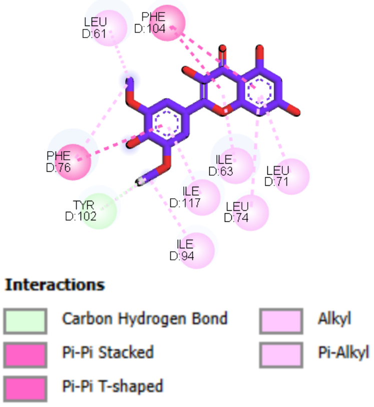
</td></tr>
<tr><td>Daidzein-8-C-glucoside</td><td></td></tr>
</table>

<table>
<tr>
<td>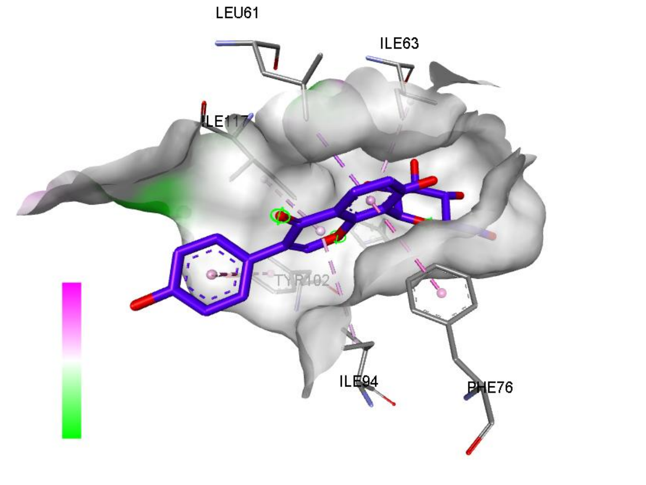
</td>
<td>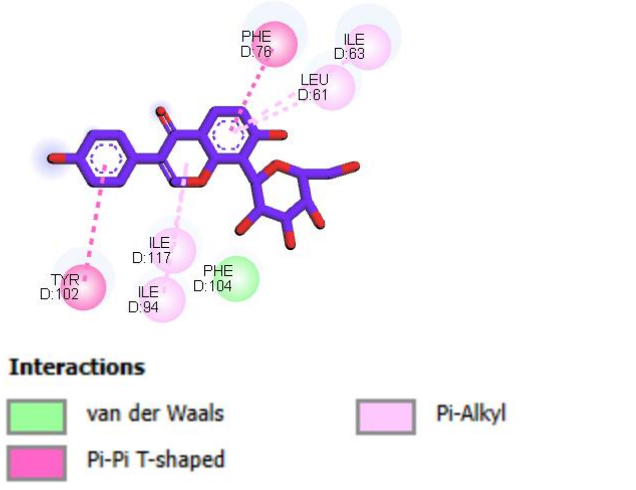
</td>
</tr>
<tr>
<td>N-Feruloyltyramine (Moupinamide)
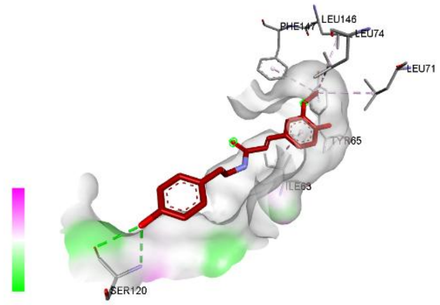</td>
<td>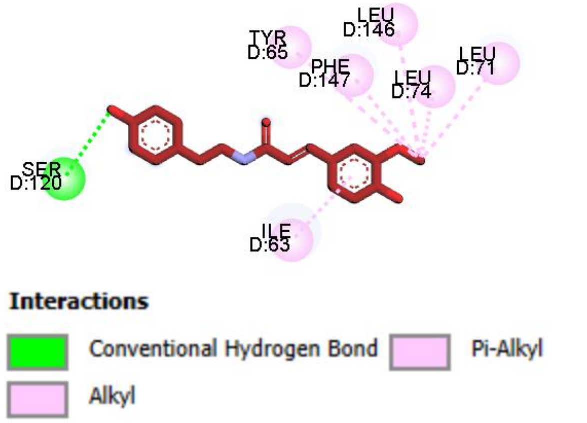
</td>
</tr>
<tr>
<td>N-Caffeoyltyramine
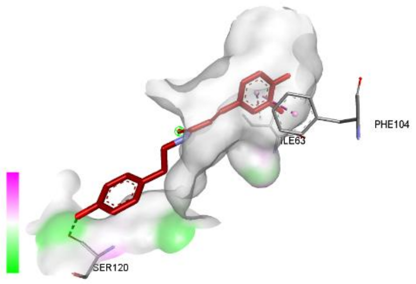
</td>
<td>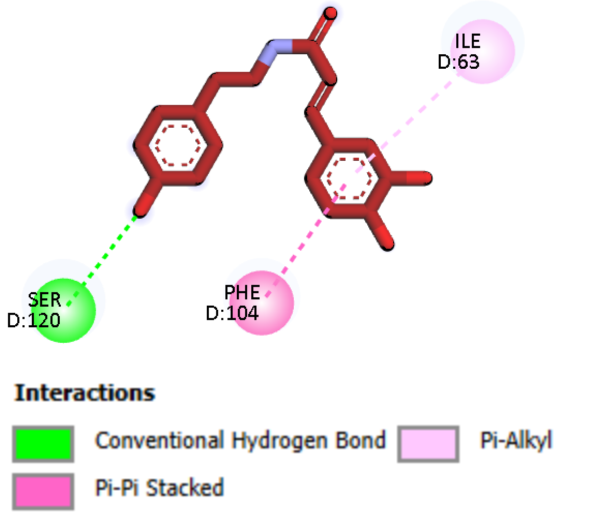
</td>
</tr>
<tr>
<td>(E)-3-(4-Hydroxy-3-methoxyphenyl)-N-(4-methoxyphenethyl)acrylamide</td>
<td></td>
</tr>
</table>

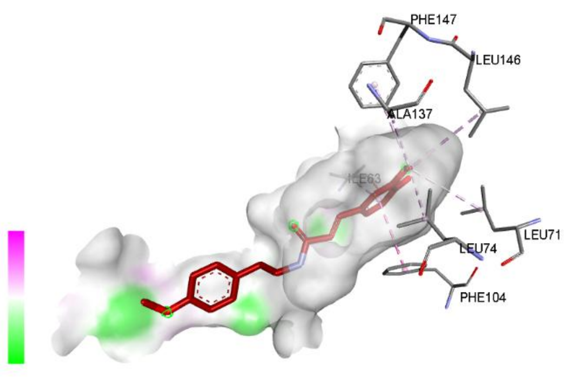

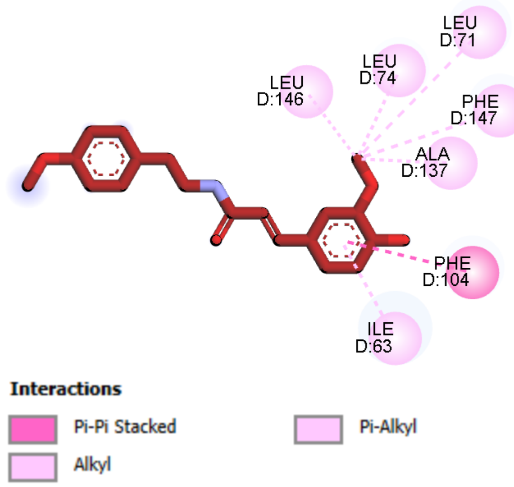

2`-Hydroxy- 3``-methyoxymoupinamide

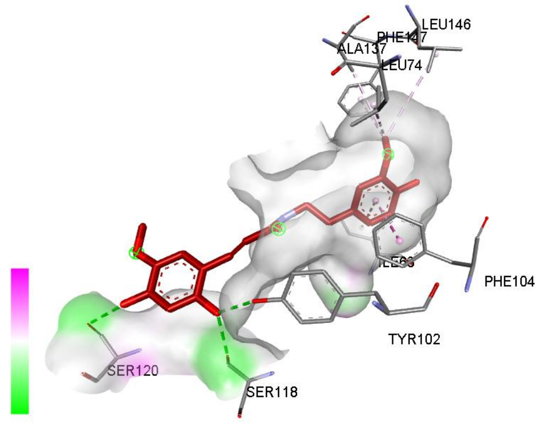

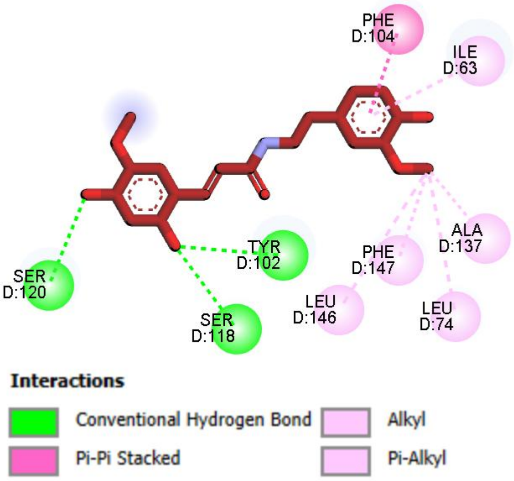

***N-Trans*-feruloyl-3-*O*-methyldopamine; *N-trans*-feruloyl-4`-*O*-methyldopamine**

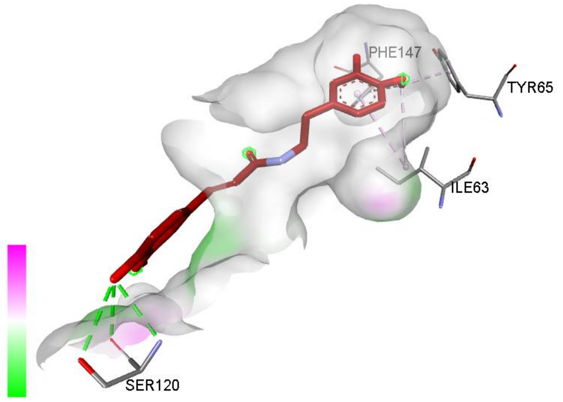

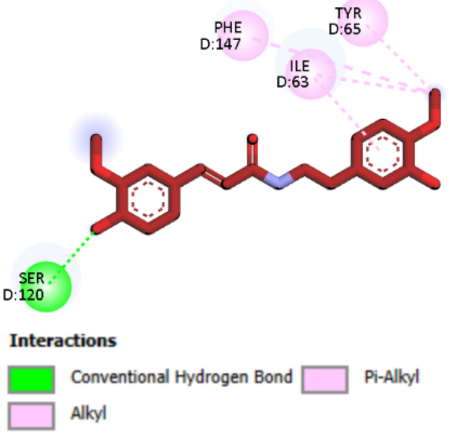

***N*-(3`,4`-Dimethoxy-cinnamoyl)-norepinephrine**

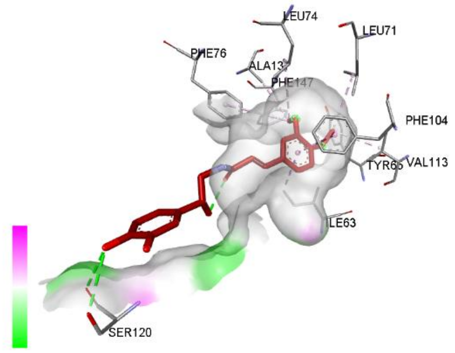

<table>
<tr><td></td><td>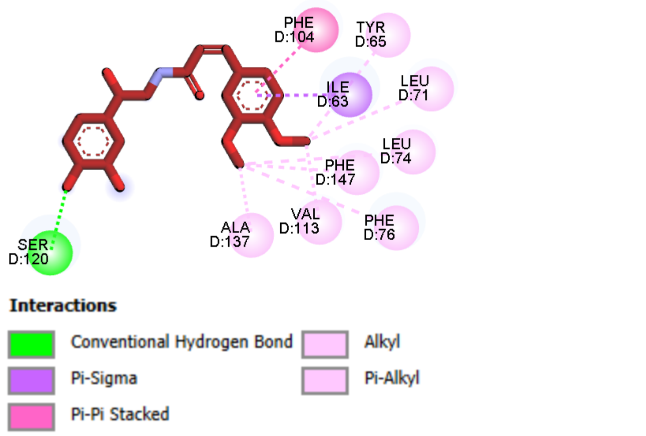
</td></tr>
<tr><td>Erucamide (fatty acid amide)
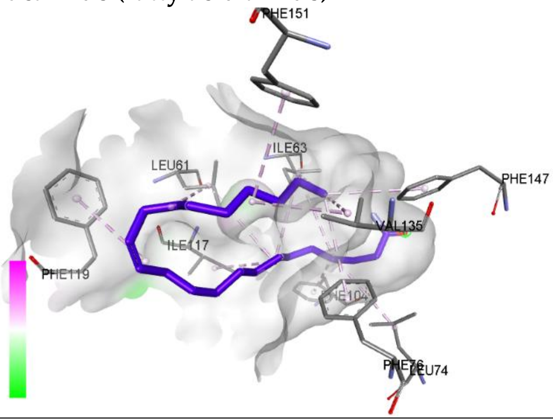
</td><td>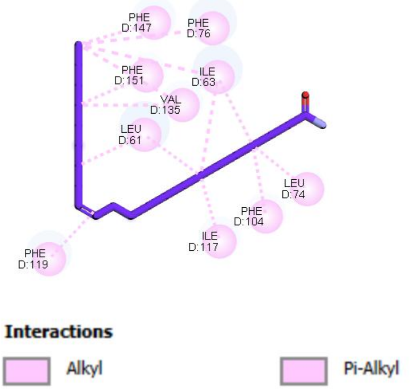
</td></tr>
<tr><td>Commigileadin A
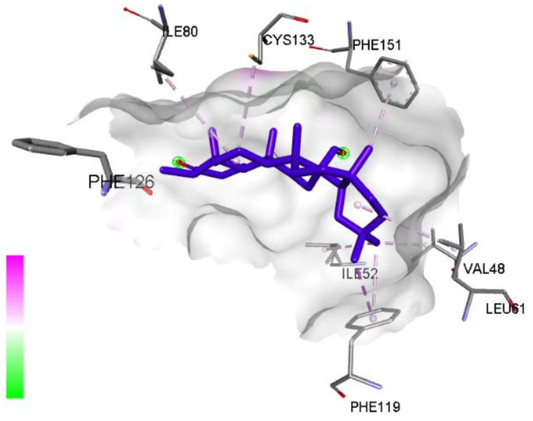
</td><td>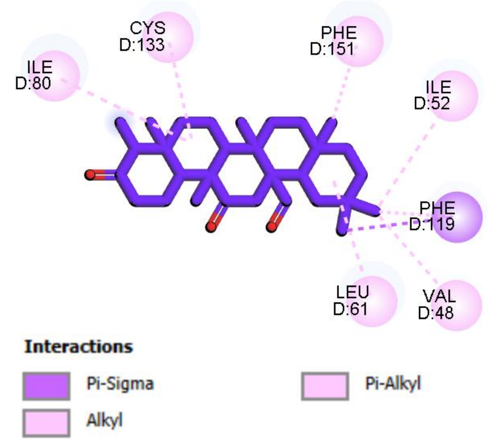
</td></tr>
<tr><td>Urosonic acid
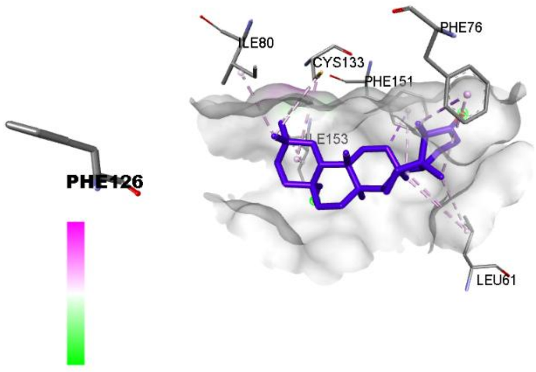
</td><td>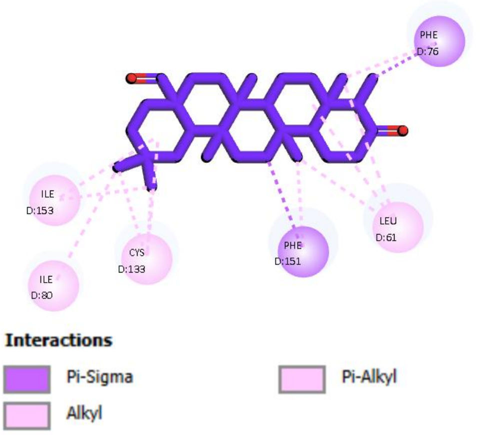
</td></tr>
<tr><td>Canophyllal</td><td></td></tr>
</table>

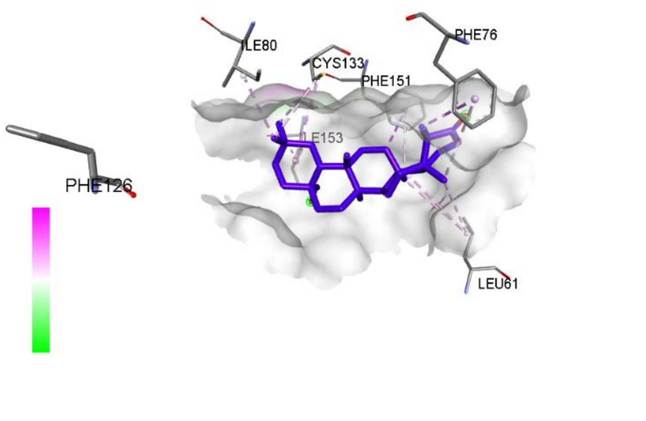

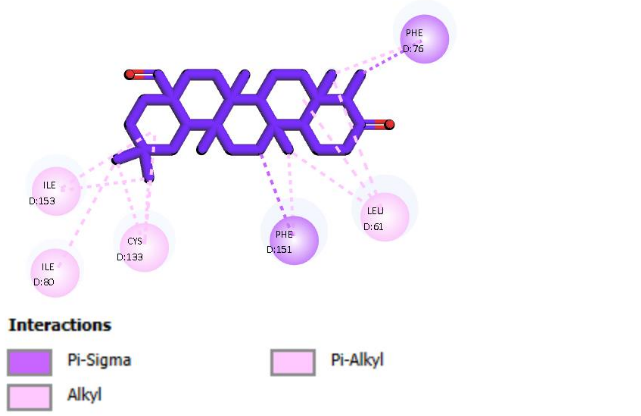

Amasterol

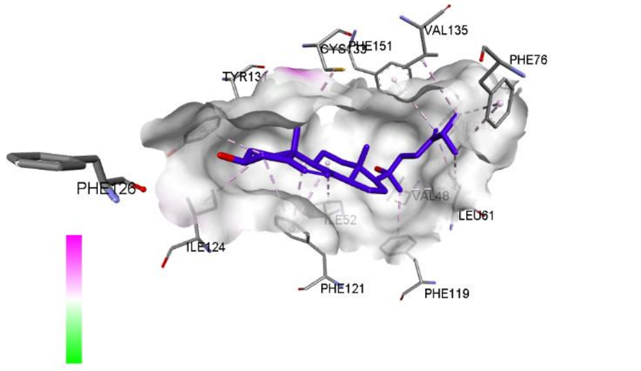

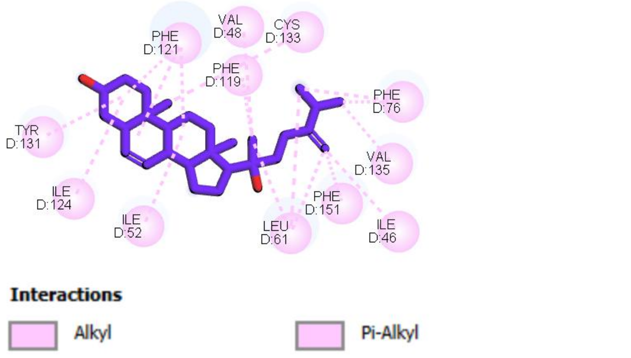

Stigmasterol

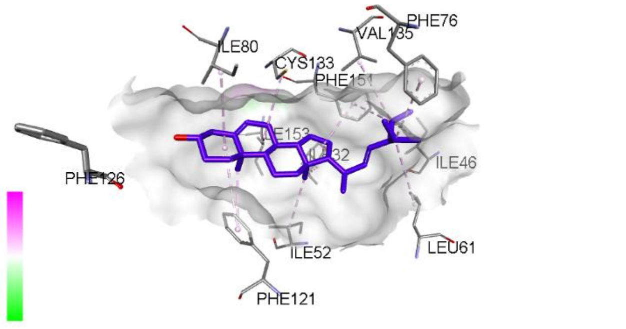

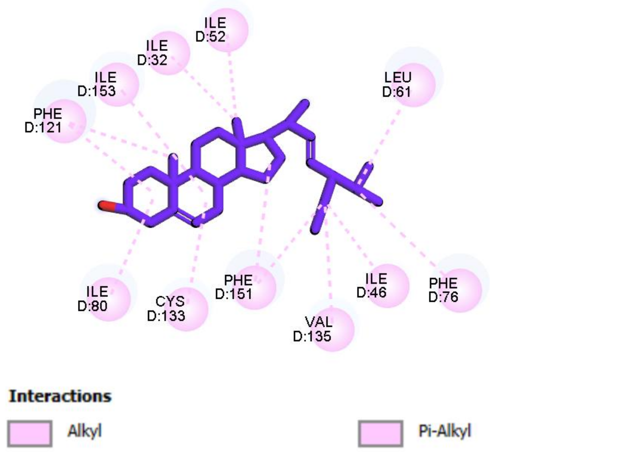

Figure S24. 2D (right) and 3D (left) visualization structure interaction poses of TLR4/MD-2 active site (PDB ID: 2Z65) with selected key identified compounds in the three investigated extracts.

Table S6. Results of molecular docking of the crystallized ligand and selected key bioactive compounds form investigated plants in conjunction with TLR4 (PDBID: 2Z65).

No.	Compound / Class	Structure	S score	RMSD
1.	Eritoran<br>(PDB ID: E55)		**-10.1264**	**1.59**
2.	Isorhamnetin	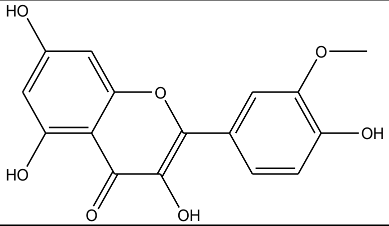	-6.1780	0.897
3.	Luteolin	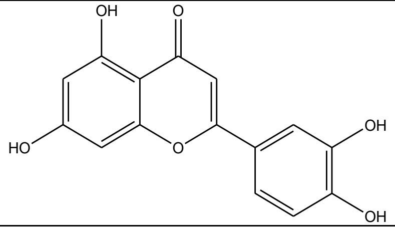	-5.8986	1.1742
4.	Kaempferide	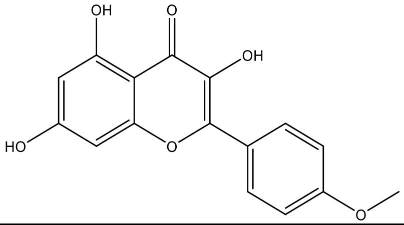	-5.9227	1.6531
5.	Kaempferol	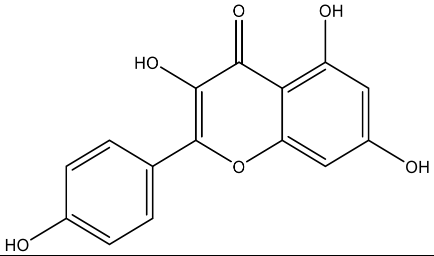	-5.6084	1.4472
6.	Hesperetin	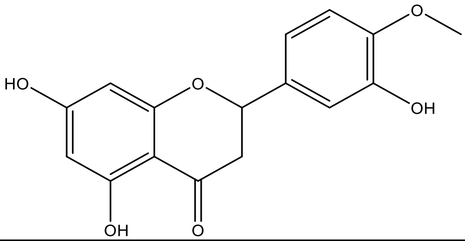	-5.6914	1.8325
7.	Quercetin	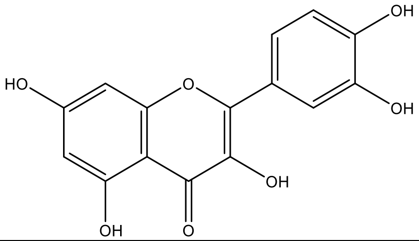	-5.7116	1.1216
8.	Myricetin	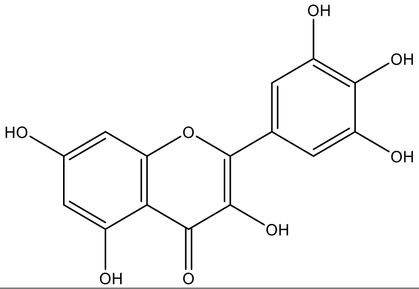	-5.5462	1.7263

9.	Taxifolin	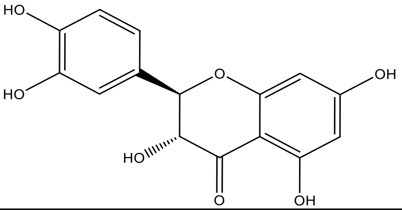	-5.5802	1.2731
10.	Acacetin	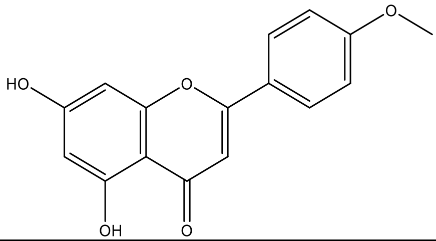	-5.8499	1.5719
11.	Apigenin	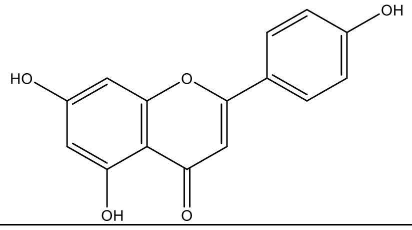	-5.7053	1.1408
12.	Epicatechin	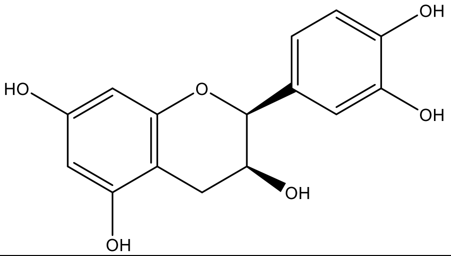	-5.4747	1.4616
13.	(+)-catechin	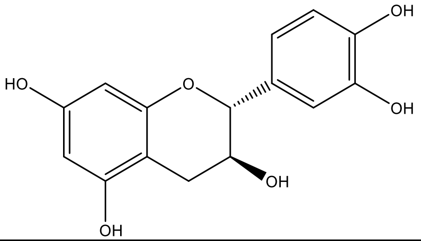	-5.4221	1.2119
14.	Peonidin	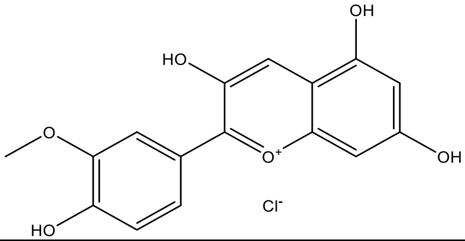	-5.3486	0.9335
15.	Delphinidin	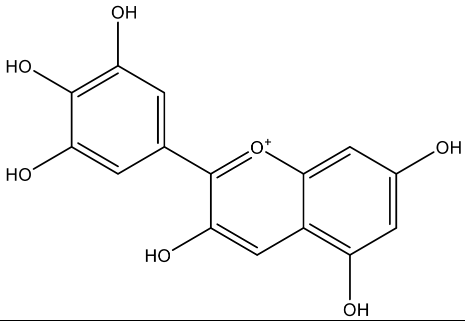	-5.5597	0.9876
16.	Cyanidin	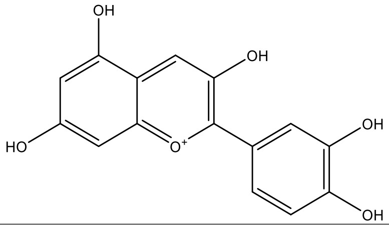	-5.7214	1.0590

17.	Syringetin	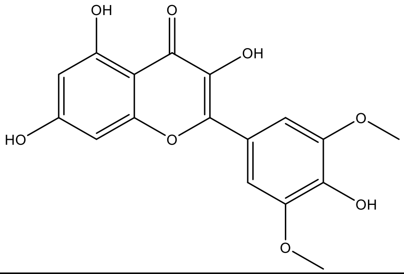	-6.4448	1.3414
18.	Daidzein-8-*C*-glucoside	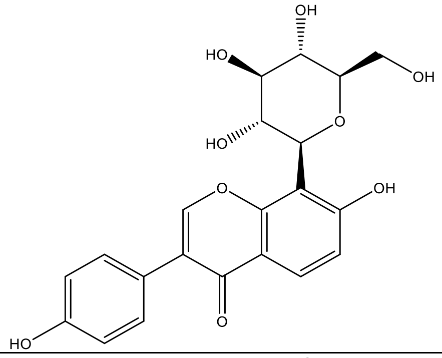	-6.0153	1.5229
19.	Luteolin-8-*C*-glucoside; Orientin	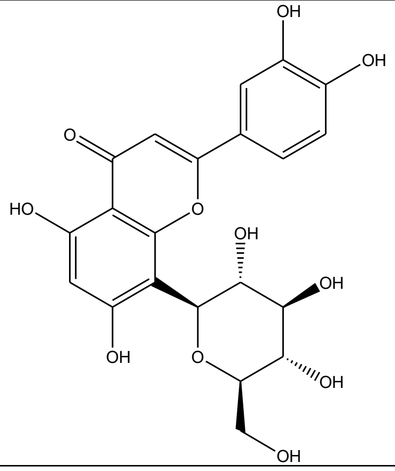	-5.8711	0.9944
20.	*N*-Feruloyltyramine (Moupinamide)	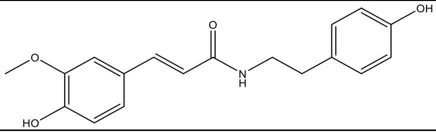	-6.5013	1.2544
21.	*N*-Caffeoyltyramine	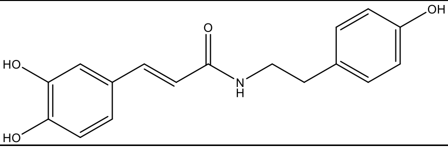	-6.1018	1.7405
22.	(*E*)-3-(4-Hydroxy-3-methoxyphenyl)-*N*-(4-methoxyphenethyl)acrylamide	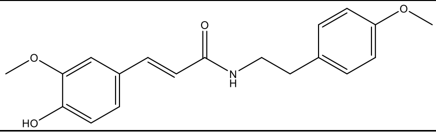	-6.5671	1.6482
23.	2`-Hydroxy- 3``-methyoxymoupinamide	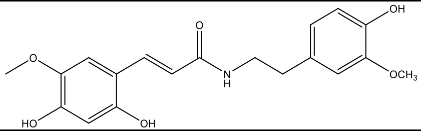	-6.5108	1.3620
24.	*N-Trans*-feruloyl-3-*O*-methyldopamine; *N-trans*-feruloyl-4`-*O*-methyldopamine	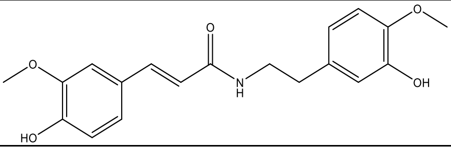	-6.6437	0.7423
25.	*N*-(3`,4`-Dimethoxy-cinnamoyl)-norepinephrine	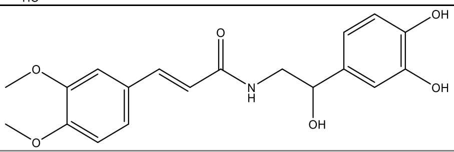	-6.1253	1.0882

26.	Erucamide (fatty acid amide)	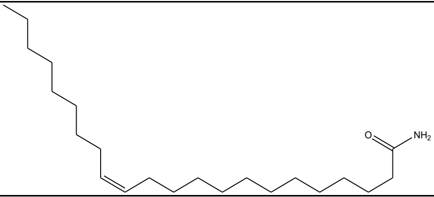	-7.1364	1.2028
27.	Chlorogenic acid	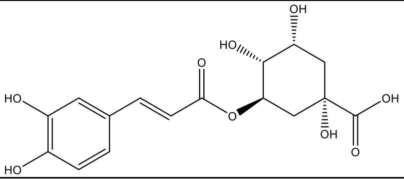	-5.6011	1.4951
28.	Scopoletin (coumarin)	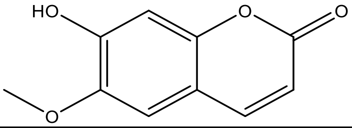	-4.8262	0.9002
29.	Esculetin	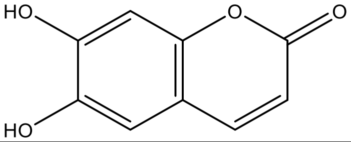	-4.7707	1.2438
30.	Daphnetin	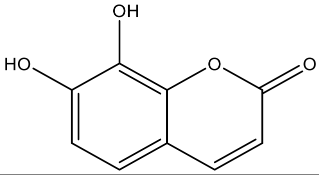	-4.3446	1.2056
31.	Oleanonic aldehyde	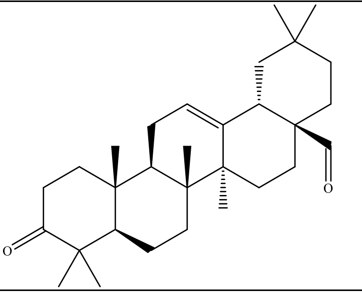	-5.9376	1.5547
32.	11-Oxo-oleanonic acid	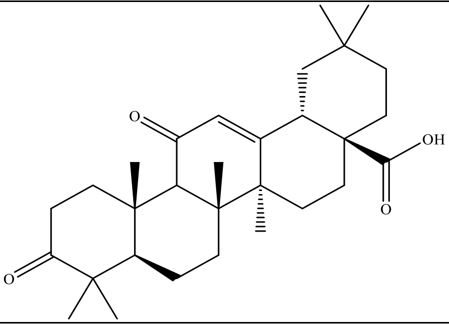	-5.9585	1.6214
33.	11-Oxo-ursonic acid	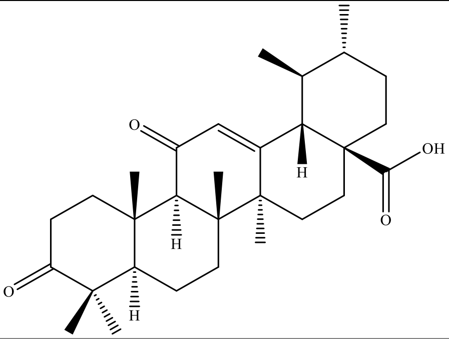	-5.9414	1.2318

34.	Oleanonic acid	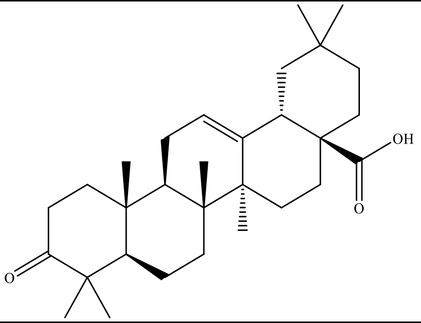	-5.6671	1.0697
35.	Commigileadin A	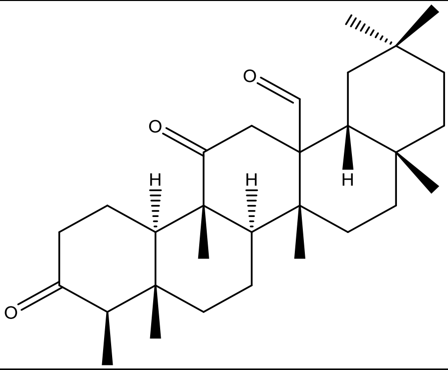	-6.6811	1.4608
36.	Urosonic acid	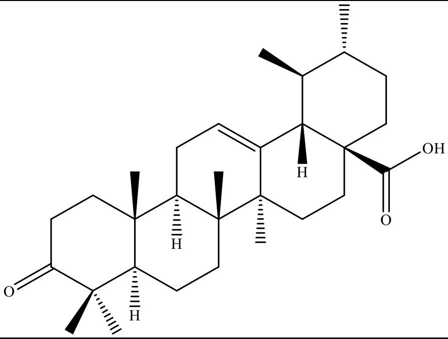	-6.3571	1.1278
37.	Canophyllal	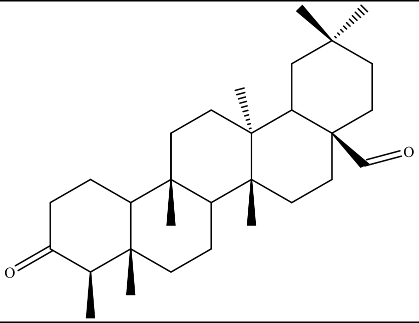	-6.2468	1.4735
38.	Urosonic aldehyde	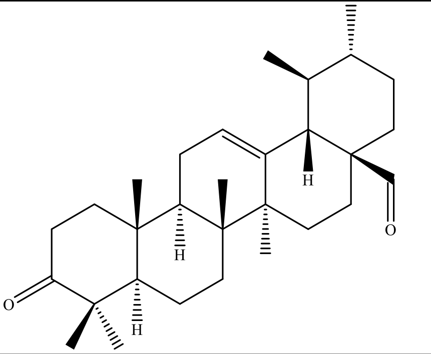	-4.5471	1.0947

39.	Salsolic acid; 3β,6α,24-Trihydroxyolean-12-en-28-oic acid	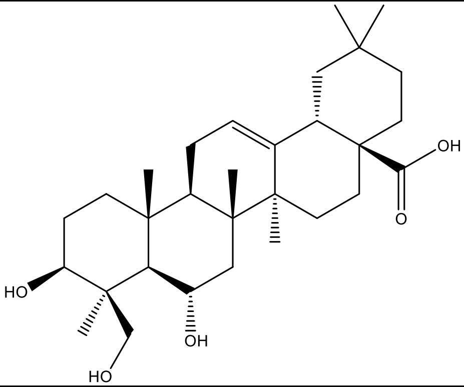	No results	
40.	Salsolin A; 3β,11β,24,30-Tetrahydroxyolean-12-en-28-oic acid	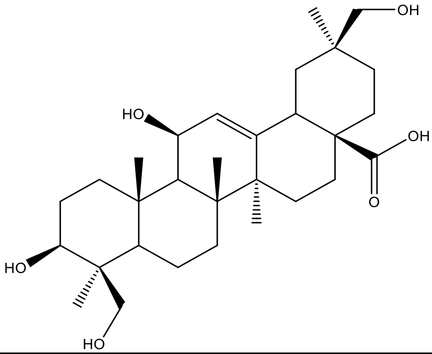	No results	
41.	Salsolin B; 2α,3β,23,24-Tetrahydroxyurs-12-en-28-oic acid	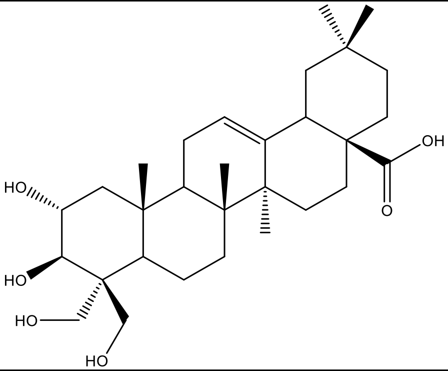	-5.4991	1.6766
42.	Amasterol	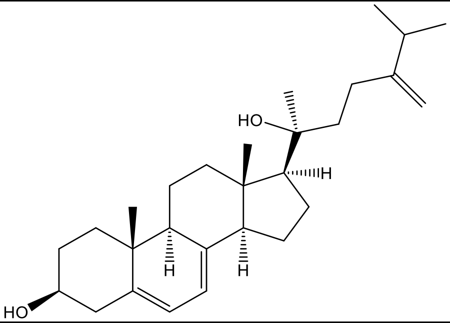	-6.6611	1.6326
43.	Stigmasterol	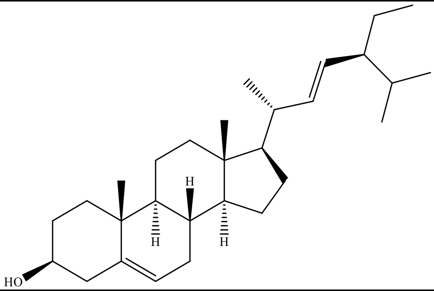	-6.7817	1.1190

Luteolin

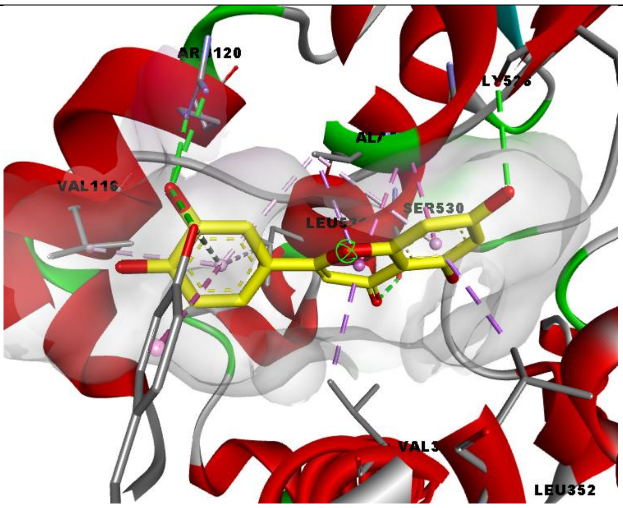

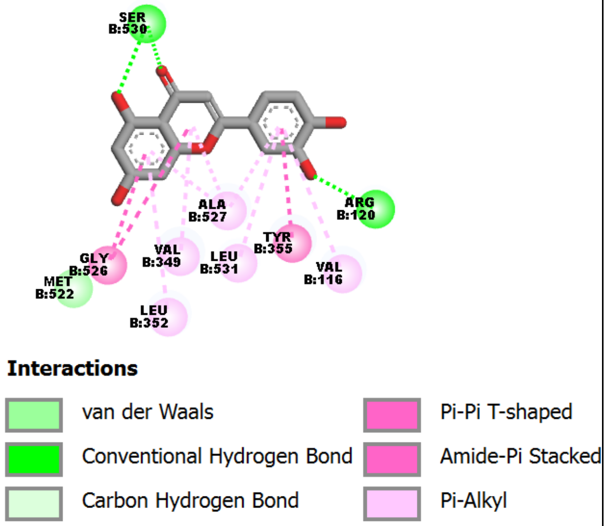

Kaempferide

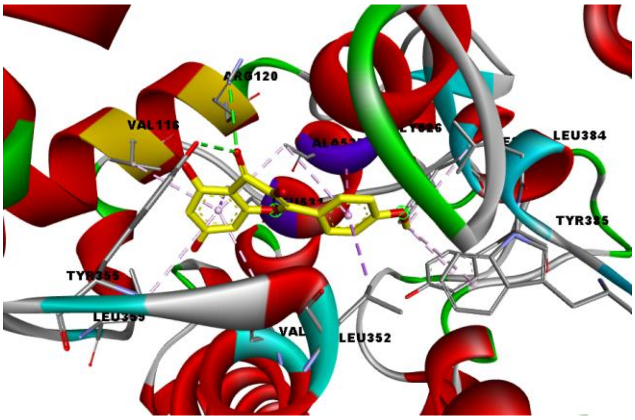

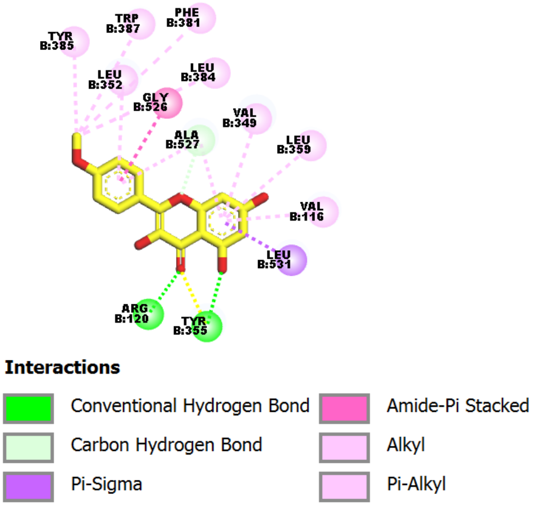

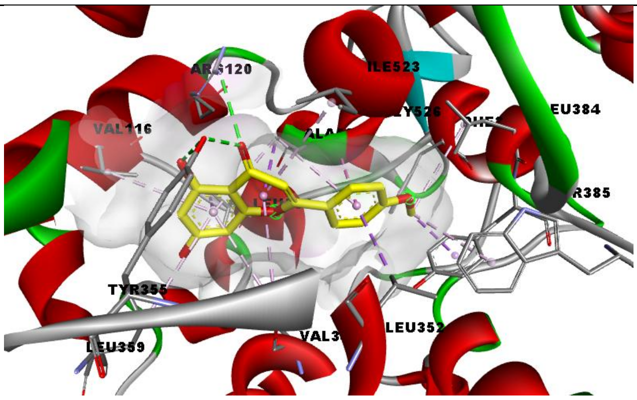

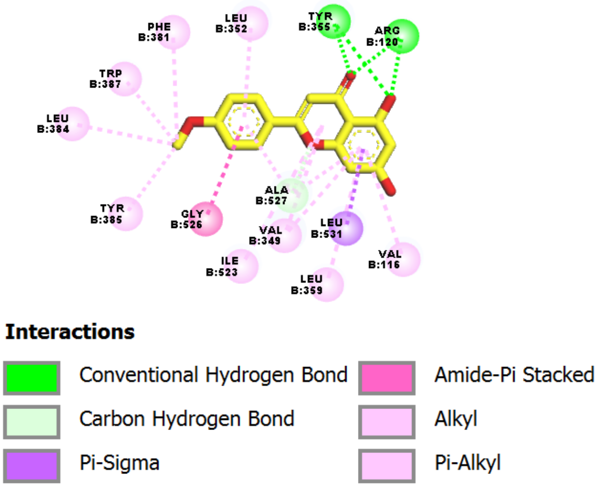

Acacetin

Peonidin

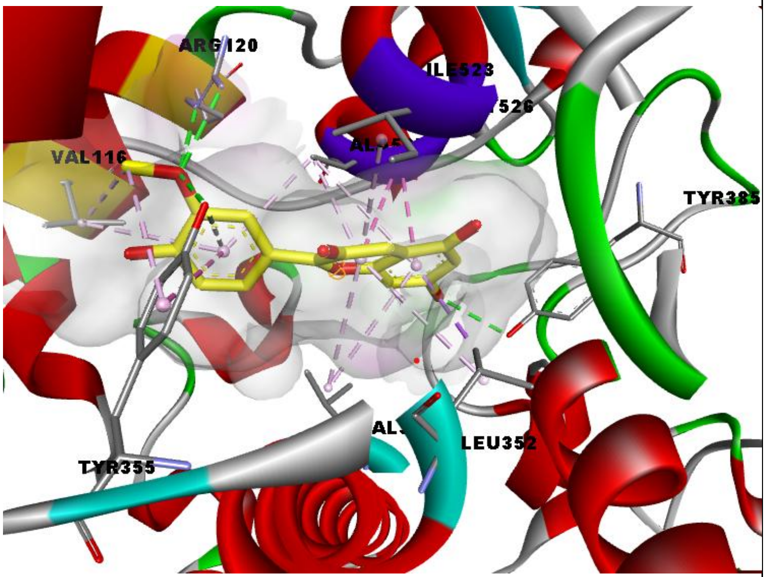

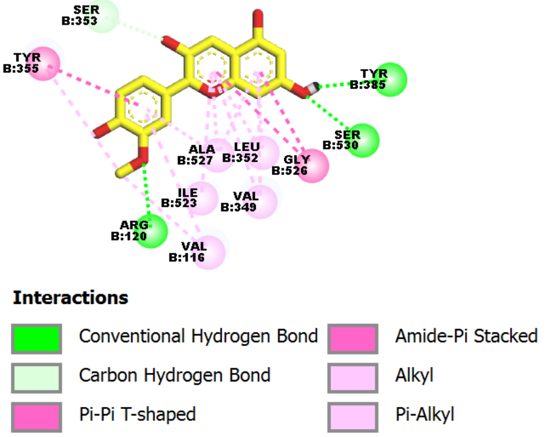

Syringetin

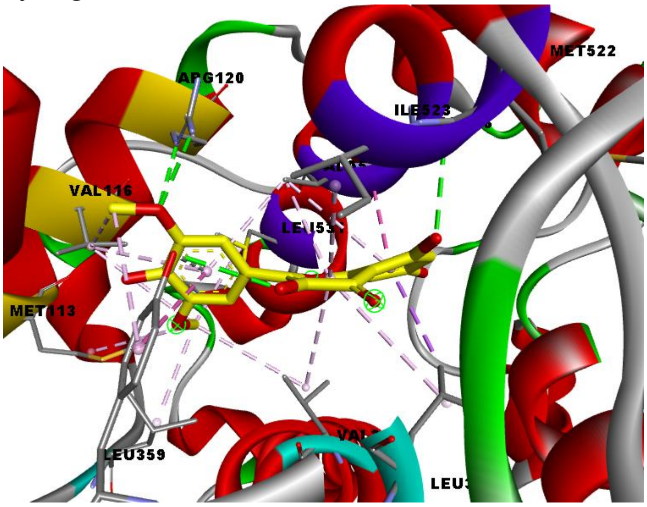

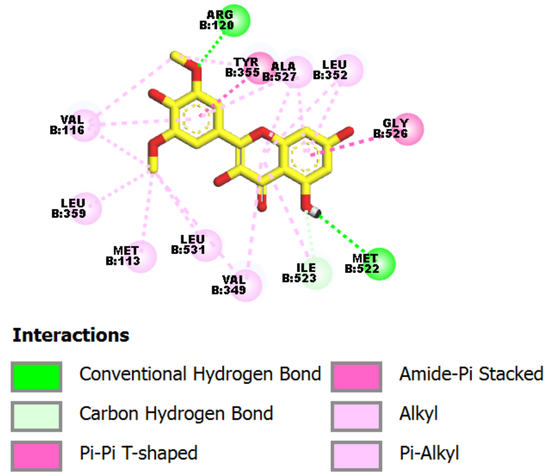

***N*-Feruloyltyramine (Moupinamide)**

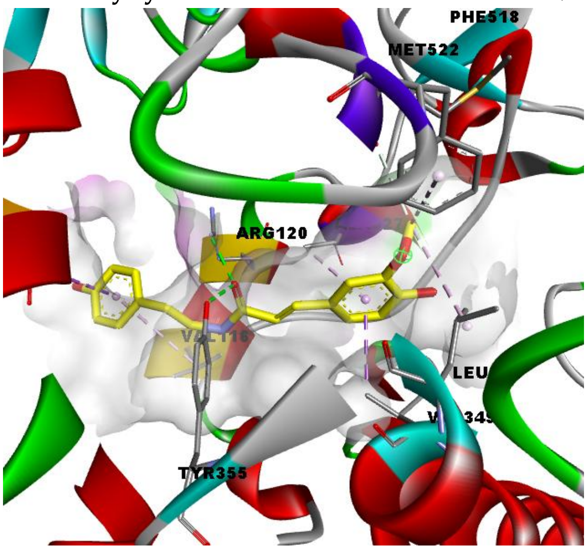

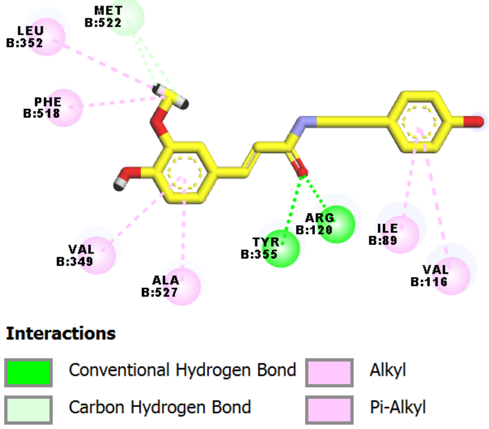

N-Caffeoyltyramine

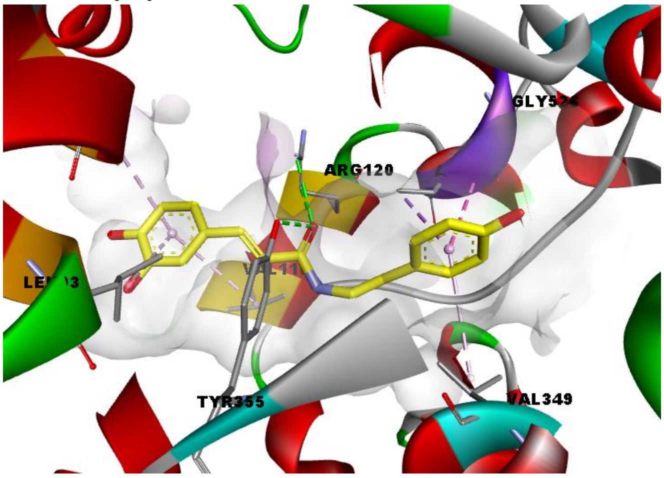

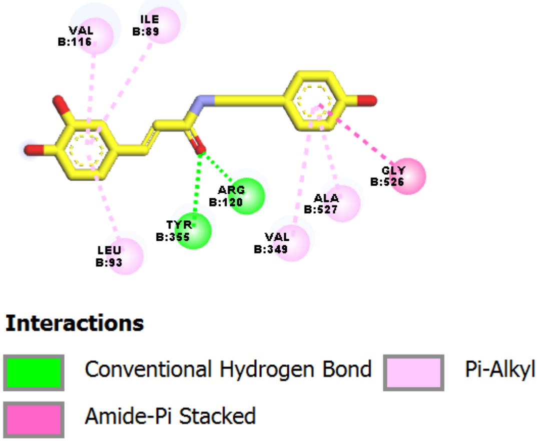

(*E*)-3-(4-Hydroxy-3-methoxyphenyl)-*N*-(4-methoxyphenethyl)acrylamide

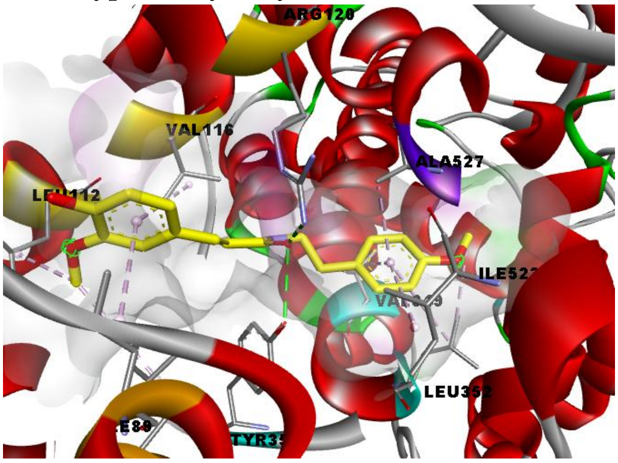

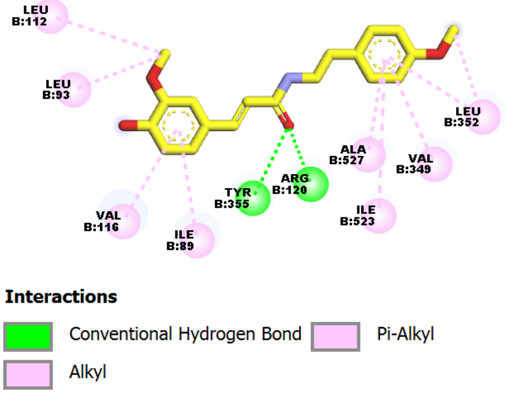

2`-Hydroxy- 3``-methyoxymoupinamide

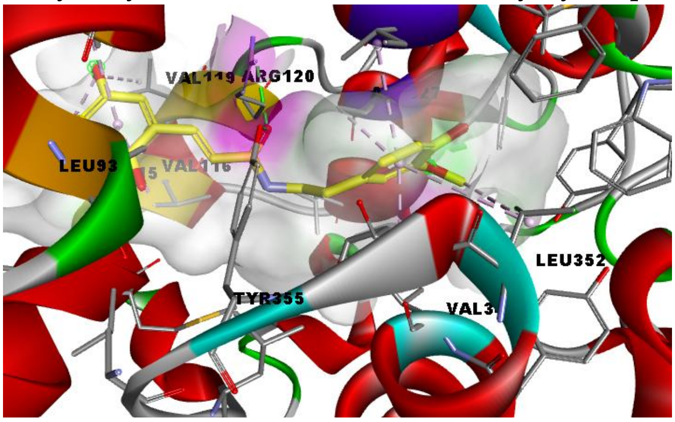

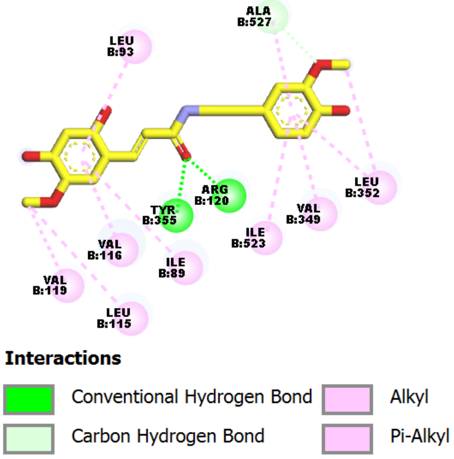

N-Trans-feruloyl-3-*O*-methyldopamine<br><br>*N-trans*-feruloyl-4`-*O*-methyldopamine<br>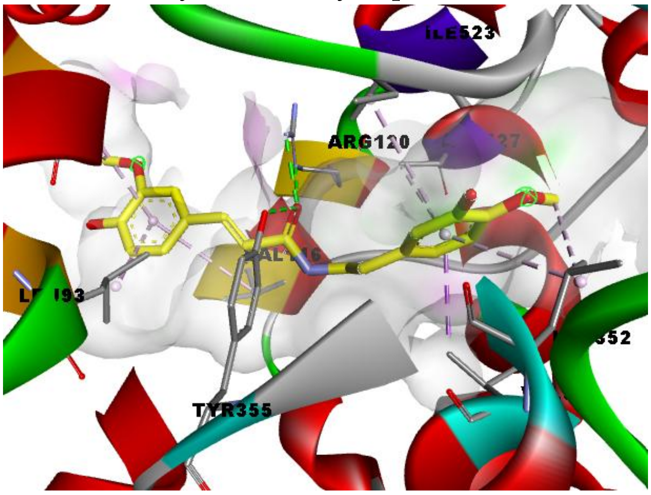	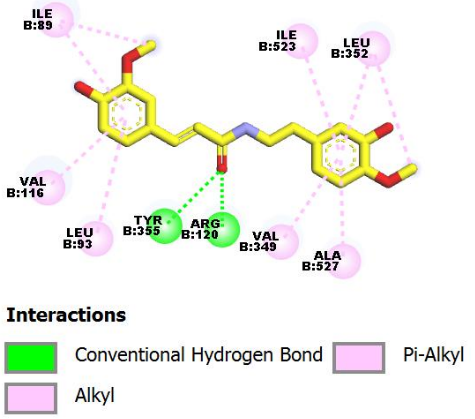
Chlorogenic acid<br>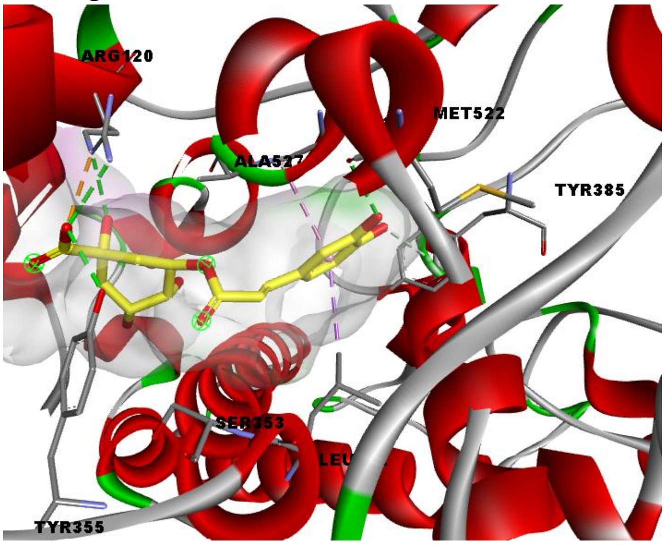	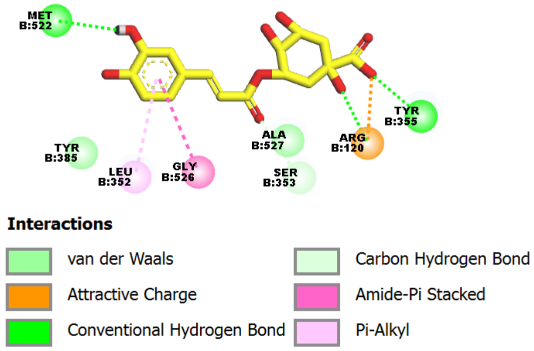
Scopoletin (coumarin)<br>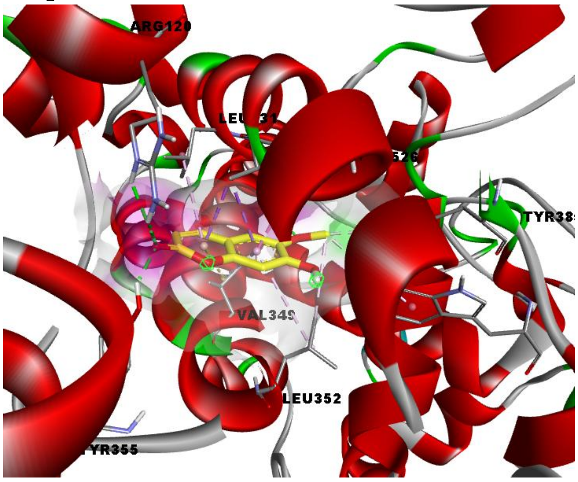	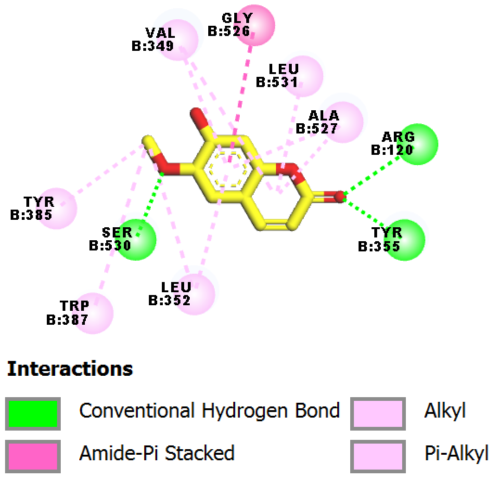

Daphnetin

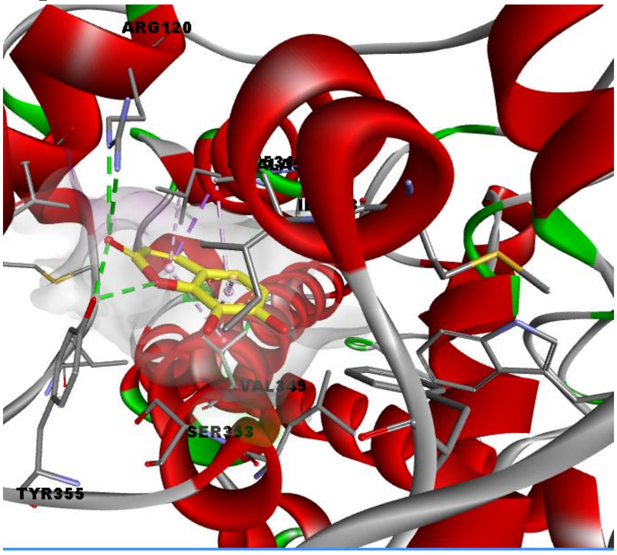

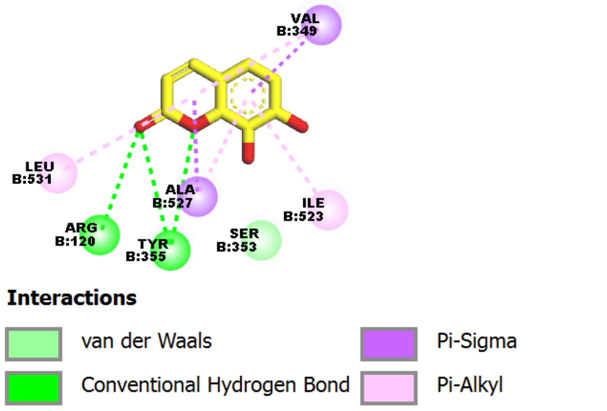

Figure S25. 2D (right) and 3D (left) visualization structure interaction poses of COX-1 (PDB: 1Q4G) with selected key identified compounds in the three investigated extracts.

Luteolin

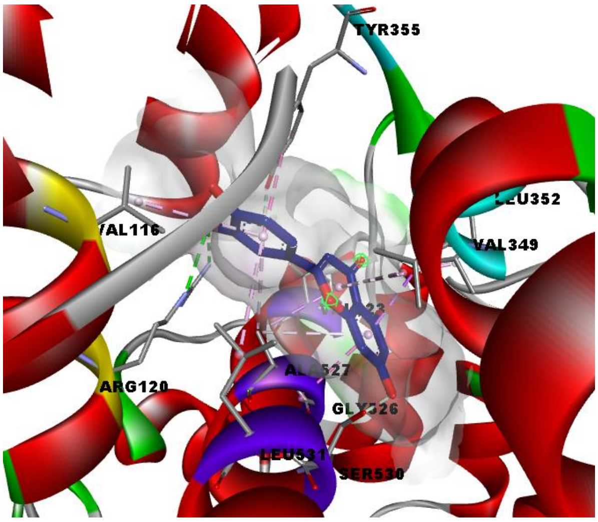

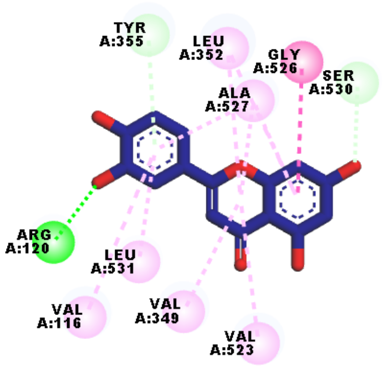

Interactions

- Conventional Hydrogen Bond
- Carbon Hydrogen Bond
- Pi-Donor Hydrogen Bond
- Pi-Pi T-shaped
- Amide-Pi Stacked
- Pi-Alkyl

Kaempferide

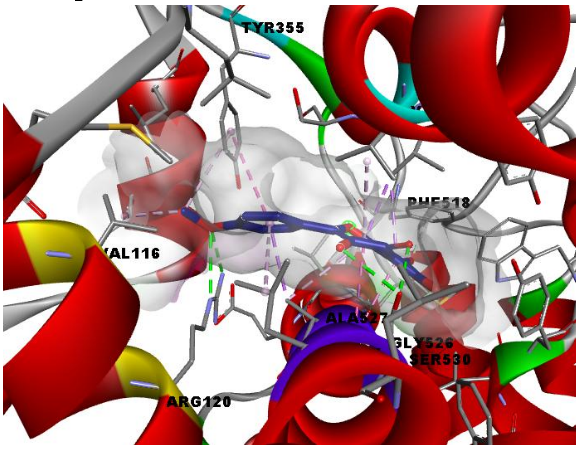

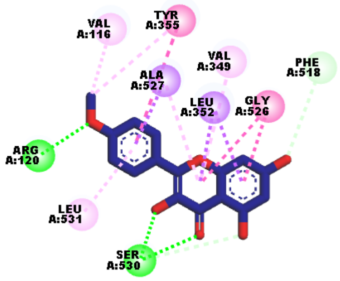

Interactions

- Conventional Hydrogen Bond
- Carbon Hydrogen Bond
- Pi-Donor Hydrogen Bond
- Pi-Sigma
- Pi-Pi T-shaped
- Amide-Pi Stacked
- Alkyl
- Pi-Alkyl

Acacetin

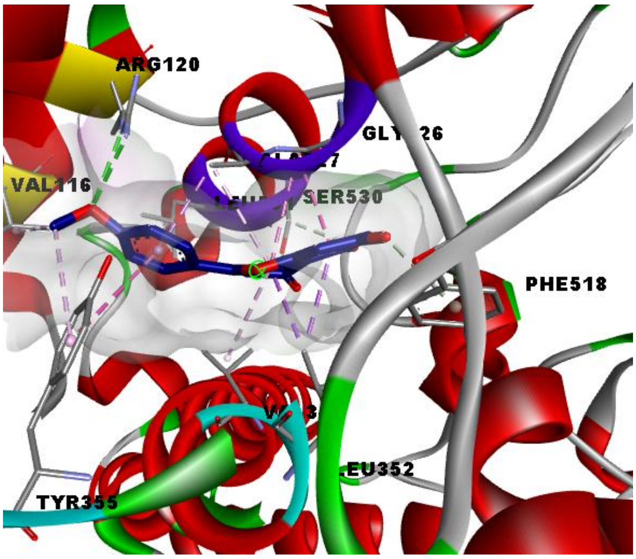

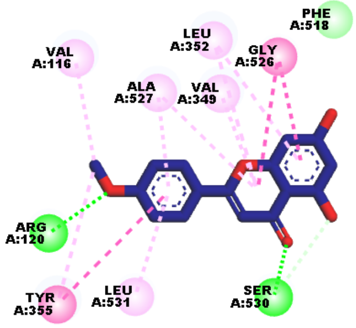

Interactions

- van der Waals
- Conventional Hydrogen Bond
- Carbon Hydrogen Bond
- Pi-Pi T-shaped
- Amide-Pi Stacked
- Alkyl
- Pi-Alkyl

Peonidin

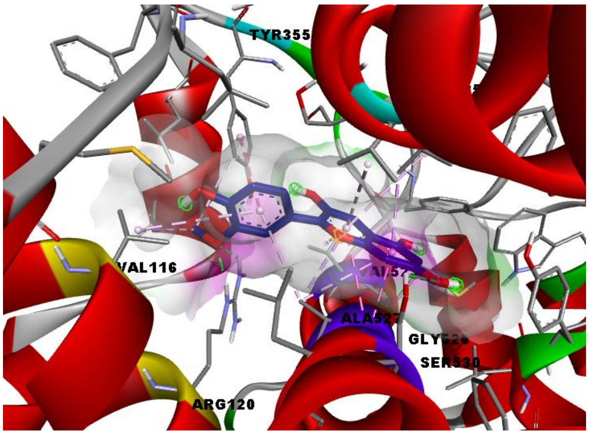

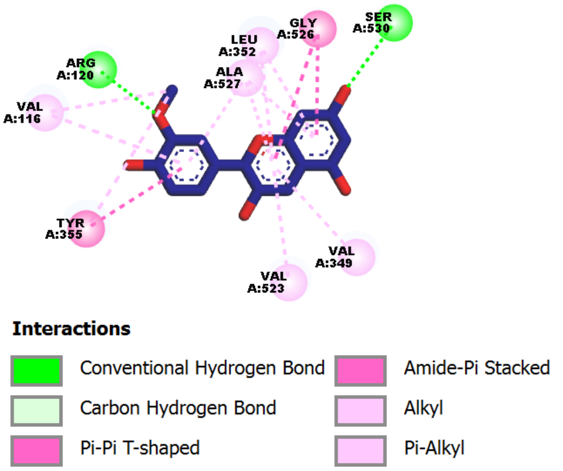

Syringetin

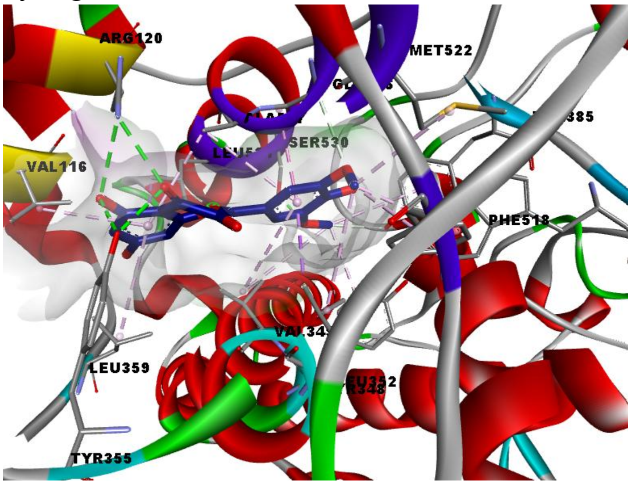

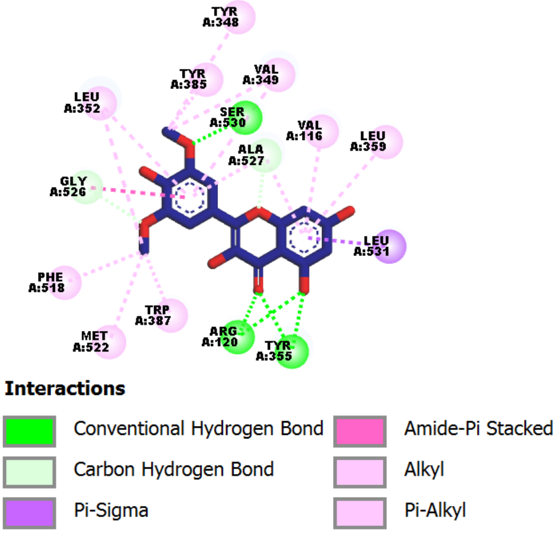

***N*-Feruloyltyramine (Moupinamide)**

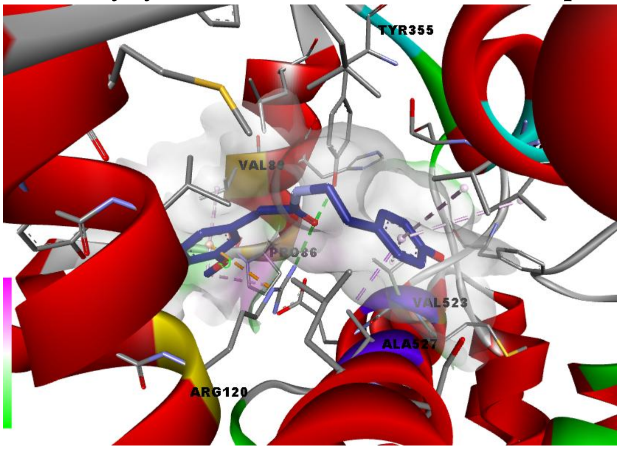

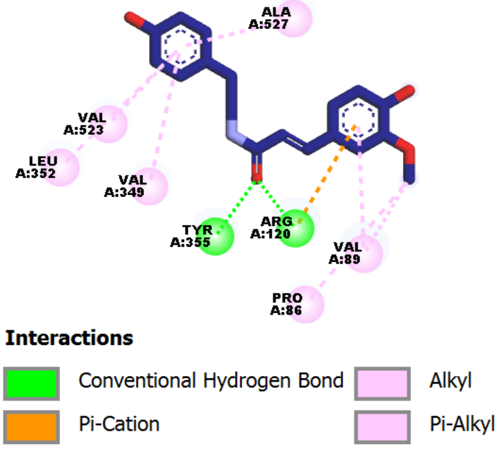

N-Caffeoyltyramine

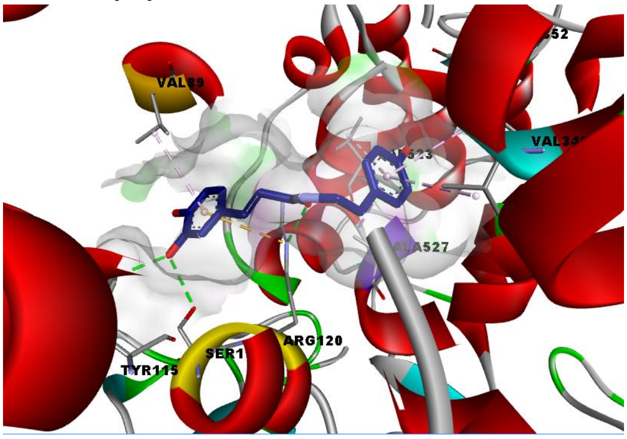

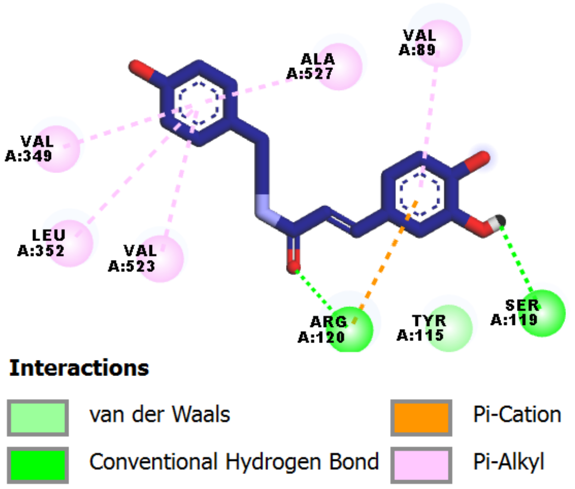

(*E*)-3-(4-Hydroxy-3-methoxyphenyl)-*N*-(4-methoxyphenethyl)acrylamide

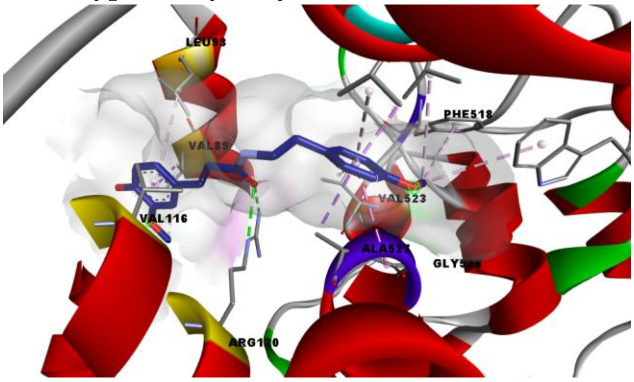

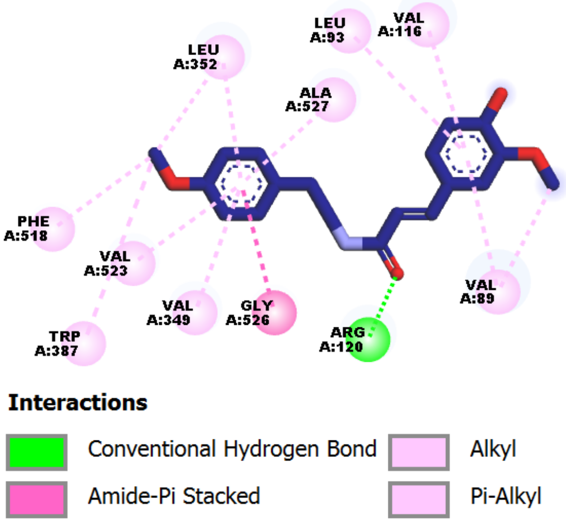

2`-Hydroxy- 3``-methyoxymoupinamide

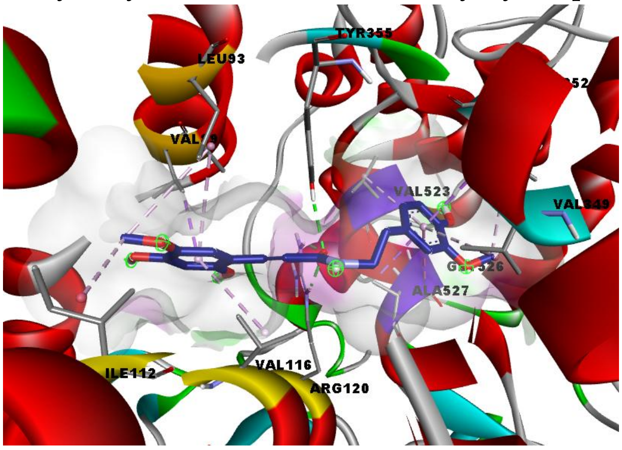

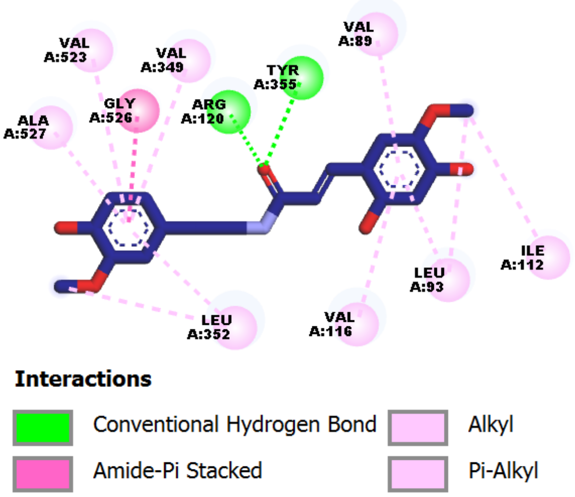

<table>
<tr>
<td>

N-Trans-feruloyl-3-*O*-methyldopamine

N-trans-feruloyl-4`-*O*-methyldopamine

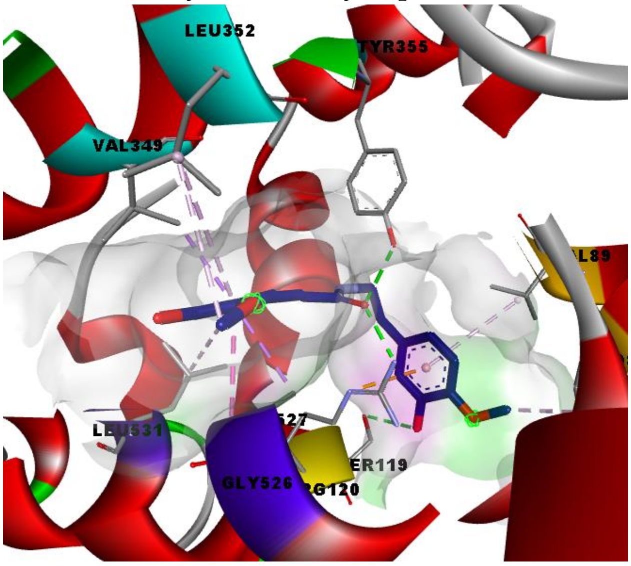

</td>
<td>

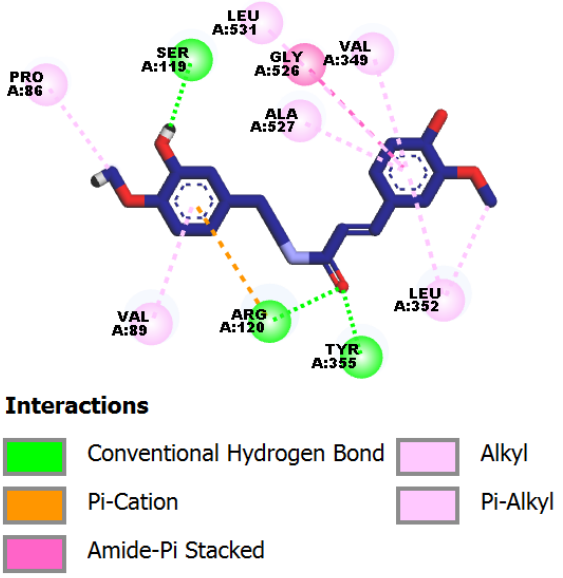

</td>
</tr>
<tr>
<td>

Chlorogenic acid

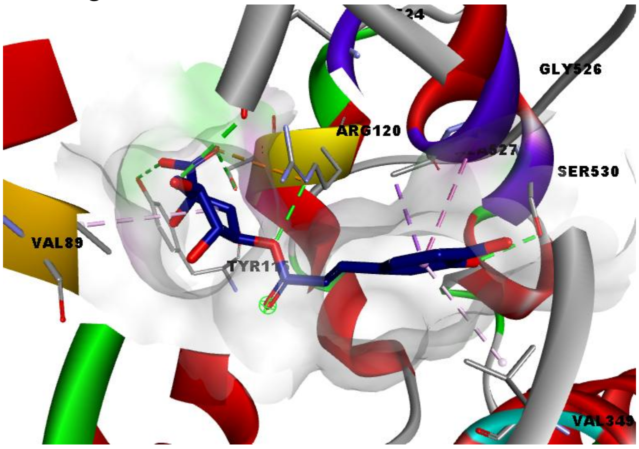

</td>
<td>

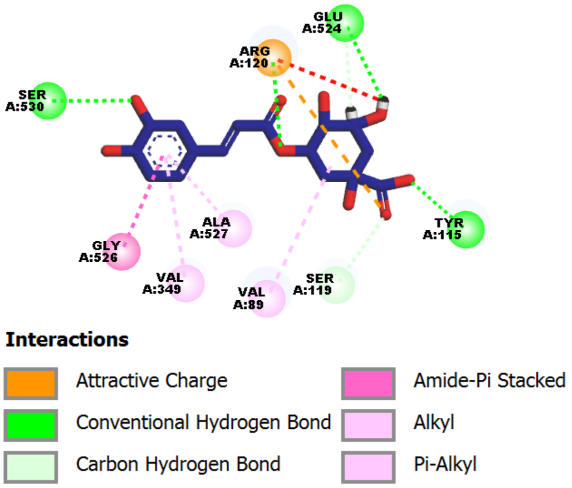

</td>
</tr>
<tr>
<td>

Scopoletin (coumarin)

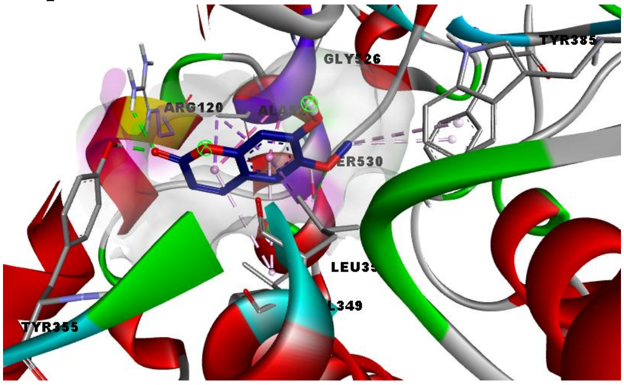

</td>
<td>

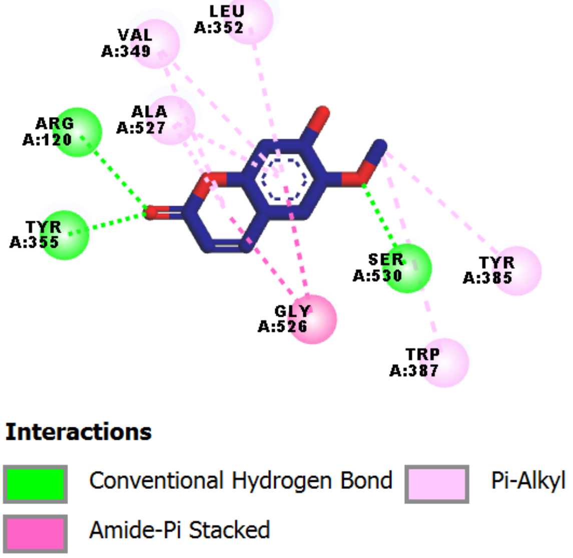

</td>
</tr>
</table>

Daphnetin

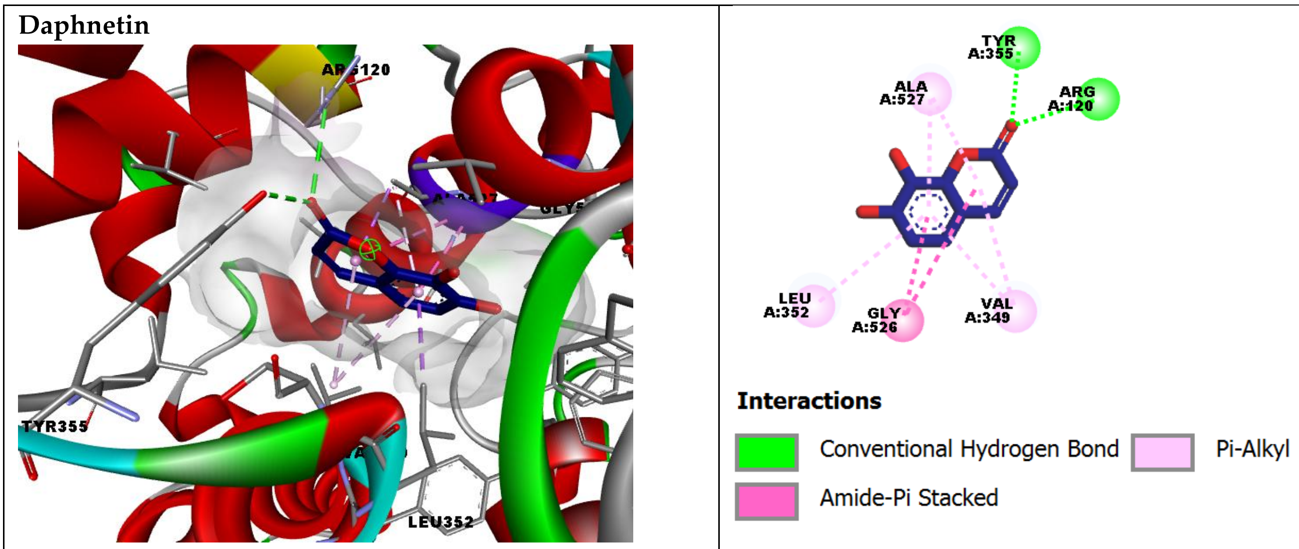

Figure S26. 2D (right) and 3D (left) visualization structure interaction poses of COX-2 (PDB: 3NT1) with selected key identified compounds in the three investigated extracts.

References

1. Elbana, R.M.; Taie, H.A.; Moustafa, A.M.Y.; Marzouk, M. LC-MS/MS analyses of *Khaya grandifoliola* and *in vitro* Antioxidant Activity and Cytotoxicity of *Khaya senegalensis* and *Khaya grandifoliola* against Ehrlich Ascites Carcinoma Cells. *Egypt. J. Chem.* **2024**, *67*, 309-325. https://doi.org/10.21608/ejchem.2024.259569.9124
2. Neumann, S.; Meier, R.; Wenk, M.; Elapavalore, A.; Nishioka, T.; Schulze, T.; Stravs, M.; Tsugawa, H.; Matsuda, F.; Schymanski, E.L. MassBank: an open and FAIR mass spectral data resource. *Nucleic Acids Res.* **2026**, *54*, D601-D606. https://doi.org/10.1093/nar/gkaf1193
3. Attia, R.A.; El-Dahmy, S.I.; Abouelenein, D.; Abdel-Ghani, A.E.-S. LC-ESI-MS profile, cytotoxic, antioxidant, insecticidal and antimicrobial activities of wild and *in vitro* propagated *Tanacetum sinaicum* Del. ex DC. *Zagazig J. Pharm. Sci.* **2022**, *31*, 8-21. https://doi.org/10.21608/zjps.2022.157581.1041
4. Yuzuak, S.; Ballington, J.; Li, G.; Xie, D.-Y. High-Performance Liquid Chromatography–Quadrupole Time-of-Flight Tandem Mass Spectrometry-Based Profiling Reveals Anthocyanin Profile Alterations in Berries of *Hybrid Muscadine* Variety FLH 13-11 in Two Continuous Cropping Seasons. *Agronomy* **2024**, *14*, 442. https://doi.org/10.3390/agronomy14030442
5. Abdl Aziz, F.T.; Temraz, A.S.; Hassan, M.A.; Sciences, M. Metabolites profiling by LC-ESI-MS/MS technique and *in-vitro* antioxidant activity of *Bauhinia madagascariensis* Desv. and *Bauhinia purpurea* L. aerial parts cultivated in Egypt: a comparative study. *Al-Azhar Int. J. Pharm. Sci.* **2024**, *4*, 169-188. https://doi.org/10.21608/aijpms.2023.212409.1215
6. Lech, K. Universal analytical method for characterization of yellow and related natural dyes in liturgical vestments from Krakow. *J. Cult. Herit.* **2020**, *46*, 108-118. https://doi.org/10.1016/j.culher.2020.04.011
7. Badawy, S.A.; Hassan, A.R.; Abu Bakr, M.S.; Mohammed, A.E.-S.I. UPLC-qTOF-MS/MS profiling of phenolic compounds in *Fagonia arabica* L. and evaluation of their cholinesterase inhibition potential through *in-vitro* and *in-silico* approaches. *Sci. Rep.* **2025**, *15*, 5244. https://doi.org/10.1038/s41598-025-86227-0
8. Shi, F.; Pan, H.; Lu, Y.; Ding, L. An HPLC–MS/MS method for the simultaneous determination of luteolin and its major metabolites in rat plasma and its application to a pharmacokinetic study. *J. Sep. Sci.* **2018**, *41*, 3830-3839. https://doi.org/10.1002/jssc.201800585
9. Truchado, P.; Vit, P.; Heard, T.A.; Tomás-Barberán, F.A.; Ferreres, F. Determination of interglycosidic linkages in *O*-glycosyl flavones by high-performance liquid chromatography/photodiode-array

detection coupled to electrospray ionization ion trap mass spectrometry. Its application to *Tetragonula carbonaria* honey from Australia. *Rapid Commun Mass Spectrom* **2015**, *29*, 948-954. https://doi.org/10.1002/rcm.7184
10. Lee, J.-T.; Pao, L.-H.; Hsieh, C.-D.; Huang, P.-W.; Hu, O.Y.-P. Development and validation of an LC-MS/MS method for simultaneous quantification of hesperidin and hesperetin in rat plasma for pharmacokinetic studies. *Anal. Methods.* **2017**, *9*, 3329-3337. https://doi.org/10.1039/C7AY00051K
11. Ancillotti, C.; Ulaszewska, M.; Mattivi, F.; Del Bubba, M. Untargeted metabolomics analytical strategy based on liquid chromatography/electrospray ionization linear ion trap quadrupole/orbitrap mass spectrometry for discovering new polyphenol metabolites in human biofluids after acute ingestion of *vaccinium myrtillus* berry supplement. *J. Am. Soc. Mass Spectrom.* **2018**, *30*, 381-402. https://doi.org/10.1007/s13361-018-2111-y
12. Emir, A.; Emir, C.; Yıldırım, H. Characterization of phenolic profile by LC-ESI-MS/MS and enzyme inhibitory activities of two wild edible garlic: *Allium nigrum* L. and *Allium subhirsutum* L. *J. Food Biochem.* **2020**, *44*, e13165. https://doi.org/10.1111/jfbc.13165
13. Wood, K.V.; Bonham, C.C.; Miles, D.; Rothwell, A.P.; Peel, G.; Wood, B.C.; Rhodes, D. Characterization of betaines using electrospray MS/MS. *Phytochemistry* **2002**, *59*, 759-765. https://doi.org/10.1016/S0031-9422(02)00049-3
14. Van der Doelen, G.A.; Van den Berg, K.J.; Boon, J.J.; Shibayama, N.; De La Rie, E.R.; Genuit, W.J.L. Analysis of fresh triterpenoid resins and aged triterpenoid varnishes by high-performance liquid chromatography–atmospheric pressure chemical ionisation (tandem) mass spectrometry. *J. Chromatogr. A.* **1998**, *809*, 21-37. https://doi.org/10.1016/S0021-9673(98)00186-1
15. Abdallah, H.; Mohamed, G.; M Ibrahim, S.; Koshak, A.; Alnashri, I.; Alghamdi, A.; Aljohani, A.; Khairy, A. Commigileadin A: A new triterpenoid from *Commiphora gileadensis* aerial parts. *Pharmacogn. Mag.* **2022**, *18*. https://doi.org/10.4103/pm.pm_118_21
16. Abdel-Kader, M.S.; Ibnouf, E.O.; Alqarni, M.H.; AlQutaym, A.S.; Salkini, A.A.; Foudah, A.I. Terpenes from the fresh stems of *Commiphora gileadensis* with antimicrobial activity. *Rec. Nat. Prod.* **2022**, *16*, 605-613. https://doi.org/10.25135/318.2202.2358
17. Rozenberg, R.; Ruibal-Mendieta, N.L.; Petitjean, G.; Cani, P.; Delacroix, D.L.; Delzenne, N.M.; Meurens, M.; Quetin-Leclercq, J.; Habib-Jiwan, J.-L. Phytosterol analysis and characterization in spelt (*Triticum aestivum* ssp. spelta L.) and wheat (*T. aestivum* L.) lipids by LC/APCI-MS. *J. Cereal Sci.* **2003**, *38*, 189-197. https://doi.org/10.1016/S0733-5210(03)00022-5
18. Schymanski, E.L.; Jeon, J.; Gulde, R.; Fenner, K.; Ruff, M.; Singer, H.P.; Hollender, J. Identifying small molecules via high resolution mass spectrometry: communicating confidence. *Environ. Sci. Technol.* **2014**, *48*, 2097-2098. https://doi.org/10.1021/es5002105
19. ElNaggar, M.H.; Abdelmohsen, U.R.; Bar, F.M.A.; Kamer, A.A.; Bringmann, G.; Elekhnawy, E. Investigation of bioactive components responsible for the antibacterial and anti-biofilm activities of *Caroxylon volkensii* by LC-QTOF-MS/MS analysis and molecular docking. *RSC adv.* **2024**, *14*, 11388-11399. https://doi.org/10.1039/d4ra01646g
20. Xiang, Y.; Li, Y.B.; Zhang, J.; Li, P.; Yao, Y.Z. [Studies on chemical constituents of *Salsola collina*]. *Zhongguo Zhong yao za zhi = Zhongguo zhongyao zazhi = China journal of Chinese materia medica* **2007**, *32*, 409-413.
21. ElNaggar, M.H.; Eldehna, W.M.; Abourehab, M.A.; Abdel Bar, F.M. The old world salsola as a source of valuable secondary metabolites endowed with diverse pharmacological activities: a review. *J. Enzyme Inhib. Med. Chem.* **2022**, *37*, 2036-2062. https://doi.org/10.1080/14756366.2022.2102005
22. Yahagi, T.; Yamashita, Y.; Daikonnya, A.; Wu, J.-b.; Kitanaka, S. New feruloyl tyramine glycosides from *Stephania hispidula* Y AMAMOTO. *Chem. Pharm. Bull.* **2010**, *58*, 415-417. https://doi.org/10.1248/cpb.58.415
23. Elsharabasy, F.S.; Hosney, A.M. Chemical constituents from the aerial parts of *Salsola inermis*. *Egypt. Pharm. J.* **2013**, *12*, 90-94. https://doi.org/10.7123/01.EPJ.0000428060.24957.95

24. Wang, Z.; Wang, Q.; Zhang, M.; Hu, X.; Ding, G.; Jiang, M.; Bai, G. Cimicifugamide from *Cimicifuga rhizomes* functions as a nonselective β-AR agonist for cardiac and sudorific effects. *Biomed. Pharmacother.* **2017**, *90*, 122-130. https://doi.org/10.1016/j.biopha.2017.03.058
25. Jiang, C.; Gates, P. Systematic characterisation of the fragmentation of flavonoids using high-resolution accurate mass electrospray tandem mass spectrometry. *Molecules* **2024**, *29*, 5246. https://doi.org/10.3390/molecules29225246
26. Ahmad, Z.; Mehmood, S.; Fatima, I.; Malik, A.; Ifzal, R.; Afza, N.; Iqbal, L.; Latif, M.; Nizami, T.A. Structural determination of salsolins A and B, new antioxidant polyoxygenated triterpenes from *Salsola baryosma*, by 1D and 2D NMR spectroscopy. *Magn. Reson. Chem.* **2008**, *46*, 94-98. https://doi.org/10.1002/mrc.2119
27. Ahmad, Z.; Mehmood, S.; Ifzal, R.; Malik, A.; Afza, N.; Rashid, F.; Mahmood, A.; Iqbal, L. Butyrylcholinesterase inhibitory triterpenes from *Salsola baryosma*. *Pol. J. Chem.* **2007**, *81*, 1427-1432.
28. Hegazi, N.M.; El Shabrawy, M.; Aboulthana, W.M.; Ragheb, A.Y.; Ibrahim, L.F.; Marzouk, M.M. UPLC-HRMS/MS Guided Isolation and NMR Investigation of Major Flavonoids from *Enarthrocarpus strangulatus* Boissier (Brassicaceae) with *In Vitro* Enzymes Inhibitory Potential. *Chem. Biodivers.* **2025**, *22*, e202401402. https://doi.org/10.1002/cbdv.202401402
29. Xiong, J.-L.; Ma, N. Transcriptomic and metabolomic analyses reveal that fullerol improves drought tolerance in *Brassica napus* L. *Int. J. Mol. Sci.* **2022**, *23*, 15304. https://doi.org/10.3390/ijms232315304
30. Jahangir, M.; Kim, H.K.; Choi, Y.H.; Verpoorte, R. Health-affecting compounds in Brassicaceae. *Compr. Rev. Food Sci. Food Saf.* **2009**, *8*, 31-43. https://doi.org/10.1111/j.1541-4337.2008.00065.x
31. Chen, Y.-Y.; Lu, H.-Q.; Jiang, K.-X.; Wang, Y.-R.; Wang, Y.-P.; Jiang, J.-J. The flavonoid biosynthesis and regulation in *Brassica napus*: A review. *Int. J. Mol. Sci.* **2022**, *24*, 357. https://doi.org/10.3390/ijms24010357
32. Park, C.H.; Bong, S.J.; Lim, C.J.; Kim, J.K.; Park, S.U. Transcriptome Analysis and Metabolic Profiling of Green and Red Mizuna (*Brassica rapa* L. var. *japonica*). *Foods* **2020**, *9*. https://doi.org/10.3390/foods9081079
33. Tomas, M.; Zhang, L.; Zengin, G.; Rocchetti, G.; Capanoglu, E.; Lucini, L.J.F.R.I. Metabolomic insight into the profile, in vitro bioaccessibility and bioactive properties of polyphenols and glucosinolates from four Brassicaceae microgreens. **2021**, *140*, 110039.
34. Ressurreição, S.; Salgueiro, L.; Figueirinha, A. *Diplotaxis muralis* as an Emerging Food Crop: Chemical Composition, Nutritional Profile and Antioxidant Activities. *Plants* **2025**, *14*, 844. https://doi.org/10.3390/plants14060844
35. Lucarini, M.; Di Cocco, M.E.; Raguso, V.; Milanetti, F.; Durazzo, A.; Lombardi-Boccia, G.; Santini, A.; Delfini, M.; Sciubba, F. NMR-Based Metabolomic Comparison of *Brassica oleracea* (Var. italica): Organic and Conventional Farming. *Foods* **2020**, *9*. https://doi.org/10.3390/foods9070945
